# Supplementary material for: High‐resolution definition of humoral immune response correlates of effective immunity against HIV
Source: Mol Syst Biol. 2018 Mar 26;14(3):e7881. doi: 10.15252/msb.20177881 (PMC5868198; doi:10.15252/msb.20177881)
Supplement: Supplementary file 1 — Appendix [file MSB-14-e7881-s001.pdf]

**Appendix**


| | |
|---|---|
| **Tables** | |
| Table S1 | Antibody Features |
| **Figures** | |
| Figure S1 | Classification of subject group using Fc Array features |
| Figure S2 | Classification of progression and viremia status using Fc Array features |
| Figure S3 | Classification of viremia status using gp120-specific IgG glycosylation features |
| Figure S4 | Level of IgG glycoforms in aviremic (AV) and viremic (V) subjects |
| Figure S5 | Models of antibody effector function learned from Fc Array features |
| Figure S6 | Models of ADCC learned from Fc Array and gp120-specific IgG glycosylation features |
| Figure S7 | Correlations observed between gp120-specific IgG glycoforms and antibody effector function across functional assays |

**Table S1. Antibody Features**

| **Functions** | **Glycoforms** | **Fc Array** | |
|---|---|---|---|
| ADCD | G2FB | **Antigens** | **Detections** |
| ADCC | G2F | gp120.93TH975 | **FcγR** |
| ADNP | G2B | gp120.96ZM651 | FcγRI |
| CD107a | G2orG1FB | gp120.BAL | FcγRIIa H131 |
| IFNγ | G1F | gp120.BAL.Kif | FcγRIIa R131 |
| MIP1β | G1B | gp120.Chiang.Mai | FcγRIIb |
| | G0FB | gp120.CM | FcγRIIIa F158 |
| | G1 | gp120.CM235 | FcγRIIIa V158 |
| | G0F | gp120.CM244 | FcγRIIIb NA1 |
| | G0B | gp120.CN54 | FcγRIIIb SH |
| | G2S1F | gp120.Du151 | |
| | G2S1B | gp120.Du156.12 | **Complement** |
| | G2S1 | gp120.IIIb | C1q |
| | G1S1F | gp120.JRCSF | MBL |
| | G1S1 | gp120.MN | |
| | G2S2FB | gp120.PVO | **Lectins** |
| | G2S2F | gp120.RSC3 | PNA |
| | G2S2B | gp120.RSC3.delta3711 | SNA |
| | G2S2 | gp120.SF162 | VVL |
| | G0.total | gp120.TRO | LCA |
| | G1.total | gp120.YU2 | |
| | G2.total | gp120.ZM109F | **Subclass** |
| | Fucose.total | gp140.BR29 | Anti-IgG1 |
| | Bisecting.total | gp140.Clade.B | Anti-IgG2 |
| | Sialic.total | gp140.CN54 | Anti-IgG3 |
| | | gp140.Du151 | Anti-IgG4 |
| | | gp140.HXBc2 | Anti-IgG |
| | | gp140.UG21 | |
| | | gp41.HXBc2 | |
| | | gp41.MN | |
| | | HIV1.Gag | |
| | | HIV1.Integrase | |
| | | HIV1.Nef | |
| | | HIV1.p66.6H | |
| | | HIV1.p7 | |
| | | HIV1.Rev | |
| | | HIV1.Vif | |
| | | p24.HXBc2 | |
| | | p24.IIIb | |
| | | p51.HIV1.RT | |
| | | pr55.Gag.IIIb | |
| | | SOSIP.JRFL | |

Fig S1

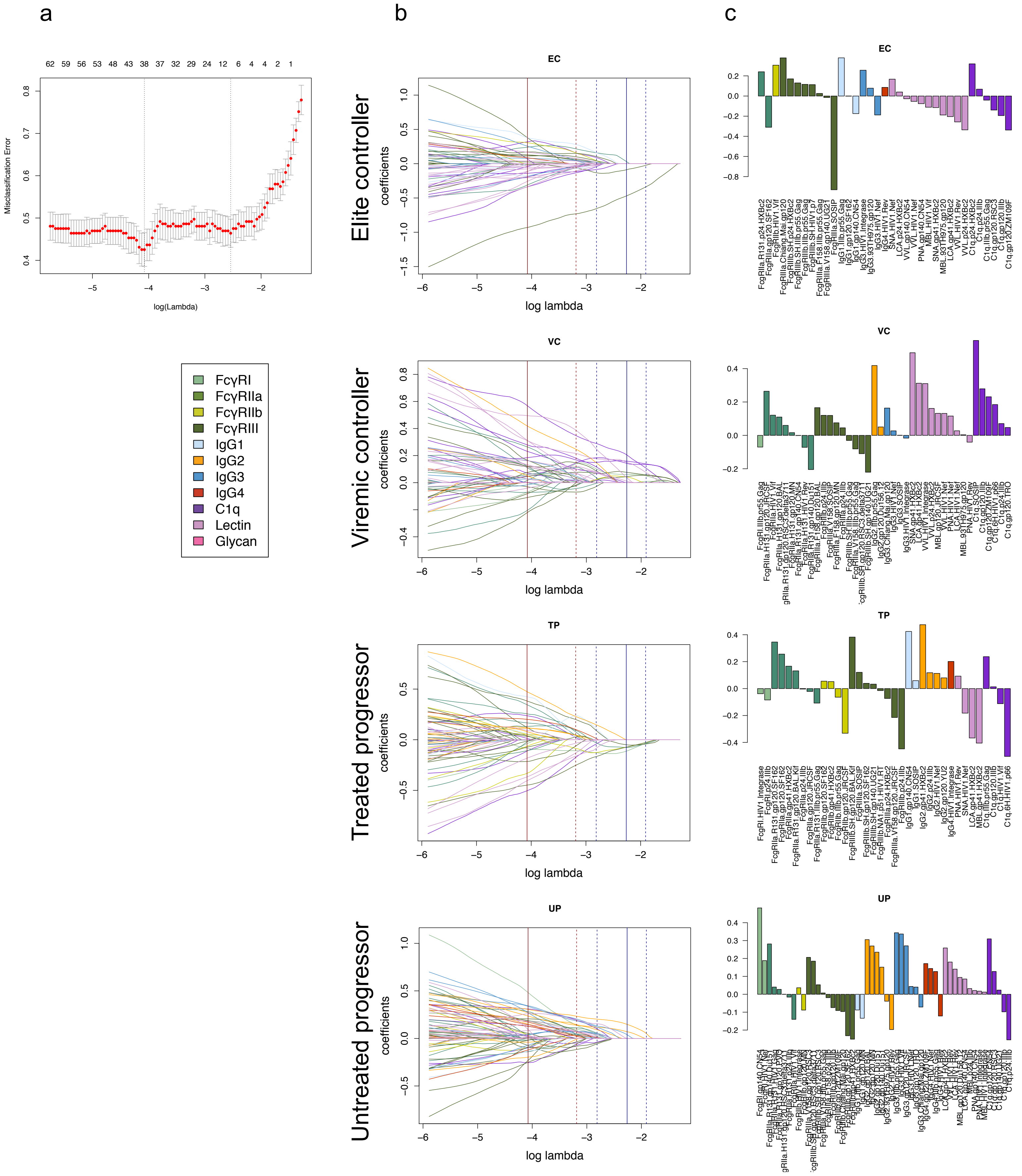

a
b
c
Misclassification Error
log(Lambda)
EC
VC
TP
UP
Elite controller
Viremic controller
Treated progressor
Untreated progressor
coefficients
log lambda
FcγRI
FcγRIIa
FcγRIIb
FcγRIII
IgG1
IgG2
IgG3
IgG4
C1q
Lectin
Glycan

**Figure S1. Classification of subject group using Fc Array features**. **A**) Misclassification error for a representative replicate of glmnet-based multinomial classification discriminating among elite controllers (EC), viremic controllers (VC) and chronic progressors on (TP) or off (UP) antiretroviral therapy. The plot depicts the mean and standard deviation of the misclassification error over 10-fold cross-validation, as the regularization parameter $\lambda$ is varied. The number of Fc Array features resulting for each $\lambda$ is indicated at the top of the plot. Vertical bars mark $\lambda_{min}$ (left) and $\lambda_{1se}$ (right). **B**) Feature coefficient paths, over $\lambda$, for the representative replicate. Vertical lines mark $\lambda_{min}$ (red) and $\lambda_{1se}$ (blue) mean (solid) and standard deviation (dashed) over all 100 replicates. Features are colored by Fc detection reagent. **C**). Feature coefficient magnitudes and identities for the representative model. Features are colored by Fc detection reagent.

Fig S2

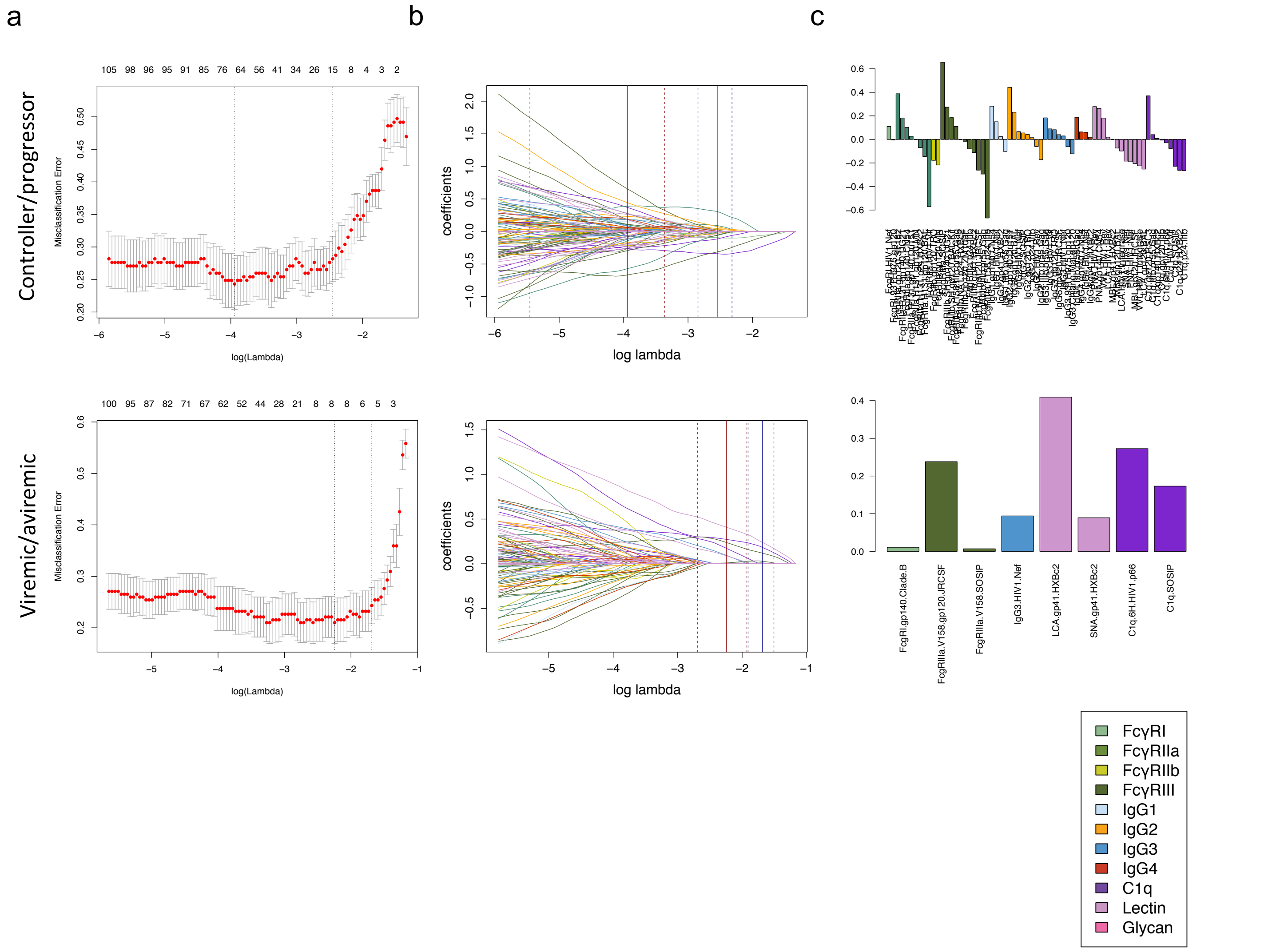


**Figure S2. Classification of progression and viremia status using Fc Array features**. **A**) Misclassification error for a representative replicate of glmnet-based binomial classification discriminating between controller (EC and VC) and progressor (TP and UP) subjects (top row) or between viremic (VC and UP) and aviremic (EC and TP) subjects (bottom row) over different values of the regularization penalty, λ. The plot depicts the mean and standard deviation of the misclassification error over 10-fold cross-validation, as the regularization parameter λ is varied. The number of Fc Array features resulting for each λ is indicated at the top of the plot. Vertical bars mark $\lambda_{min}$ (left) and $\lambda_{1se}$ (right). **B**) Feature coefficient paths, over λ, for the representative replicate. Vertical lines mark $\lambda_{min}$ (red) and $\lambda_{1se}$ (blue) mean (solid) and standard deviation (dashed) over all 100 replicates. Features are colored by Fc detection reagent. **C**). Feature coefficient magnitudes and identities for the representative model. Features are colored by Fc detection reagent.

Fig S3

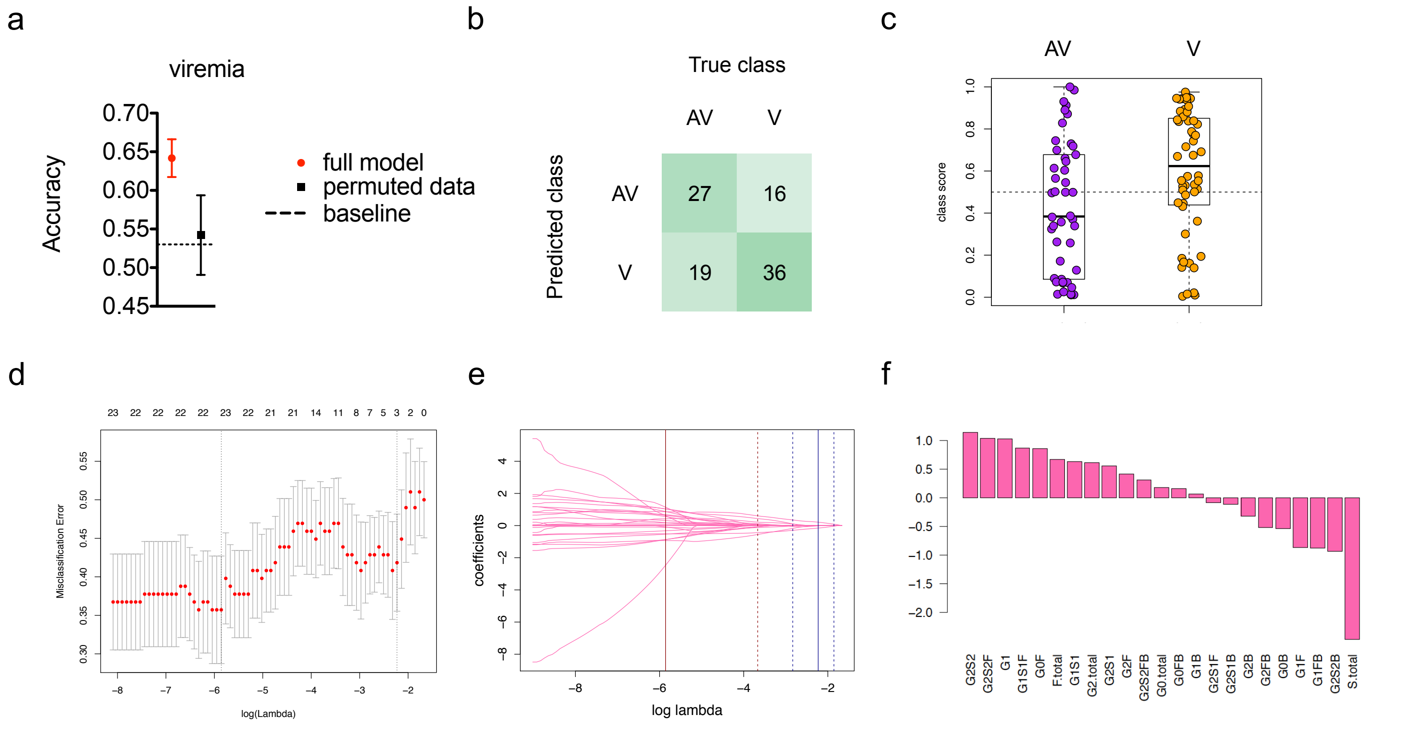


**Figure S3. Classification of viremia status using gp120-specific IgG glycosylation features**. **A**) Accuracy of classification models trained using gp120-specific IgG glycosylation profiles to distinguish between subjects with variable viremia [viremics (V) defined as UP and VC versus aviremics (AV) defined as EC and TP]. Accuracies observed in models (red) are compared to the baseline expectation based on random chance (dotted line) or when classification models were constructed using permuted class labels (black). **B**) Confusion matrix and **C**) and LOD scores for prediction of viremia. **D**) Mean-squared prediction error for a representative replicate of glmnet-based regression predicting different effector functions (each row of panels) from Fc Array features. The plot depicts the mean and standard deviation of the prediction error over 10-fold cross-validation, as the regularization parameter λ is varied. The number of Fc Array features resulting for each λ is indicated at the top of the plot. Vertical bars mark $\lambda_{min}$ (left) and $\lambda_{1se}$ (right). **E**) Feature coefficient paths, over λ, for the representative replicate. Vertical lines mark $\lambda_{min}$ (red) and $\lambda_{1se}$ (blue) mean (solid) and standard deviation (dashed) over all 100 replicates. **F**). Feature coefficient magnitudes and identities for the representative model.

Fig S4

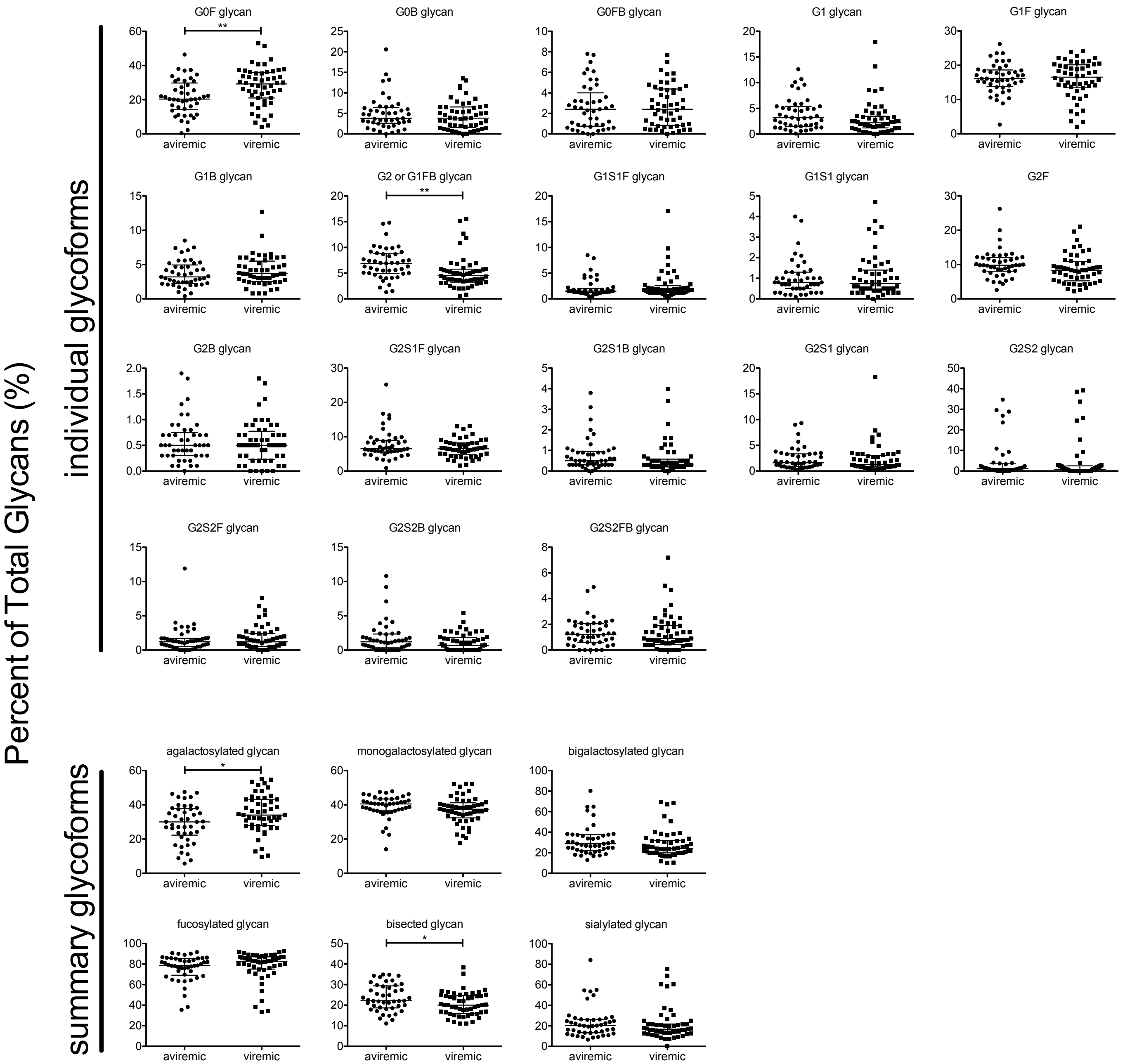


**Figure S4. Levels of IgG glycoforms among gp120-specific antibodies in aviremic (AV) and viremic (V) subjects.** Median and interquartile ranges are plotted. Significance as defined by an uncorrected two-tailed t test is indicated as *p<0.05, **p<0.005.

Fig S5

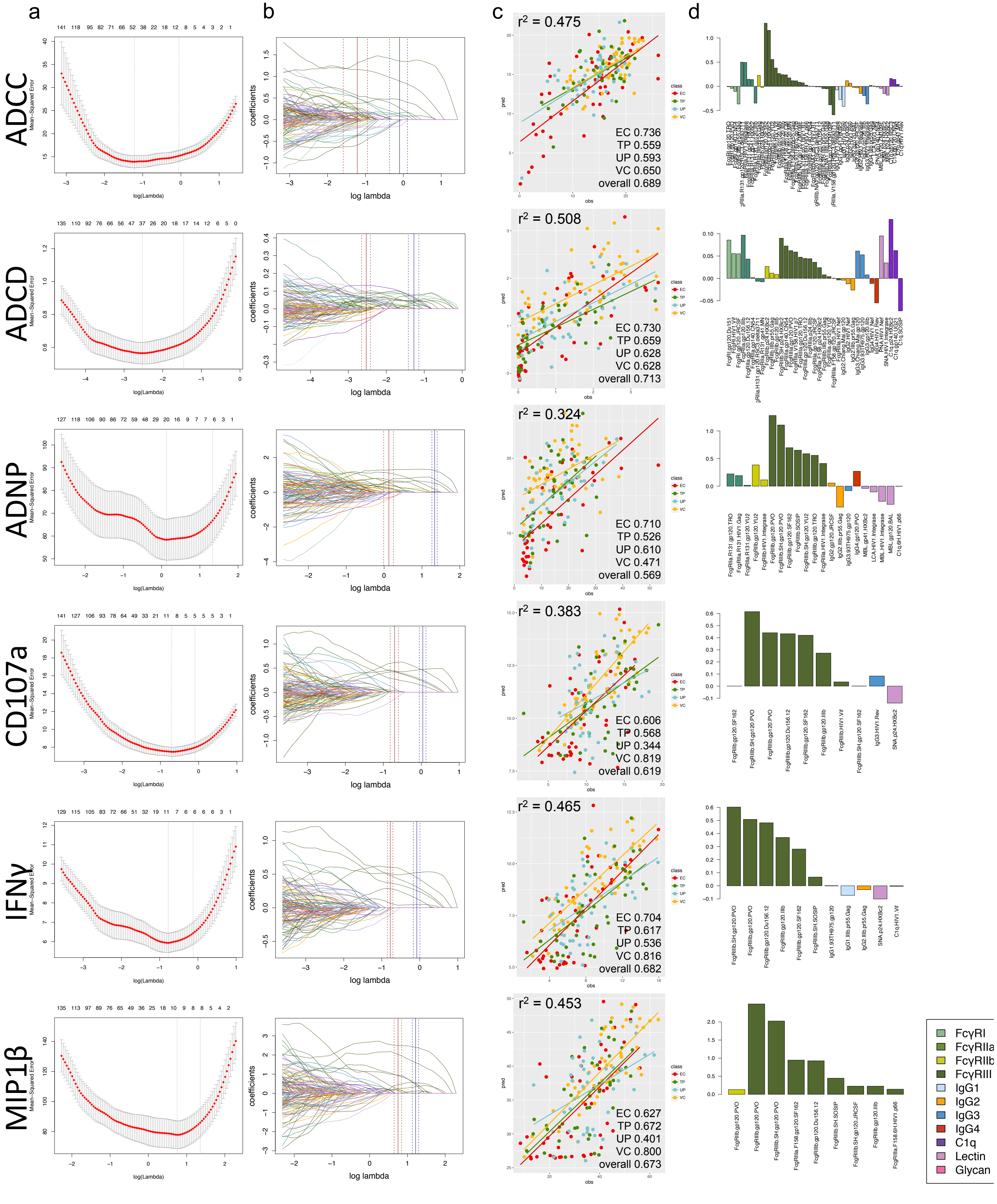

a
b
c
d
ADCC
ADCD
ADNP
CD107a
IFNγ
MIP1β
Mean-Squared Error
log(Lambda)
coefficients
log lambda
pred
obs
class
EC
TP
UP
VC
r² = 0.475
EC 0.736
TP 0.559
UP 0.593
VC 0.650
overall 0.689
r² = 0.508
EC 0.730
TP 0.659
UP 0.628
VC 0.628
overall 0.713
r² = 0.324
EC 0.710
TP 0.526
UP 0.610
VC 0.471
overall 0.569
r² = 0.383
EC 0.606
TP 0.568
UP 0.344
VC 0.819
overall 0.619
r² = 0.465
EC 0.704
TP 0.617
UP 0.536
VC 0.816
overall 0.682
r² = 0.453
EC 0.627
TP 0.672
UP 0.401
VC 0.800
overall 0.673
FcγRI
FcγRIIa
FcγRIIb
FcγRIII
IgG1
IgG2
IgG3
IgG4
C1q
Lectin
Glycan

**Figure S5. Models of antibody effector function learned from Fc Array features**. **A**) Mean-squared prediction error for a representative replicate of glmnet-based regression predicting different effector functions (each row of panels) from Fc Array features. The plot depicts the mean and standard deviation of the prediction error over 10-fold cross-validation, as the regularization parameter $\lambda$ is varied. The number of Fc Array features resulting for each $\lambda$ is indicated at the top of the plot. Vertical bars mark $\lambda_{min}$ (left) and $\lambda_{1se}$ (right). **B**) Feature coefficient paths, over $\lambda$, for the representative replicate. Vertical lines mark $\lambda_{min}$ (red) and $\lambda_{1se}$ (blue) mean (solid) and standard deviation (dashed) over all 100 replicates. Features are colored by Fc detection reagent. **C**) Scatterplots of observed (x-axis) versus predicted (y-axis) antibody activity across subjects for the representative model. Subjects are colored by group and the best fit line for each group is presented. Pearson correlation coefficients for all subjects and each subject group are presented in the inset at lower right, $r^2$ values are reported inset at the upper left. **D**). Feature coefficient magnitudes and identities for the representative model. Features are colored by Fc detection reagent.

Fig S6

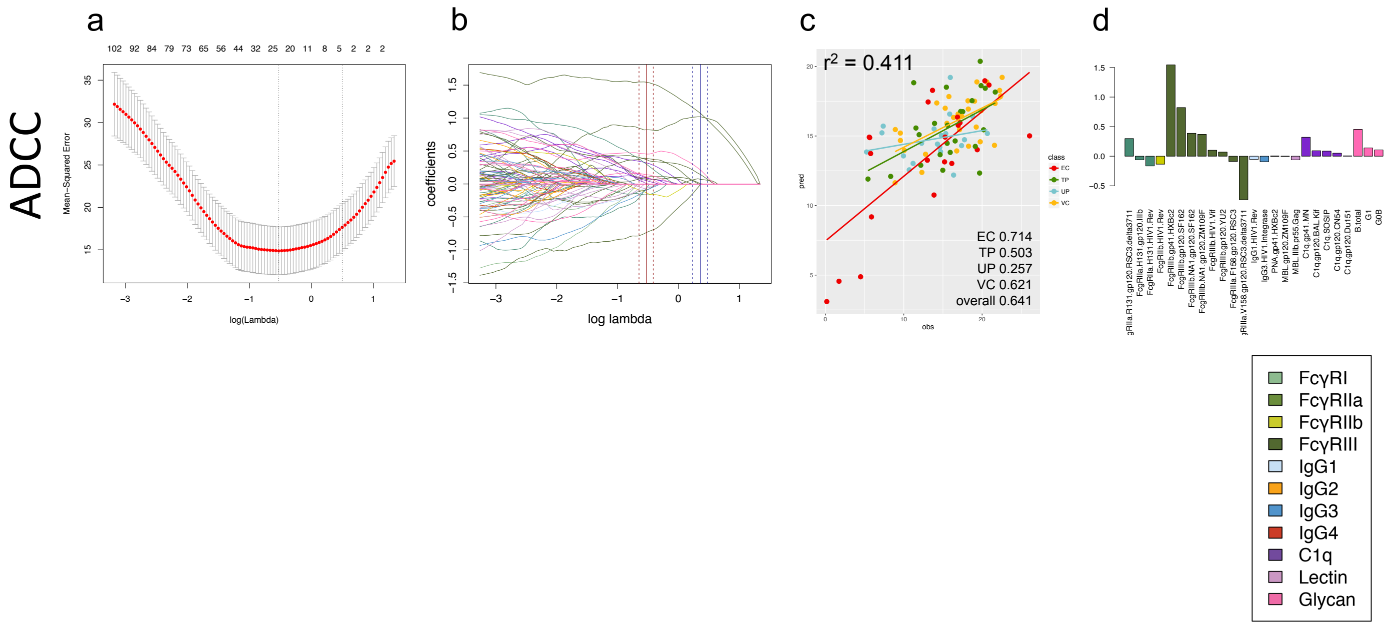


**Figure S6. Models of ADCC learned from Fc Array and gp120-specific IgG glycosylation features**.
**A**) Mean-squared prediction error for a representative replicate of glmnet-based regression predicting ADCC activity from Fc Array and antigen-specific antibody glycosylation features. The plot depicts the mean and standard deviation of the prediction error over 10-fold cross-validation, as the regularization parameter λ is varied. The number of features resulting for each λ is indicated at the top of the plot. Vertical bars mark $\lambda_{min}$ (left) and $\lambda_{1se}$ (right). **B**) Feature coefficient paths, over λ, for the representative replicate. Vertical lines mark $\lambda_{min}$ (red) and $\lambda_{1se}$ (blue) mean (solid) and standard deviation (dashed) over all 100 replicates. Features are colored by Fc detection reagent, and glycoforms are represented in pink. **C**) Scatterplots of observed (x-axis) versus predicted (y-axis) antibody activity across subjects for the representative model. Subjects are colored by group and the best fit line for each group is presented. Pearson correlation coefficients for all subjects and each subject group are presented in the inset at lower right, $r^2$ values are reported inset at the upper left. **D**). Feature coefficient magnitudes and identities for the representative model. Features are colored by Fc detection reagent and glycoforms are represented in pink.

Fig S7

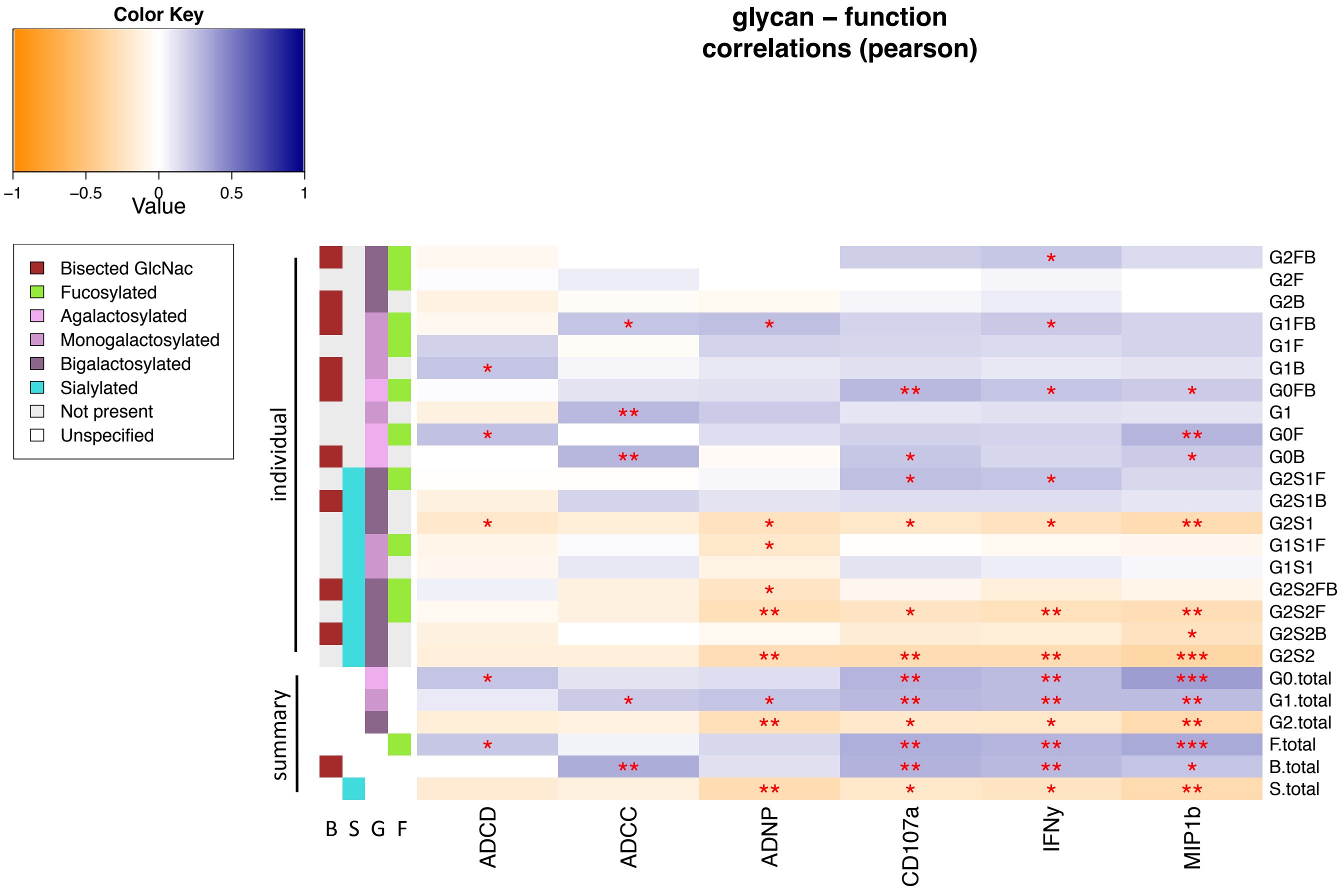


**Figure S7. Correlations observed between gp120-specific IgG glycoforms and antibody effector function across functional assays**. Correlation matrix presenting relationships between gp120-specific antibody glycoform prevalence (y-axis) and antibody effector functions (x-axis). The magnitude and direction of Pearson correlations are shown in orange to blue scale (unadjusted p values reported as *p<0.05; **p<0.01; ***p<0.001). Glycoform identities are graphically presented in the color bar at left, where the level of galactosylation (G0, G1, or G2) is in purple scale, and the presence of bisecting GlcNAc (B), Sialic Acid (S), and Fucose indicated in burgundy, turquoise, and green, respectively.
