# Supplementary material for: High‐resolution definition of humoral immune response correlates of effective immunity against HIV
Source: Mol Syst Biol. 2018 Mar 26;14(3):e7881. doi: 10.15252/msb.20177881 (PMC5868198; doi:10.15252/msb.20177881)
Supplement: Supplementary file 5 — Dataset EV3 [file MSB-14-e7881-s005.zip › dataset_EV3/Fc.array/class.etuv/lambda.min/fullmod-coeffs.pdf]

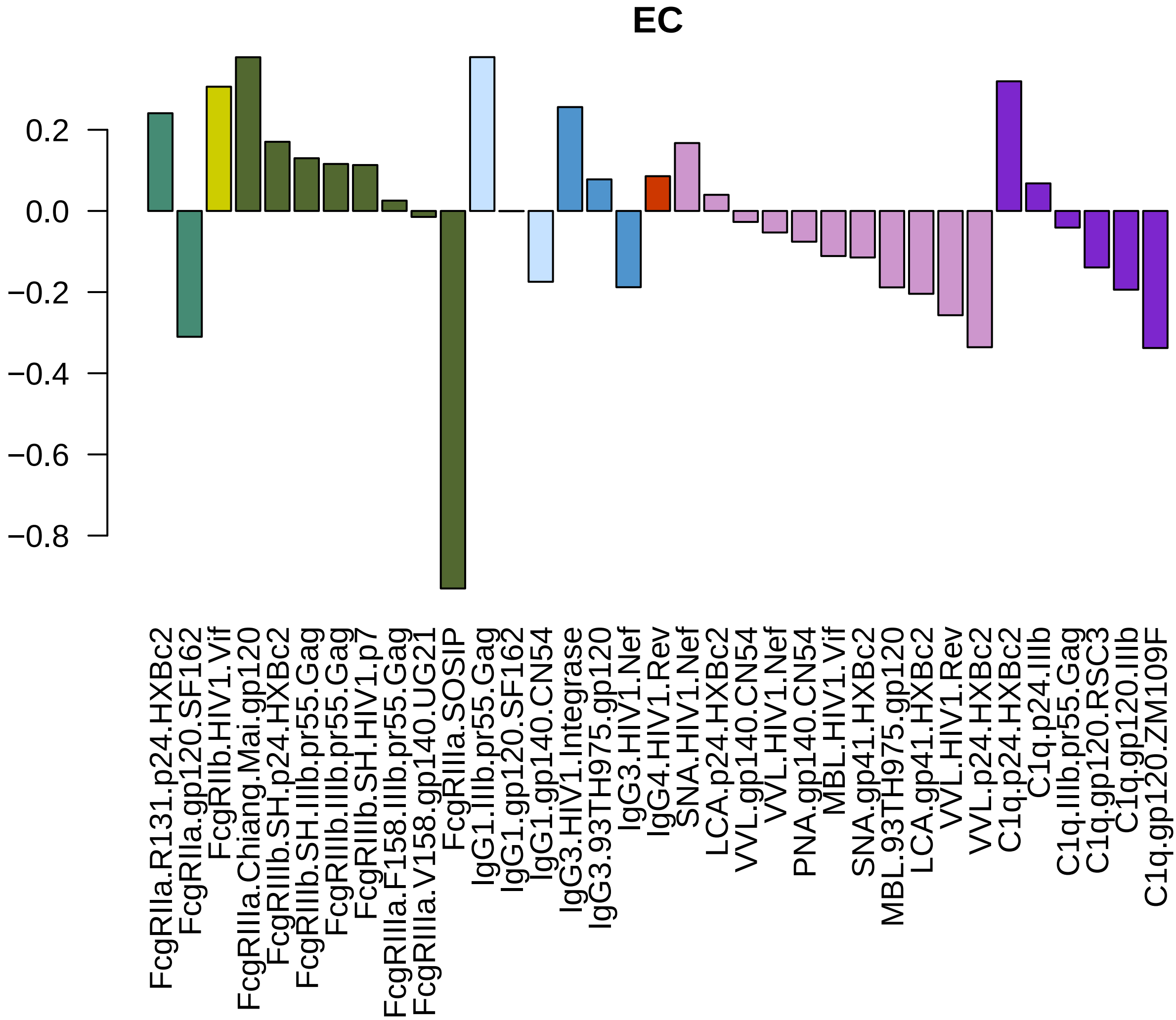

EC
0.2
0.0
−0.2
−0.4
−0.6
−0.8
FcgRIIa.R131.p24.HXBc2
FcgRIIa.gp120.SF162
FcgRIIb.HIV1.Vif
FcgRIIIa.Chiang.Mai.gp120
FcgRIIIb.SH.p24.HXBc2
FcgRIIIb.SH.IIIb.pr55.Gag
FcgRIIIb.IIIb.pr55.Gag
FcgRIIIb.SH.HIV1.p7
FcgRIIIa.F158.IIIb.pr55.Gag
FcgRIIIa.V158.gp140.UG21
FcgRIIIa.SOSIP
IgG1.IIIb.pr55.Gag
IgG1.gp120.SF162
IgG1.gp140.CN54
IgG3.HIV1.Integrase
IgG3.93TH975.gp120
IgG3.HIV1.Nef
IgG4.HIV1.Rev
SNA.HIV1.Nef
LCA.p24.HXBc2
VVL.gp140.CN54
VVL.HIV1.Nef
PNA.gp140.CN54
MBL.HIV1.Vif
SNA.gp41.HXBc2
MBL.93TH975.gp120
LCA.gp41.HXBc2
VVL.HIV1.Rev
VVL.p24.HXBc2
C1q.p24.HXBc2
C1q.p24.IIIb
C1q.IIIb.pr55.Gag
C1q.gp120.RSC3
C1q.gp120.IIIb
C1q.gp120.ZM109F

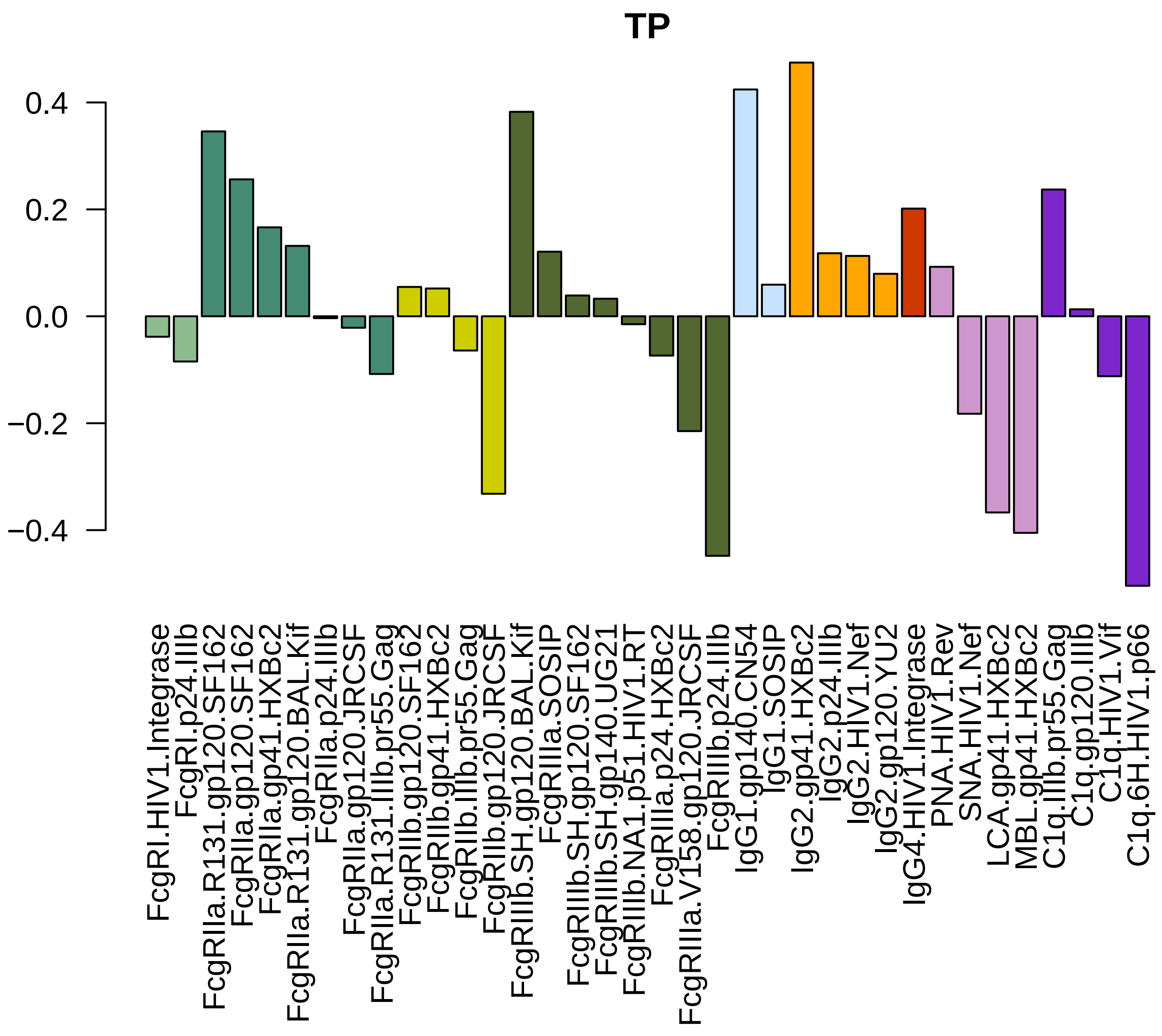

TP
0.4
0.2
0.0
−0.2
−0.4
FcgRI.HIV1.Integrase
FcgRI.p24.IIIb
FcgRIIa.R131.gp120.SF162
FcgRIIa.gp120.SF162
FcgRIIa.gp41.HXBc2
FcgRIIa.R131.gp120.BAL.Kif
FcgRIIa.p24.IIIb
FcgRIIa.gp120.JRCSF
FcgRIIa.R131.IIIb.pr55.Gag
FcgRIIb.gp120.SF162
FcgRIIb.gp41.HXBc2
FcgRIIb.IIIb.pr55.Gag
FcgRIIb.gp120.JRCSF
FcgRIIIb.SH.gp120.BAL.Kif
FcgRIIIa.SOSIP
FcgRIIIb.SH.gp120.SF162
FcgRIIIb.SH.gp140.UG21
FcgRIIIb.NA1.p51.HIV1.RT
FcgRIIIa.p24.HXBc2
FcgRIIIa.V158.gp120.JRCSF
FcgRIIIb.p24.IIIb
IgG1.gp140.CN54
IgG1.SOSIP
IgG2.gp41.HXBc2
IgG2.p24.IIIb
IgG2.HIV1.Nef
IgG2.gp120.YU2
IgG4.HIV1.Integrase
PNA.HIV1.Rev
SNA.HIV1.Nef
LCA.gp41.HXBc2
MBL.gp41.HXBc2
C1q.IIIb.pr55.Gag
C1q.gp120.IIIb
C1q.HIV1.Vif
C1q.6H.HIV1.p66

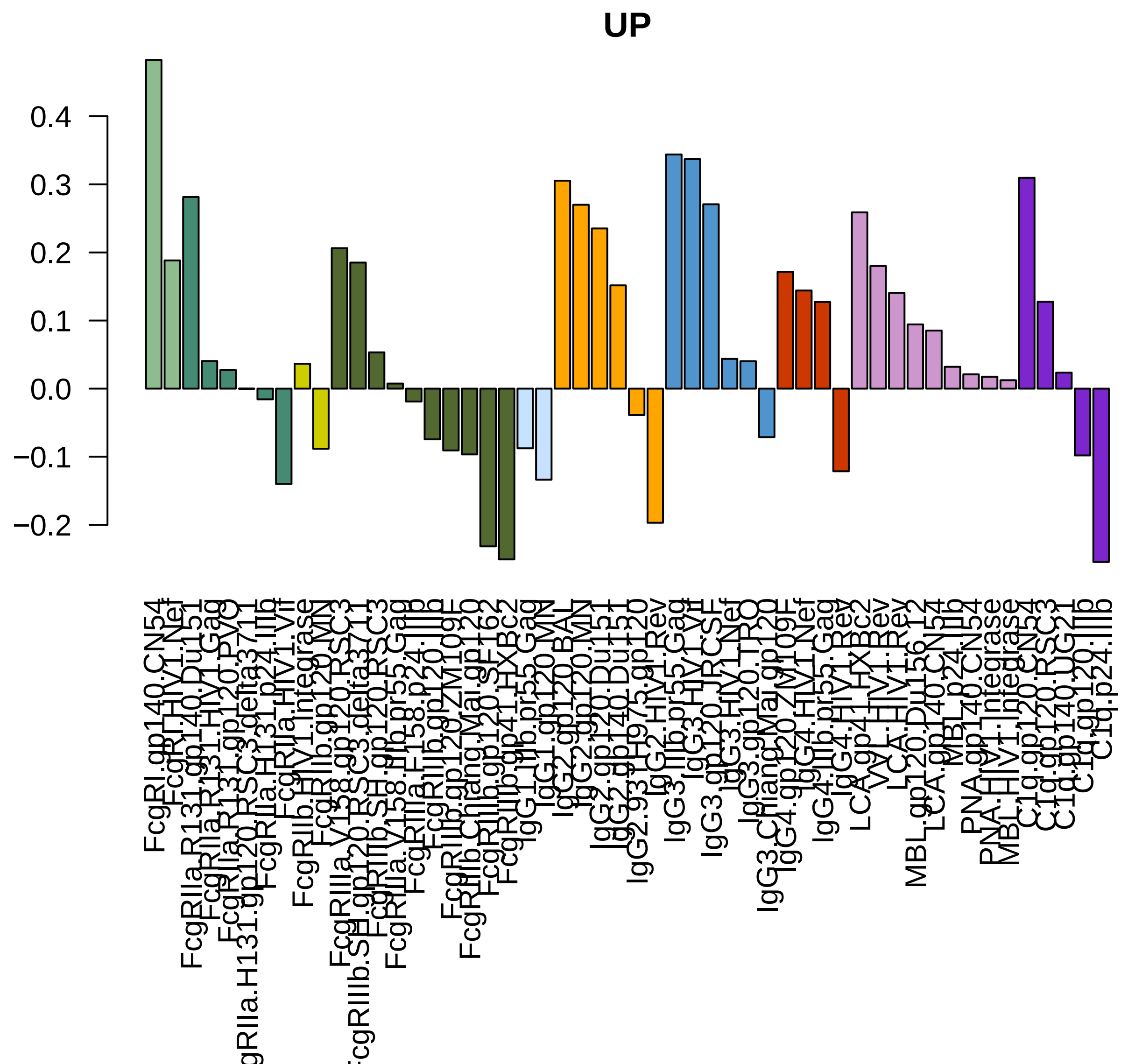

UP
0.4
0.3
0.2
0.1
0.0
−0.1
−0.2

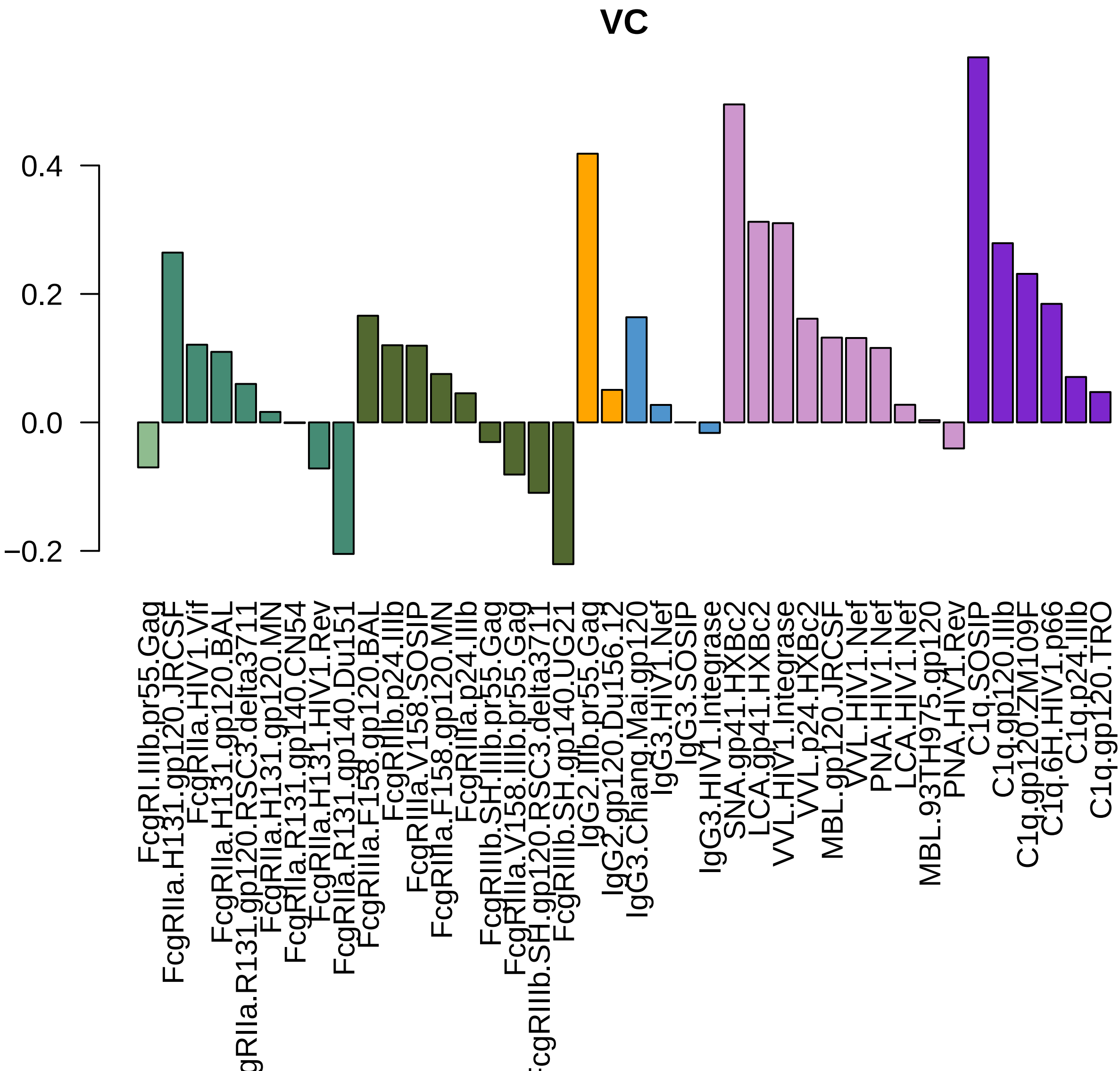

VC
0.4
0.2
0.0
−0.2
FcgRI.IIIb.pr55.Gag
FcgRIIa.H131.gp120.JRCSF
FcgRIIa.HIV1.Vif
FcgRIIa.H131.gp120.BAL
gRIIa.R131.gp120.RSC3.delta3711
FcgRIIa.H131.gp120.MN
FcgRIIa.R131.gp140.CN54
FcgRIIa.H131.HIV1.Rev
FcgRIIa.R131.gp140.Du151
FcgRIIIa.F158.gp120.BAL
FcgRIIIb.p24.IIIb
FcgRIIIa.V158.SOSIP
FcgRIIIa.F158.gp120.MN
FcgRIIIa.p24.IIIb
FcgRIIIb.SH.IIIb.pr55.Gag
FcgRIIIa.V158.IIIb.pr55.Gag
cgRIIIb.SH.gp120.RSC3.delta3711
FcgRIIIb.SH.gp140.UG21
IgG2.IIIb.pr55.Gag
IgG2.gp120.Du156.12
IgG3.Chiang.Mai.gp120
IgG3.HIV1.Nef
IgG3.SOSIP
IgG3.HIV1.Integrase
SNA.gp41.HXBc2
LCA.gp41.HXBc2
VVL.HIV1.Integrase
VVL.p24.HXBc2
MBL.gp120.JRCSF
VVL.HIV1.Nef
PNA.HIV1.Nef
LCA.HIV1.Nef
MBL.93TH975.gp120
PNA.HIV1.Rev
C1q.SOSIP
C1q.gp120.IIIb
C1q.gp120.ZM109F
C1q.6H.HIV1.p66
C1q.p24.IIIb
C1q.gp120.TRO
