# Supplementary figures and images for: High‐resolution definition of humoral immune response correlates of effective immunity against HIV
Source: Mol Syst Biol. 2018 Mar 26;14(3):e7881. doi: 10.15252/msb.20177881 (PMC5868198; doi:10.15252/msb.20177881)

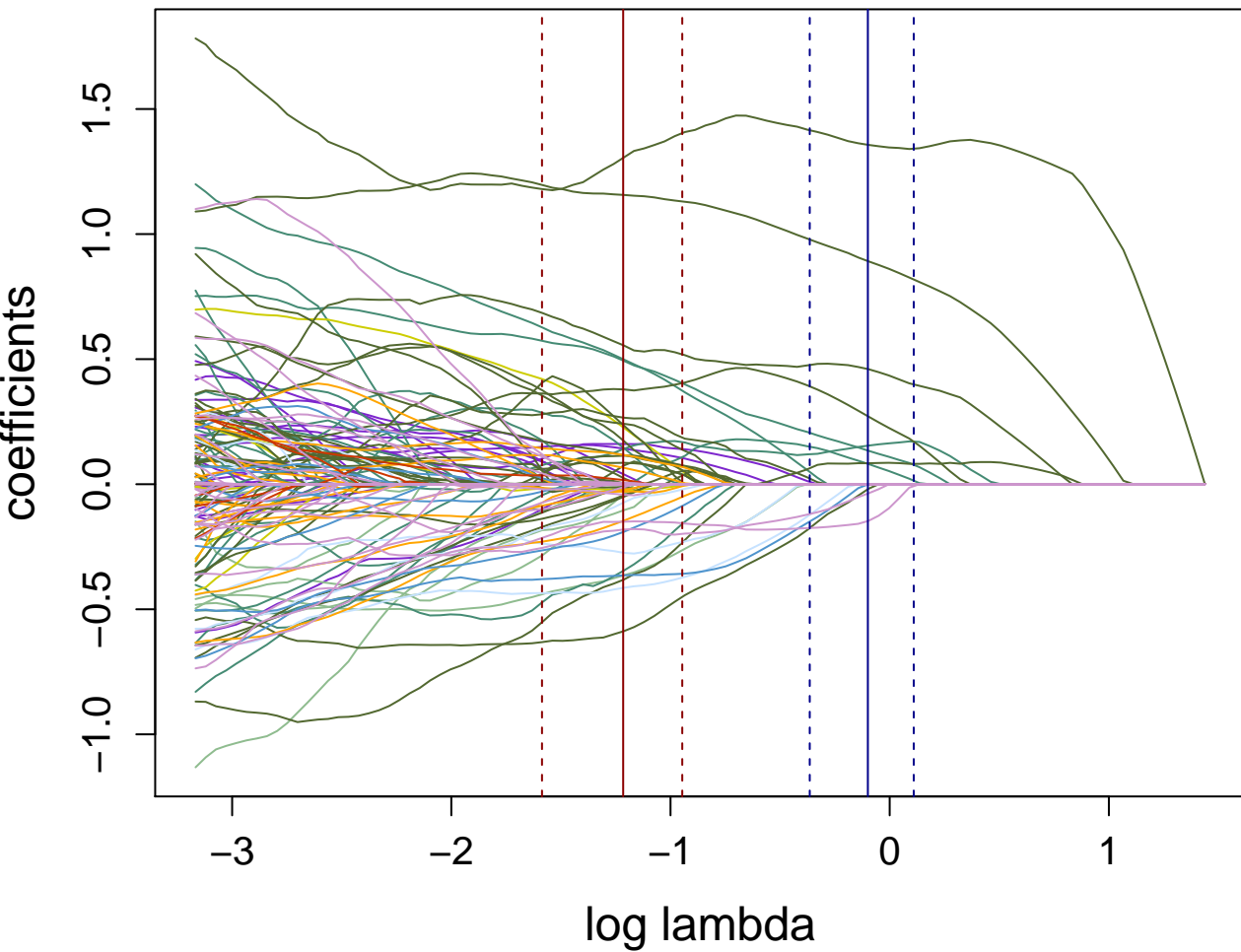

Supplement: Supplementary file 5 — Dataset EV3 [file MSB-14-e7881-s005.zip › dataset_EV3/Fc.array/ADCC/coeff-path.pdf]

Mean-Squared Error

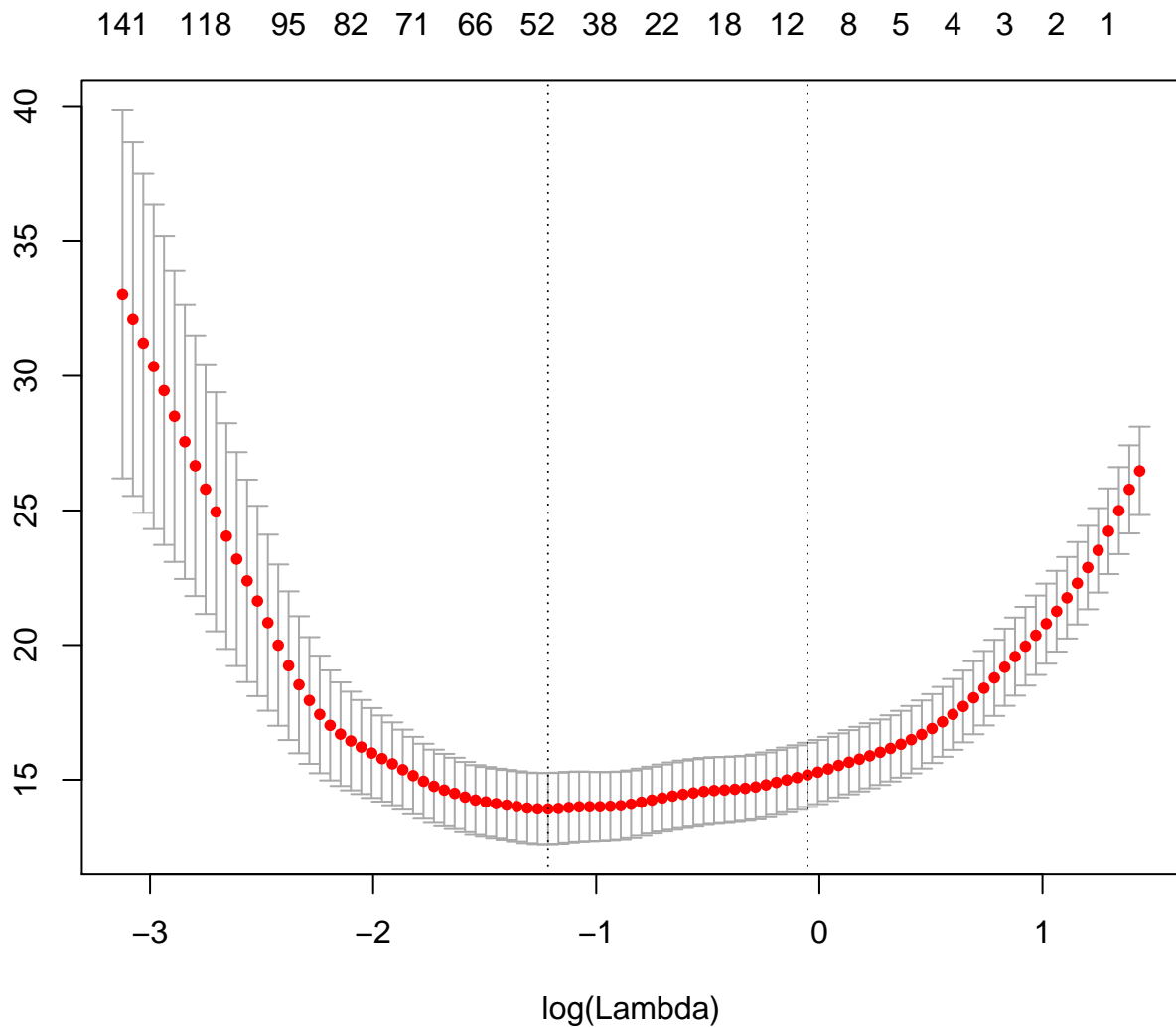

Supplement: Supplementary file 5 — Dataset EV3 [file MSB-14-e7881-s005.zip › dataset_EV3/Fc.array/ADCC/lambda.min/cv-results.pdf]

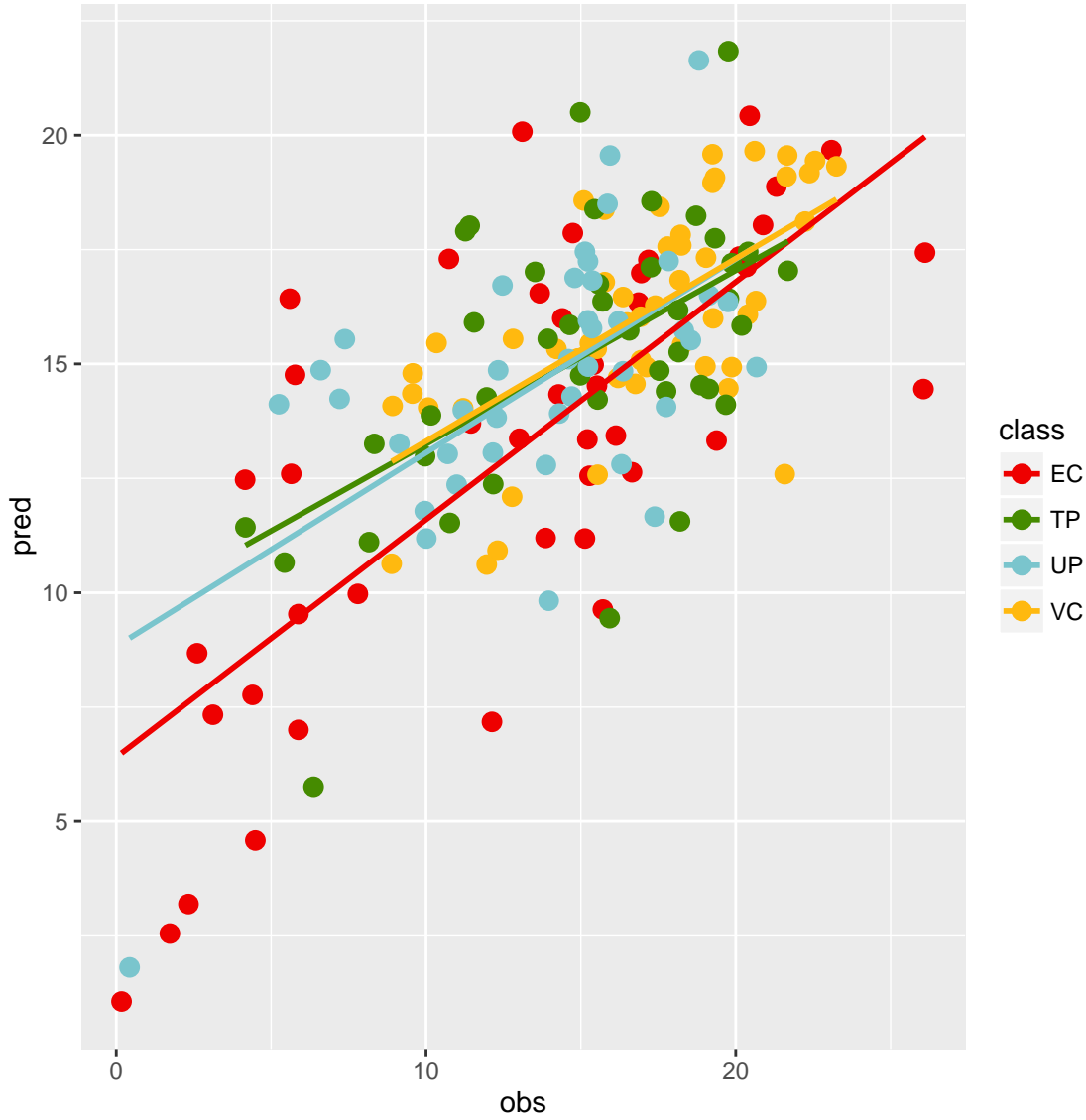

Supplement: Supplementary file 5 — Dataset EV3 [file MSB-14-e7881-s005.zip › dataset_EV3/Fc.array/ADCC/lambda.min/cvmod-scatter.pdf]

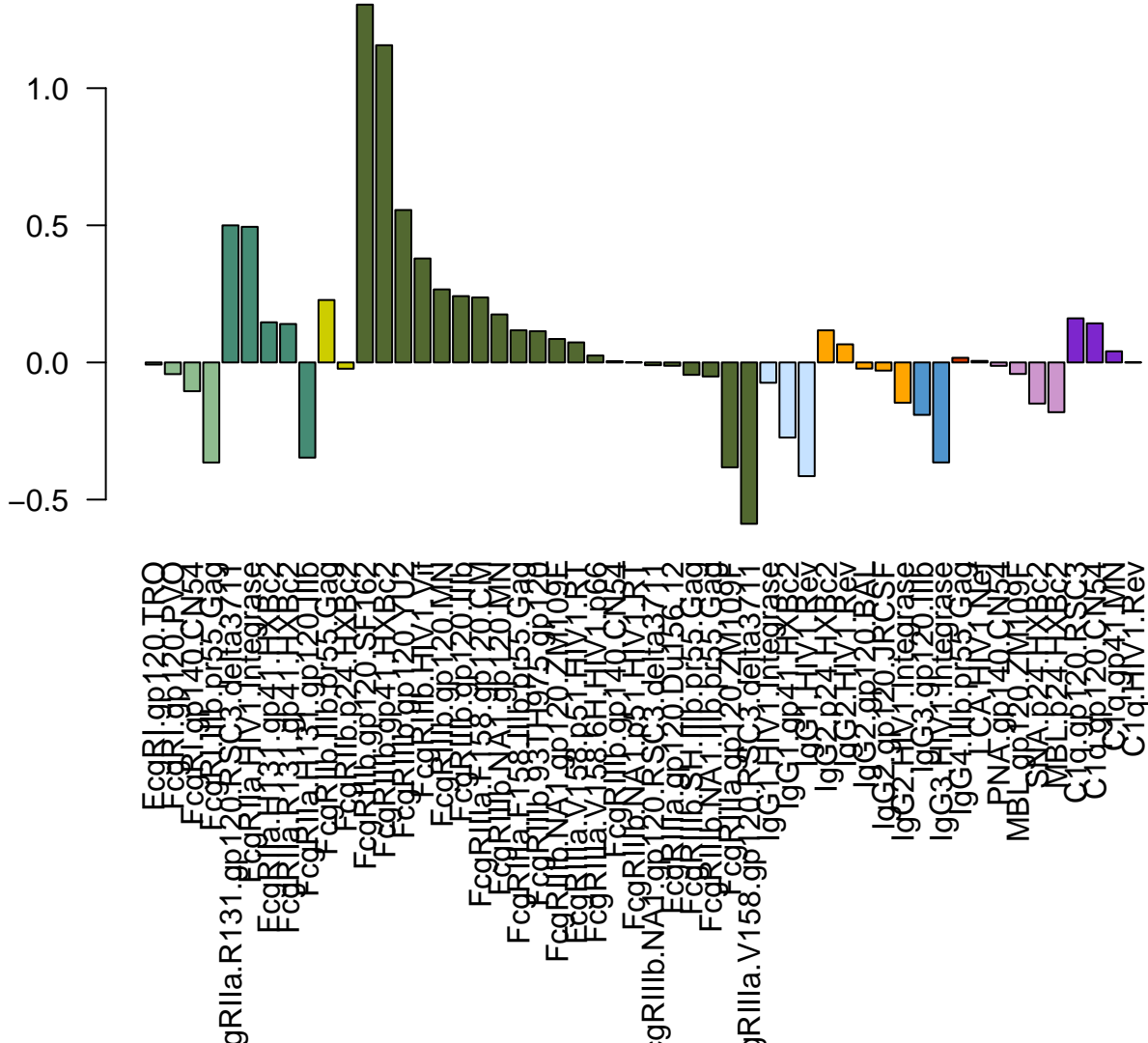

Supplement: Supplementary file 5 — Dataset EV3 [file MSB-14-e7881-s005.zip › dataset_EV3/Fc.array/ADCC/lambda.min/fullmod-coeffs.pdf]

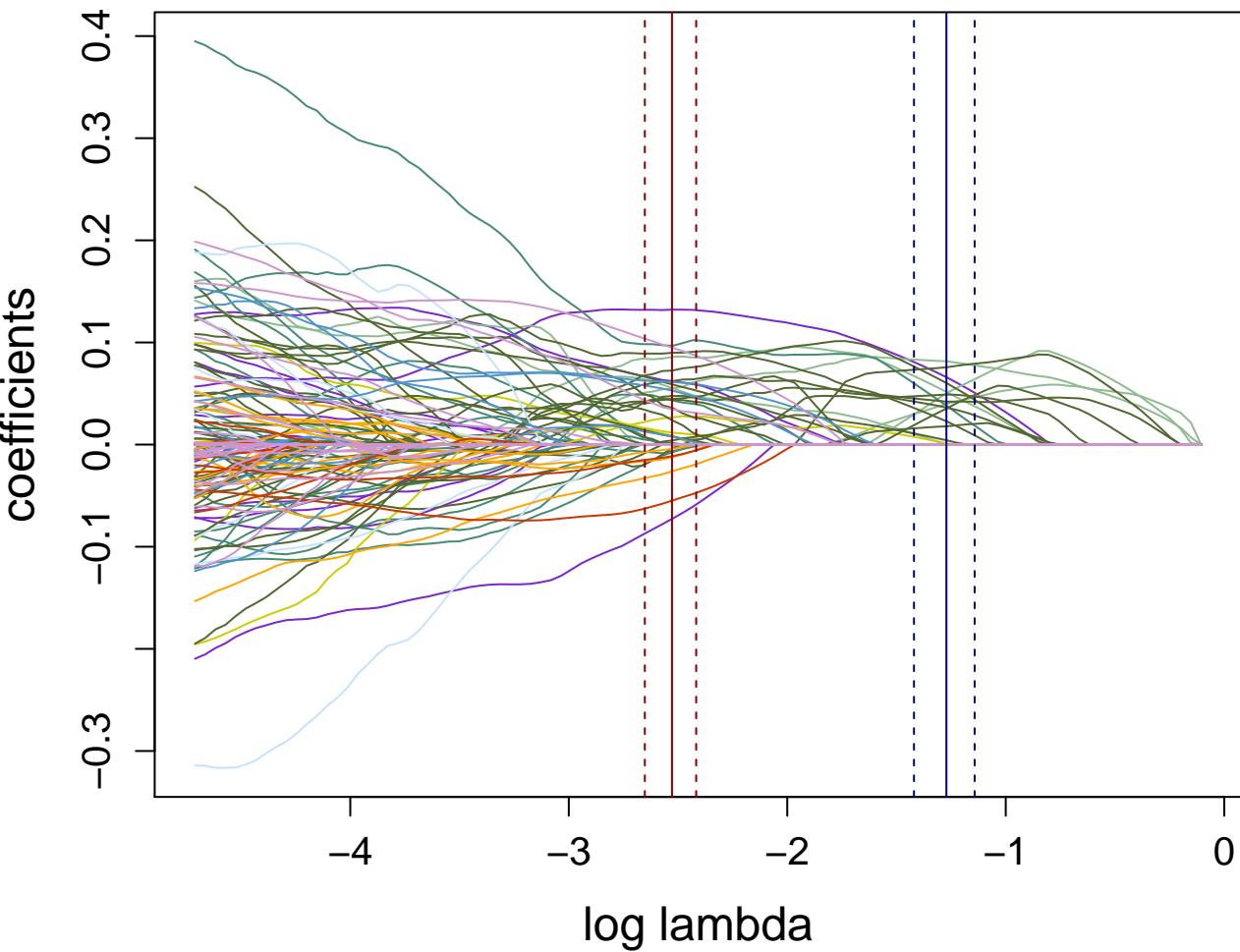

Supplement: Supplementary file 5 — Dataset EV3 [file MSB-14-e7881-s005.zip › dataset_EV3/Fc.array/ADCD/coeff-path.pdf]

Mean-Squared Error

135 110 92 76 66 56 47 37 26 20 18 17 14 12 6 5 0

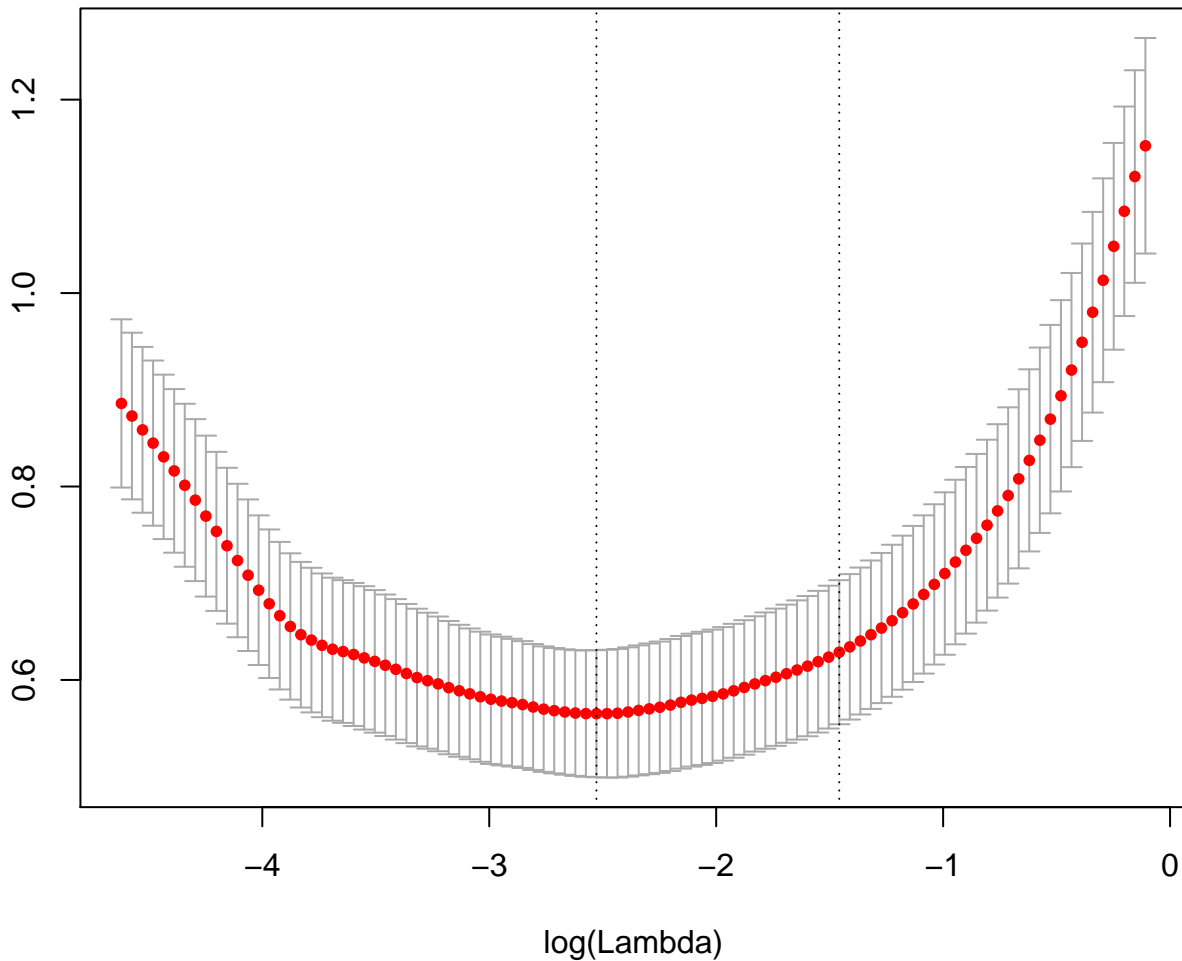

Supplement: Supplementary file 5 — Dataset EV3 [file MSB-14-e7881-s005.zip › dataset_EV3/Fc.array/ADCD/lambda.min/cv-results.pdf]

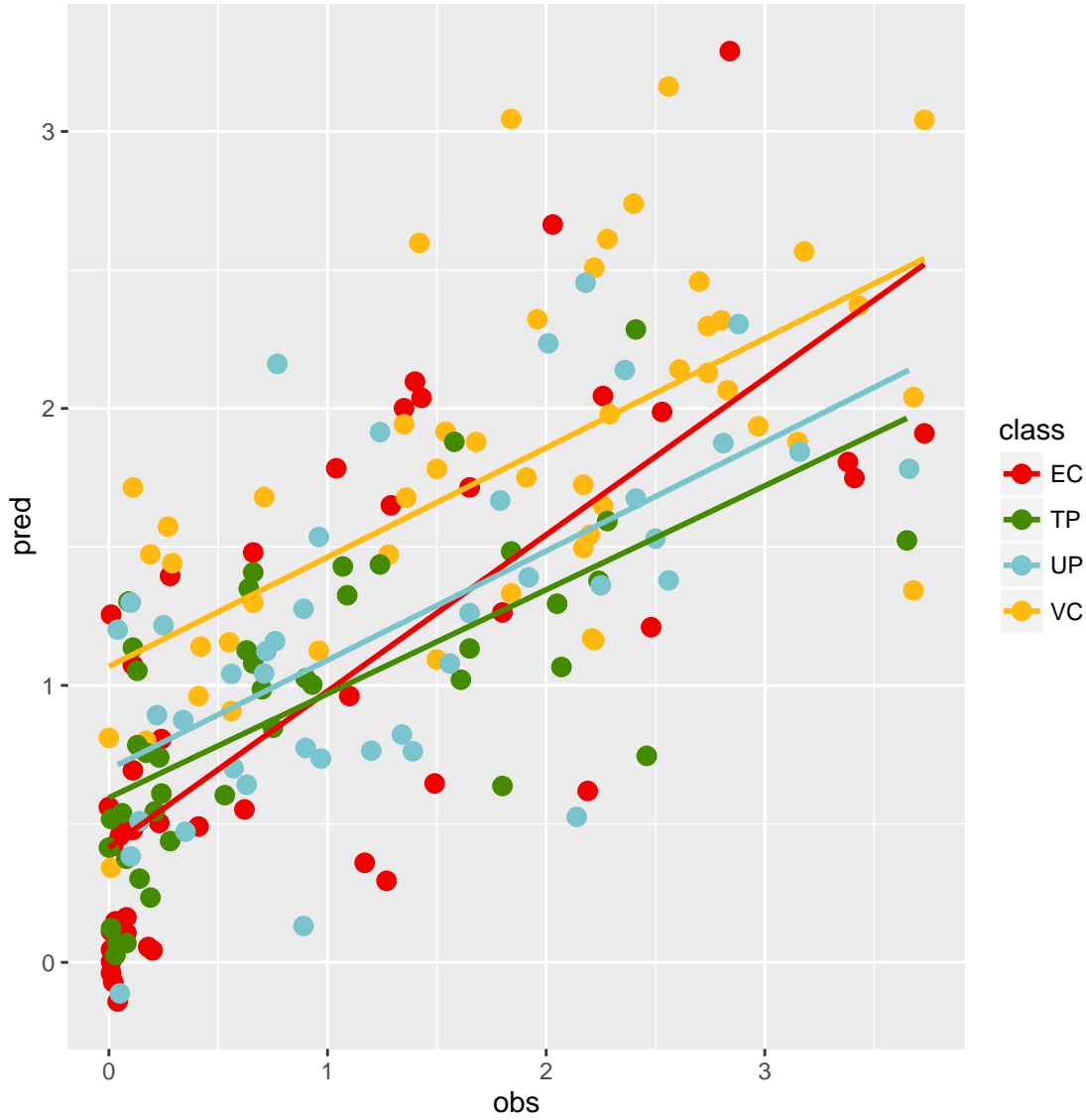

Supplement: Supplementary file 5 — Dataset EV3 [file MSB-14-e7881-s005.zip › dataset_EV3/Fc.array/ADCD/lambda.min/cvmod-scatter.pdf]

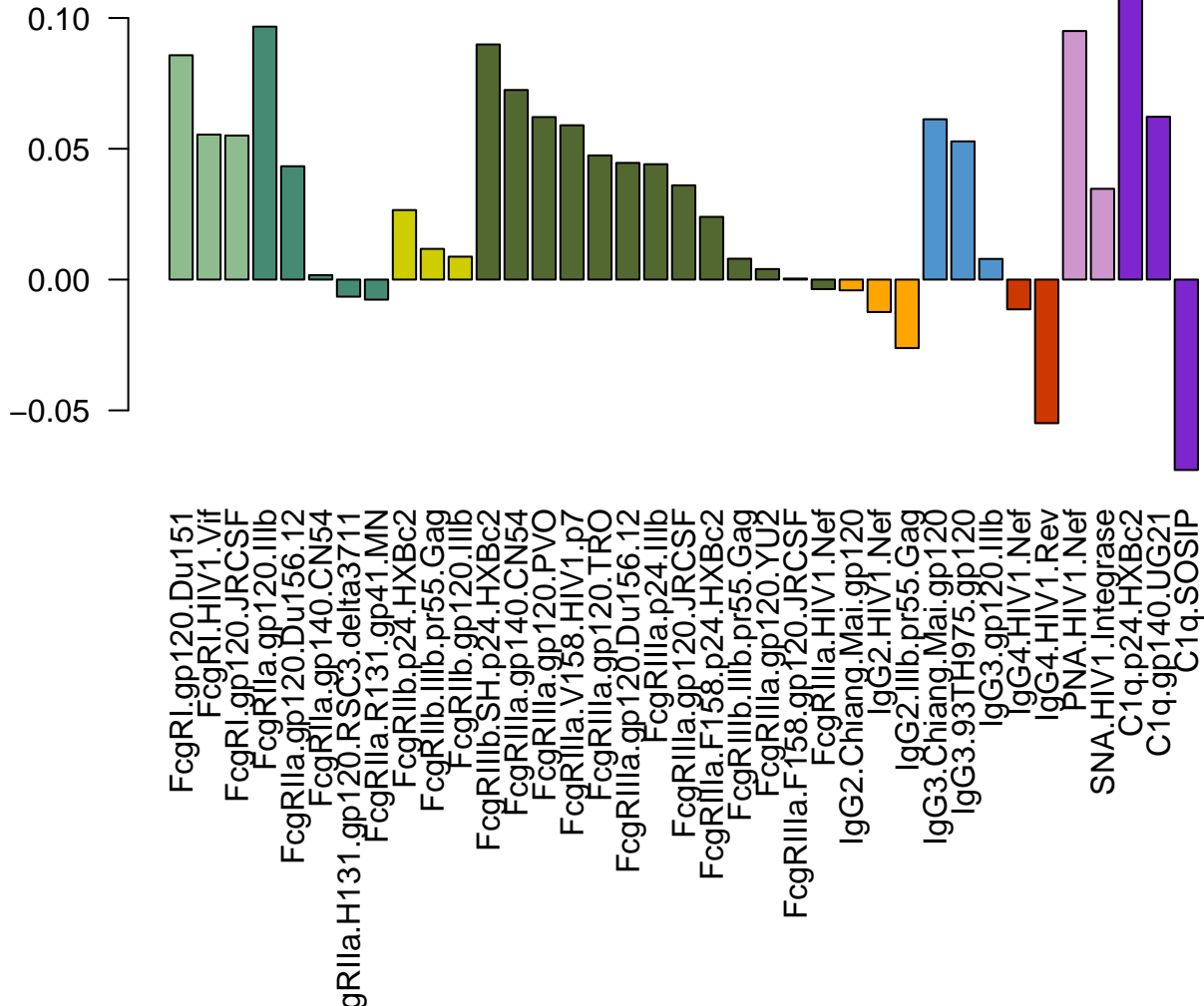

Supplement: Supplementary file 5 — Dataset EV3 [file MSB-14-e7881-s005.zip › dataset_EV3/Fc.array/ADCD/lambda.min/fullmod-coeffs.pdf]

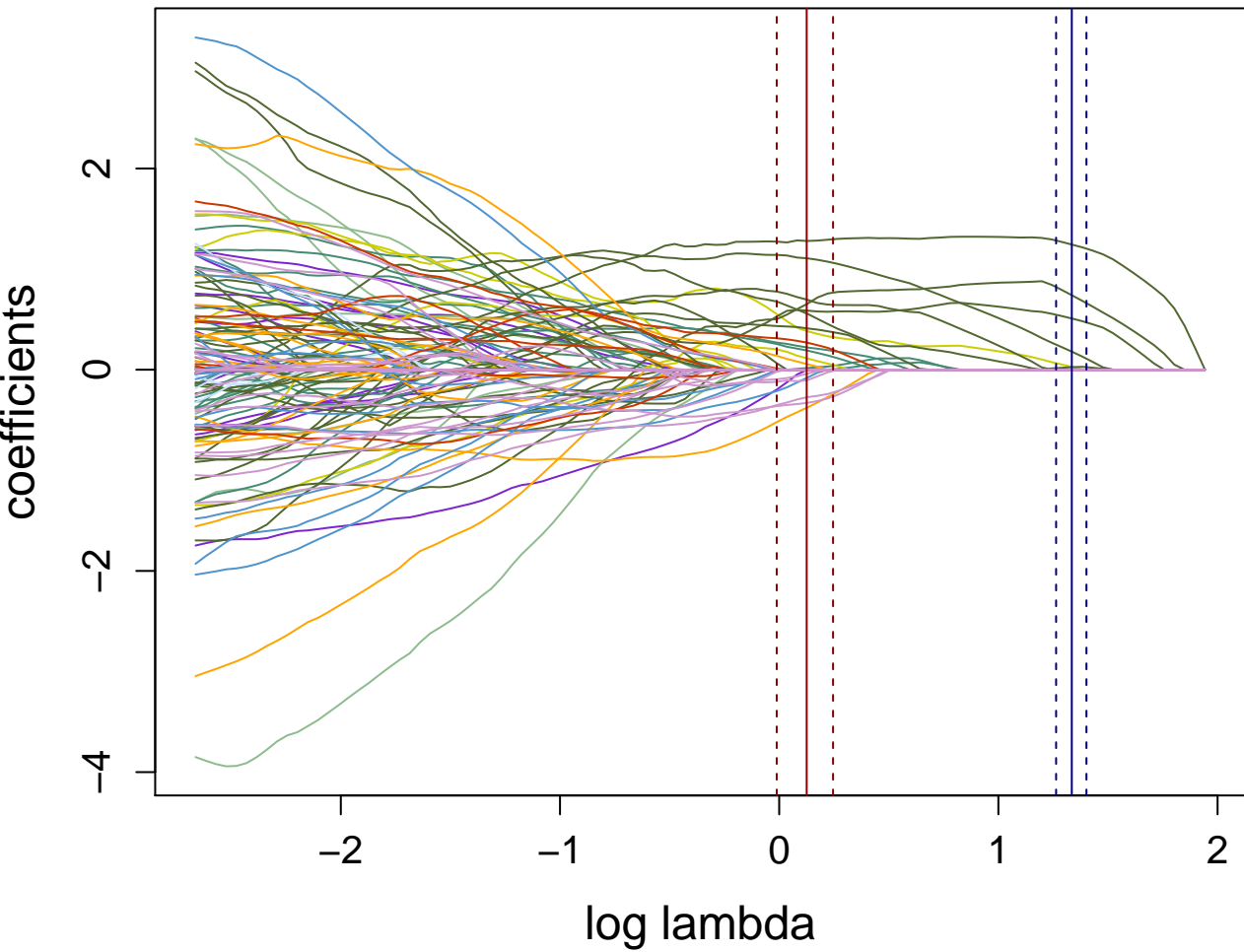

Supplement: Supplementary file 5 — Dataset EV3 [file MSB-14-e7881-s005.zip › dataset_EV3/Fc.array/ADNP/coeff-path.pdf]

Mean-Squared Error

127 118 106 90 86 72 59 48 29 20 16 9 7 7 6 3 1

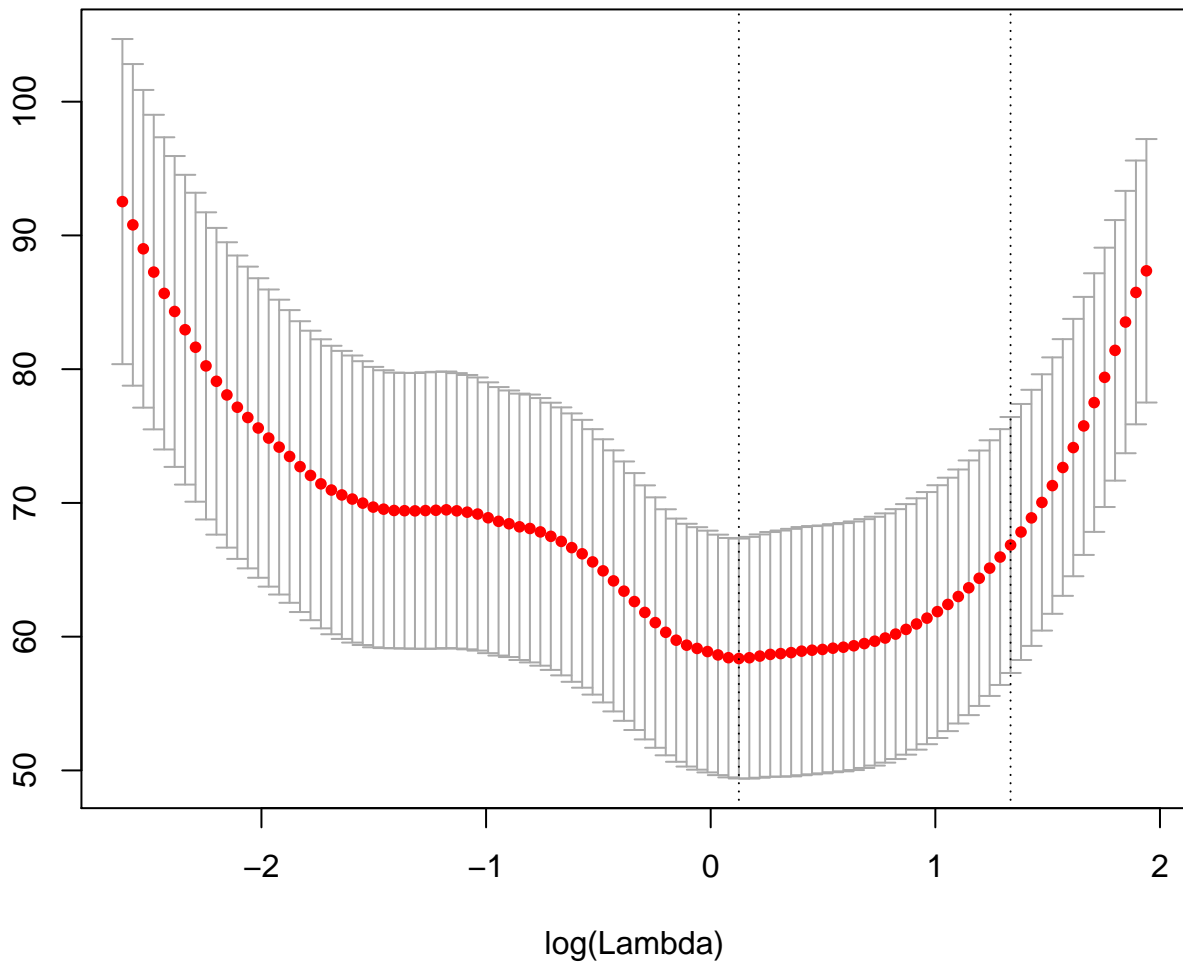

Supplement: Supplementary file 5 — Dataset EV3 [file MSB-14-e7881-s005.zip › dataset_EV3/Fc.array/ADNP/lambda.min/cv-results.pdf]

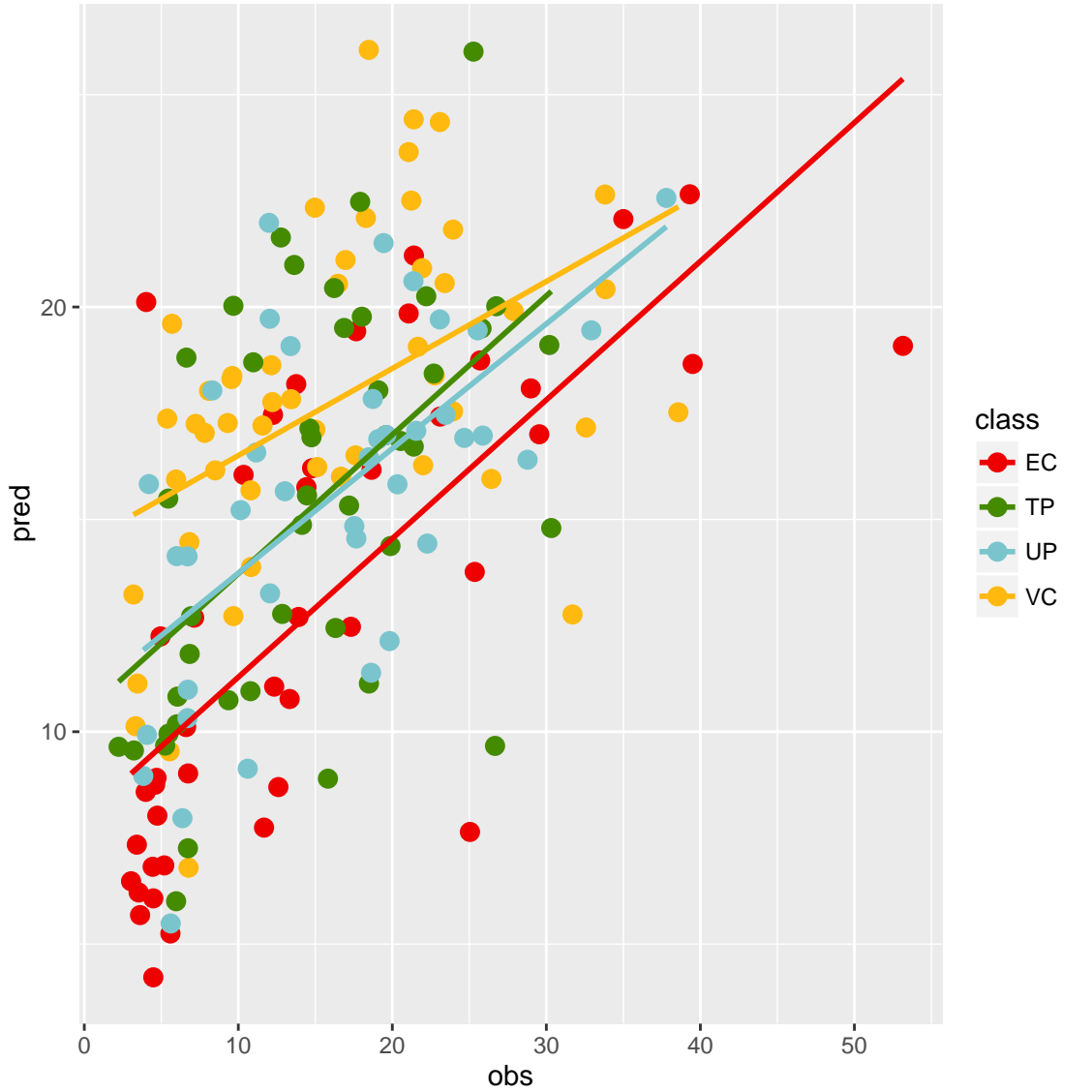

Supplement: Supplementary file 5 — Dataset EV3 [file MSB-14-e7881-s005.zip › dataset_EV3/Fc.array/ADNP/lambda.min/cvmod-scatter.pdf]

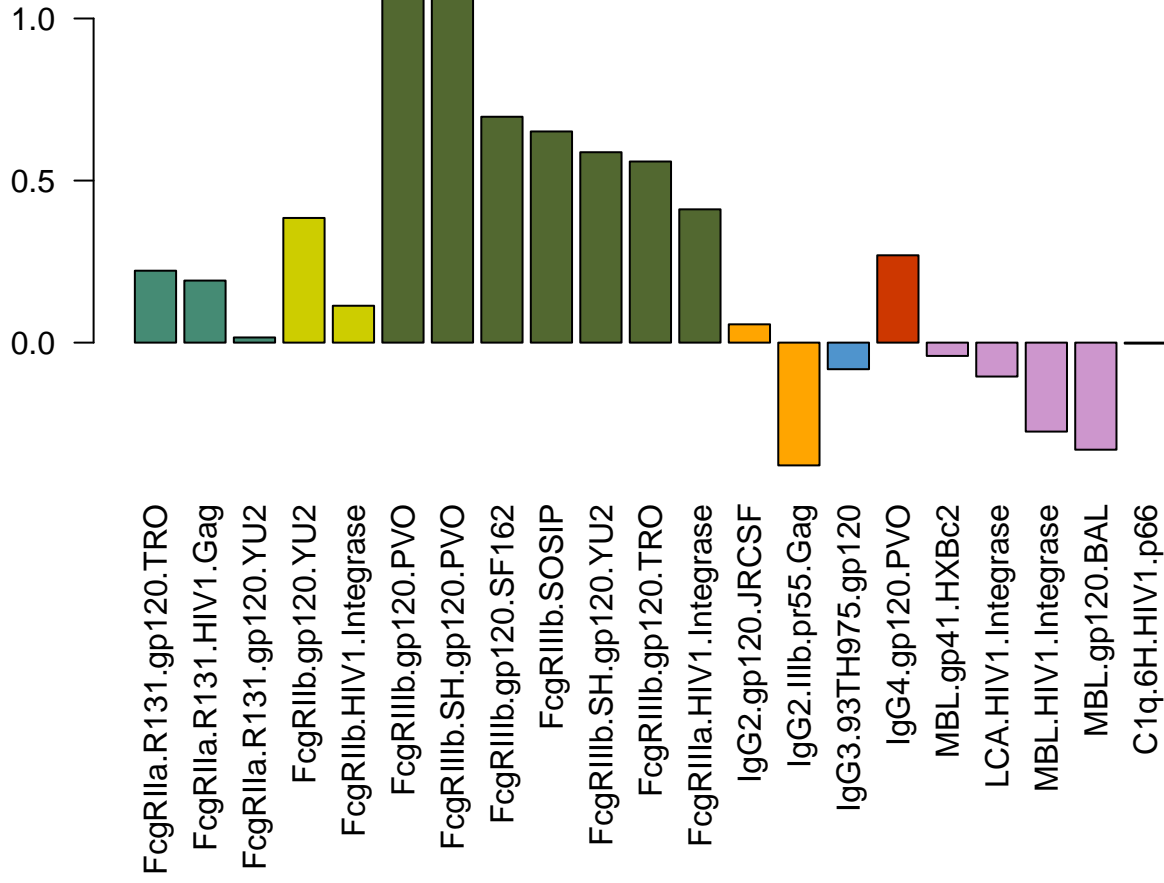

Supplement: Supplementary file 5 — Dataset EV3 [file MSB-14-e7881-s005.zip › dataset_EV3/Fc.array/ADNP/lambda.min/fullmod-coeffs.pdf]

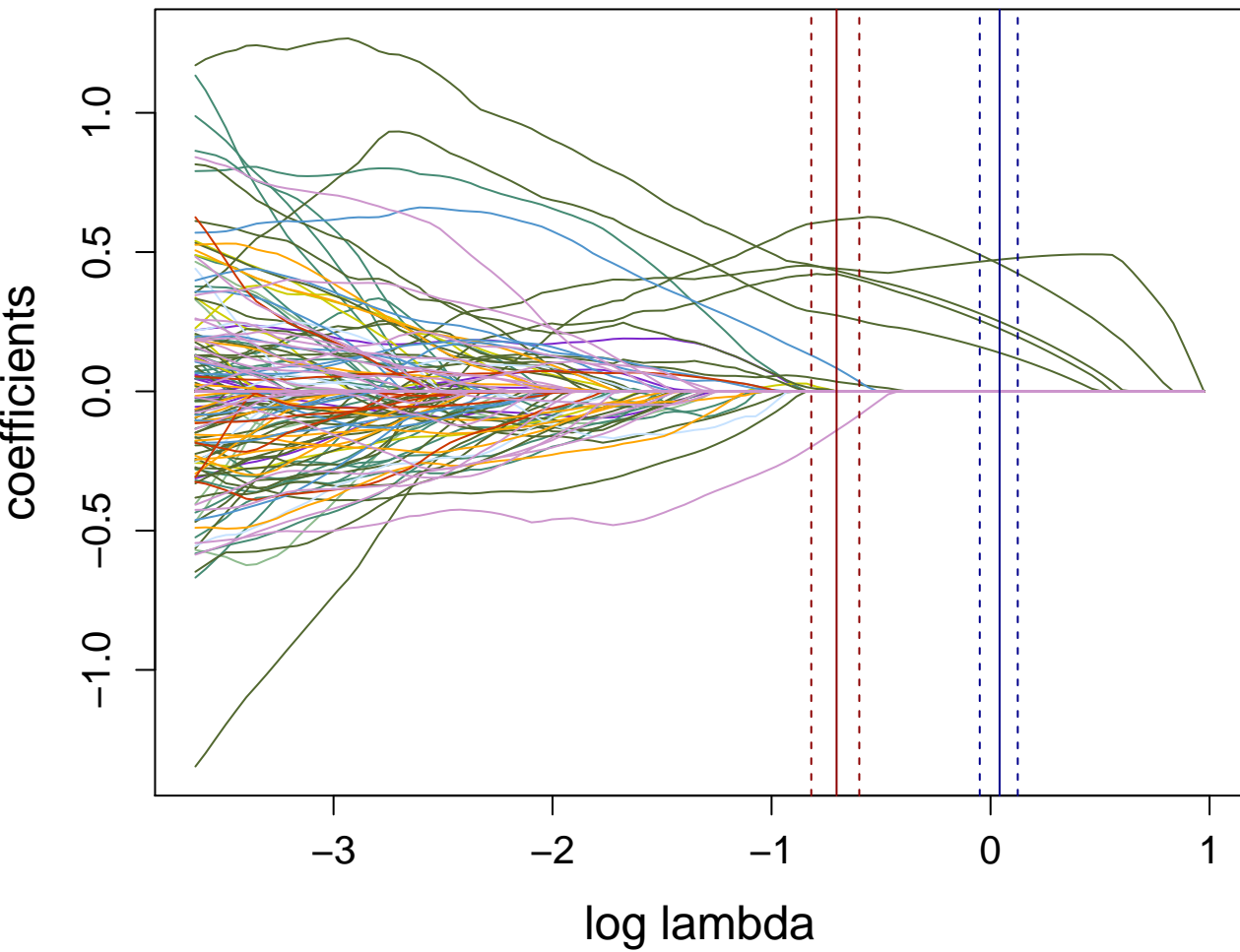

Supplement: Supplementary file 5 — Dataset EV3 [file MSB-14-e7881-s005.zip › dataset_EV3/Fc.array/CD107a/coeff-path.pdf]

Mean-Squared Error

141 127 106 93 78 64 49 33 21 11 8 5 5 5 5 3 1

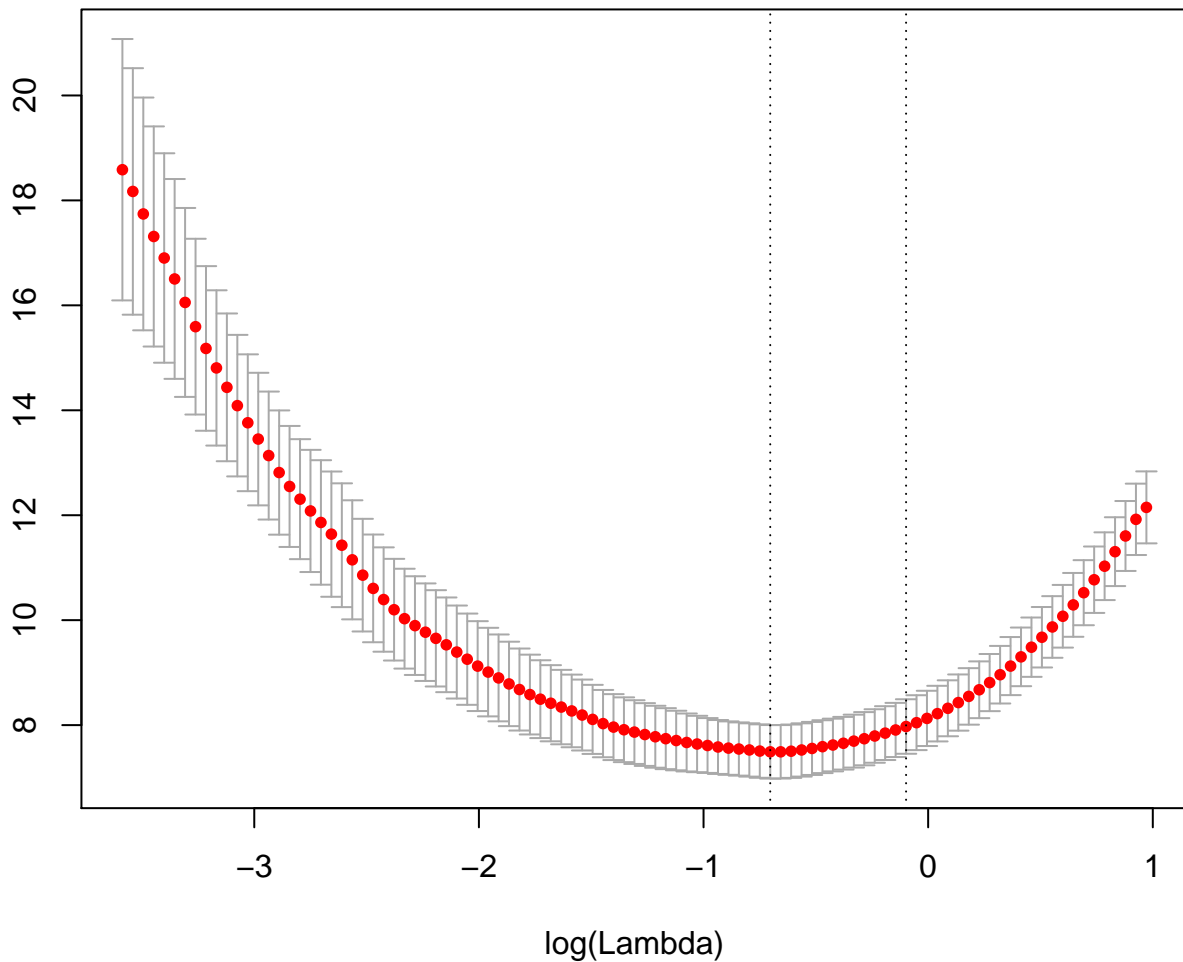

Supplement: Supplementary file 5 — Dataset EV3 [file MSB-14-e7881-s005.zip › dataset_EV3/Fc.array/CD107a/lambda.min/cv-results.pdf]

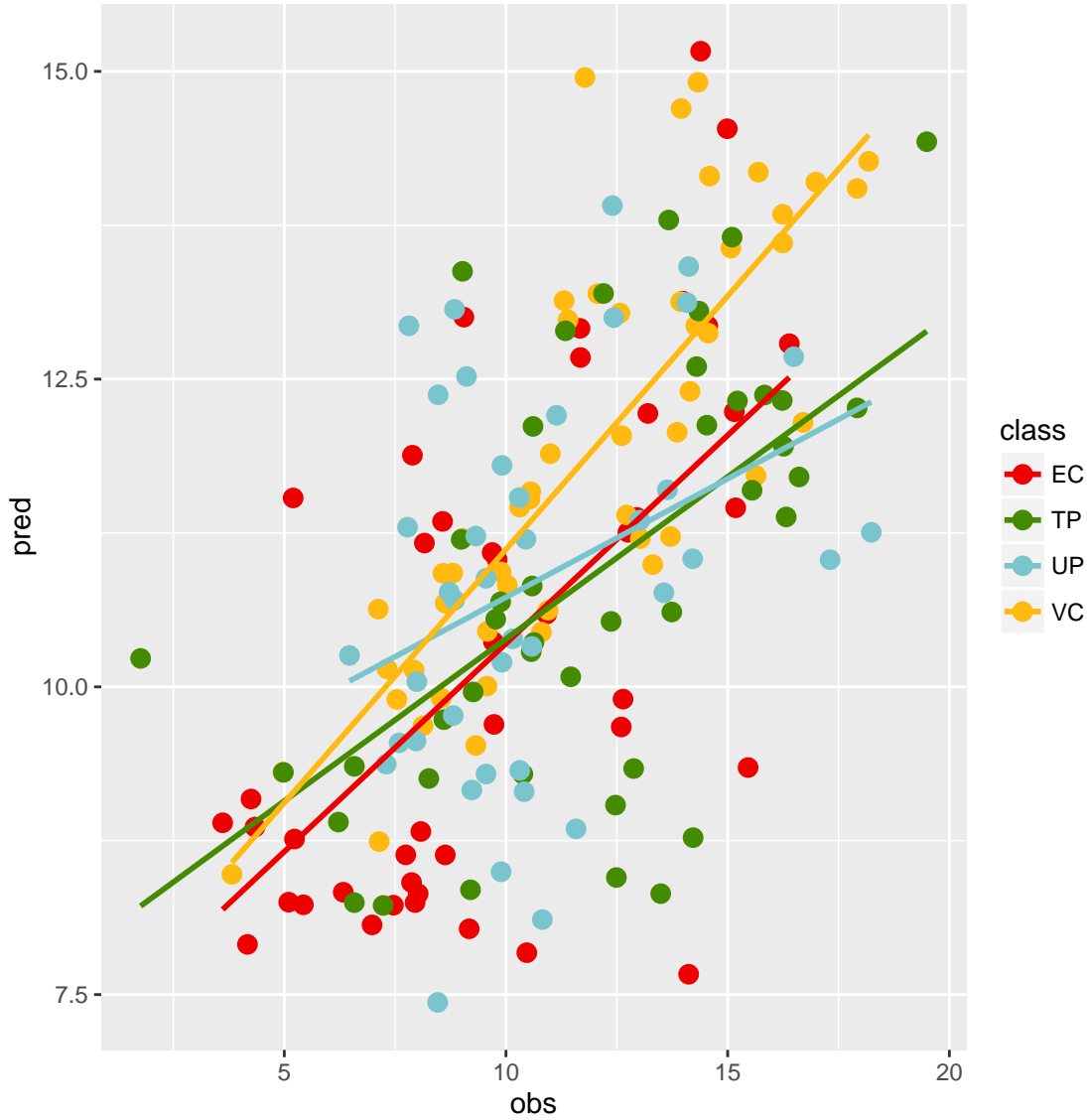

Supplement: Supplementary file 5 — Dataset EV3 [file MSB-14-e7881-s005.zip › dataset_EV3/Fc.array/CD107a/lambda.min/cvmod-scatter.pdf]

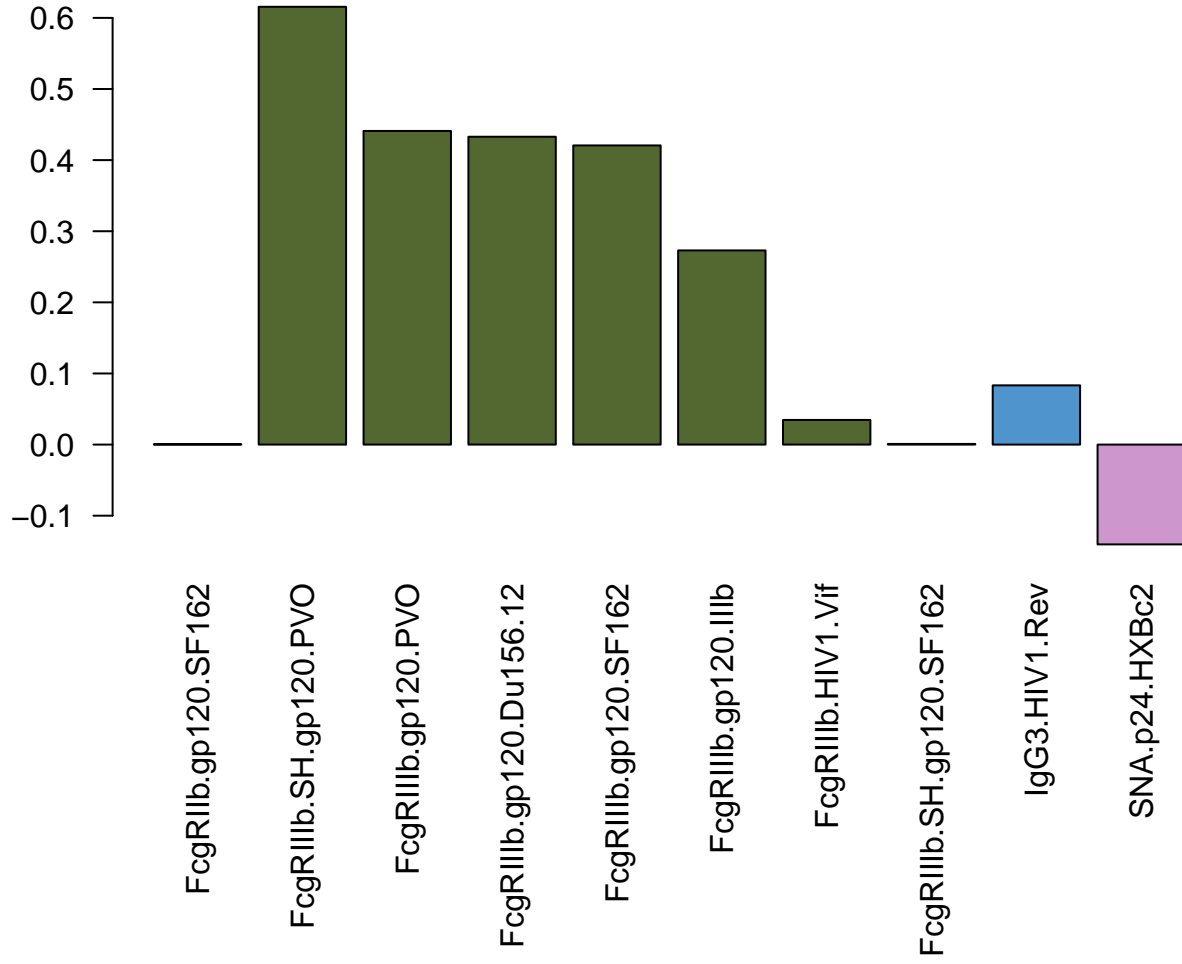

Supplement: Supplementary file 5 — Dataset EV3 [file MSB-14-e7881-s005.zip › dataset_EV3/Fc.array/CD107a/lambda.min/fullmod-coeffs.pdf]

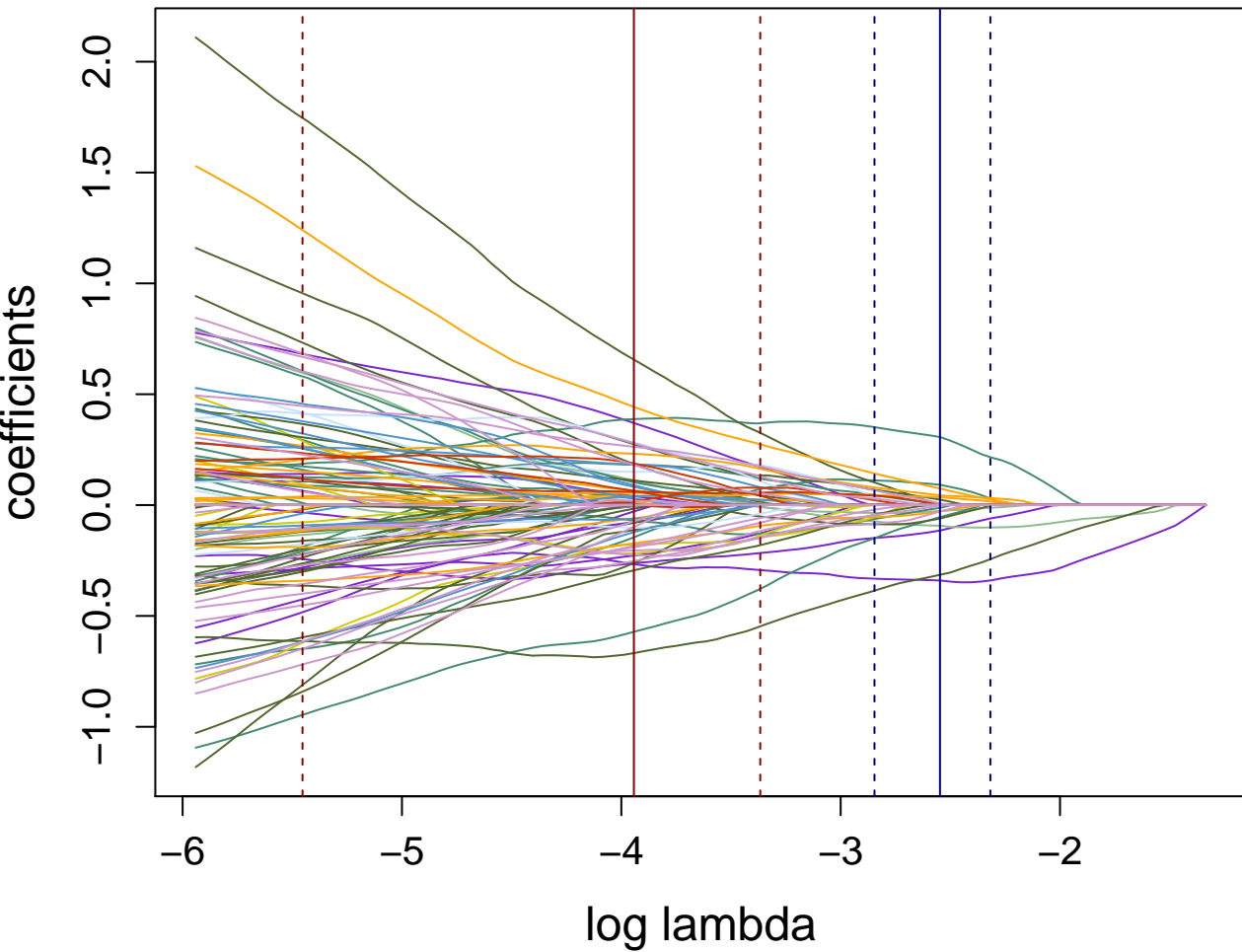

Supplement: Supplementary file 5 — Dataset EV3 [file MSB-14-e7881-s005.zip › dataset_EV3/Fc.array/class.cp/coeff-path.pdf]

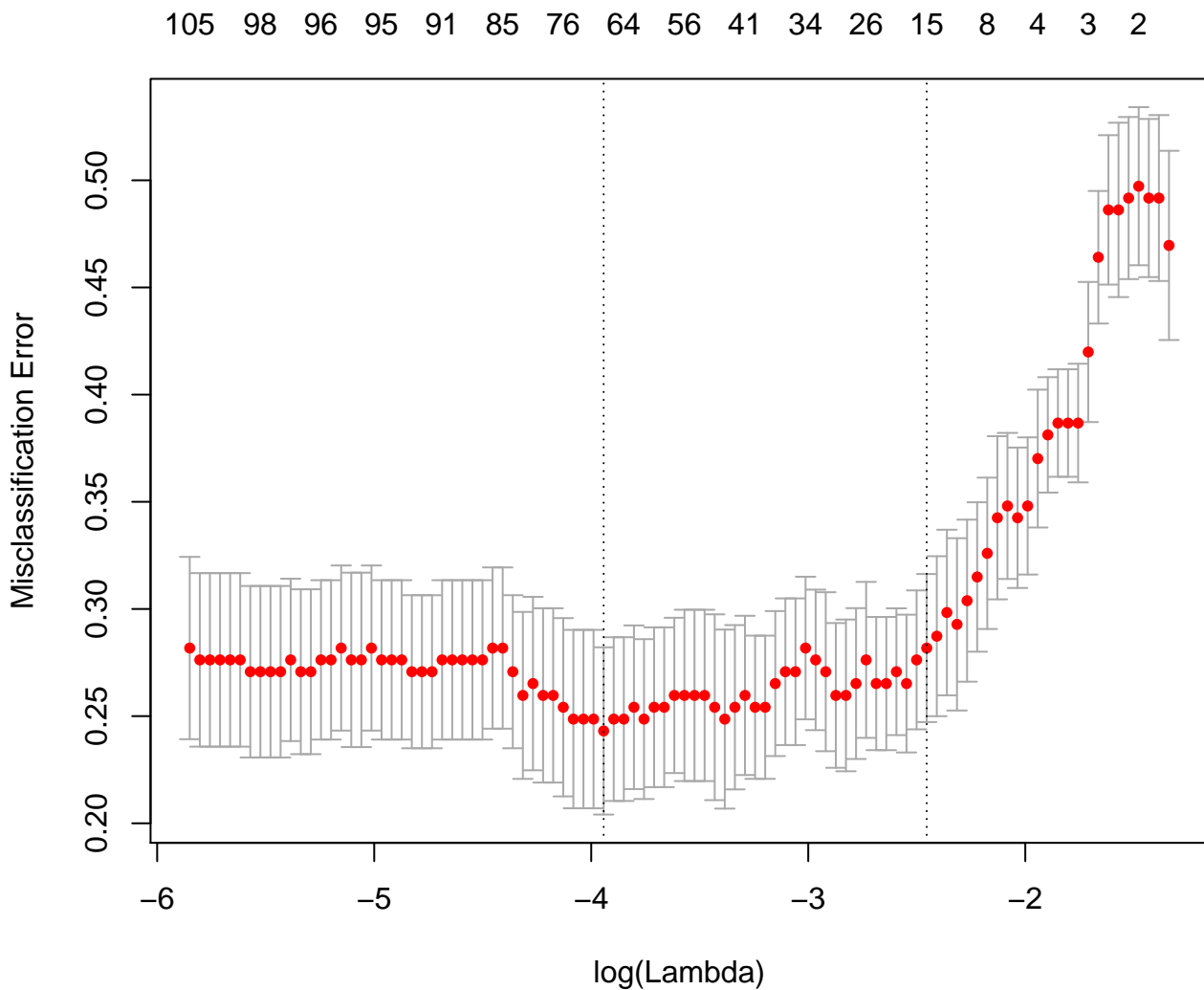

Supplement: Supplementary file 5 — Dataset EV3 [file MSB-14-e7881-s005.zip › dataset_EV3/Fc.array/class.cp/lambda.min/cv-results.pdf]

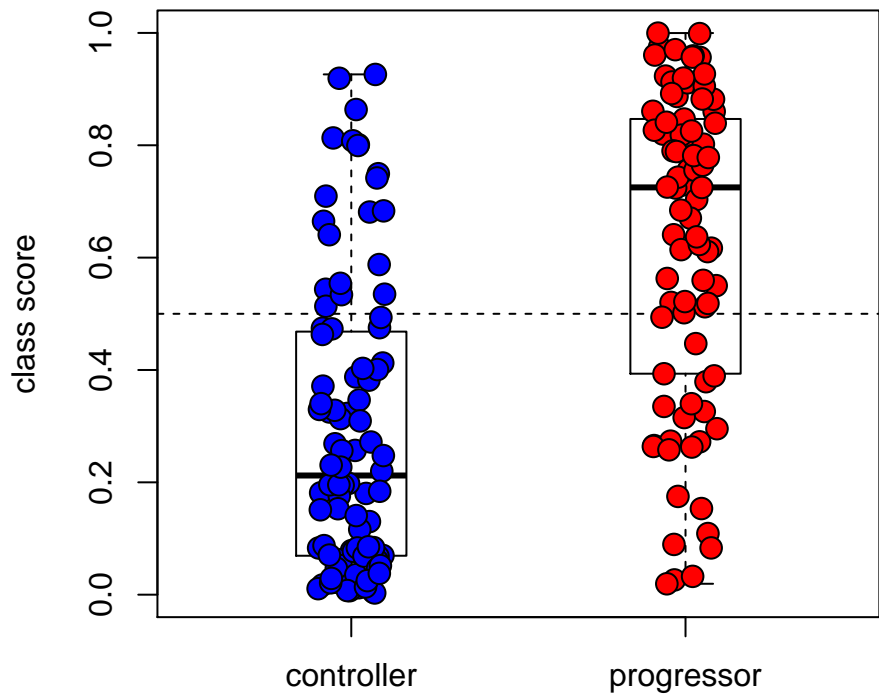

Supplement: Supplementary file 5 — Dataset EV3 [file MSB-14-e7881-s005.zip › dataset_EV3/Fc.array/class.cp/lambda.min/cvmod-box.pdf]

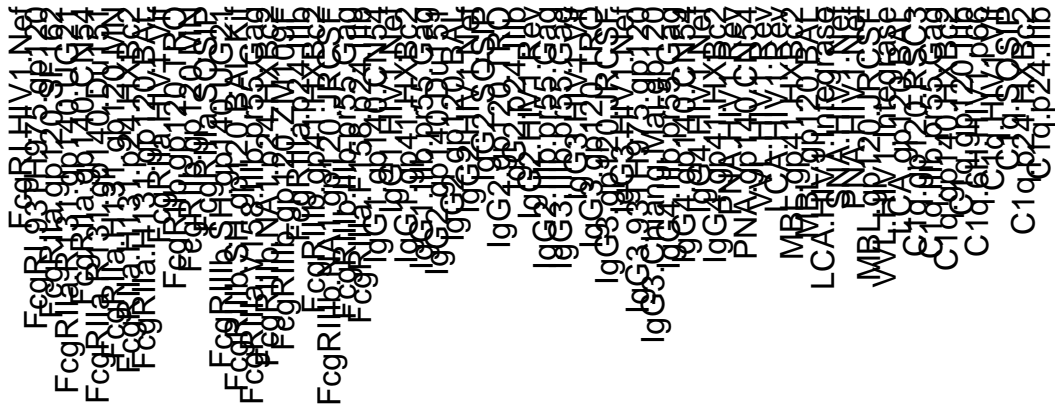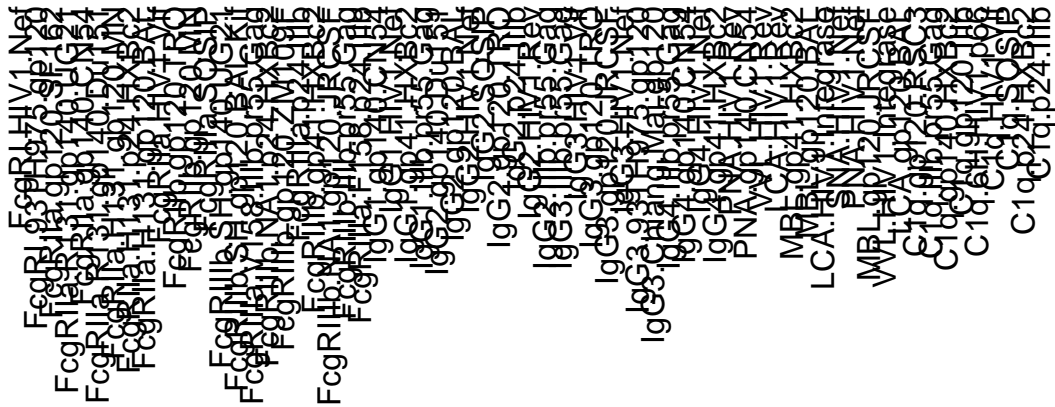

Supplement: Supplementary file 5 — Dataset EV3 [file MSB-14-e7881-s005.zip › dataset_EV3/Fc.array/class.cp/lambda.min/fullmod-coeffs.pdf]

**EC**

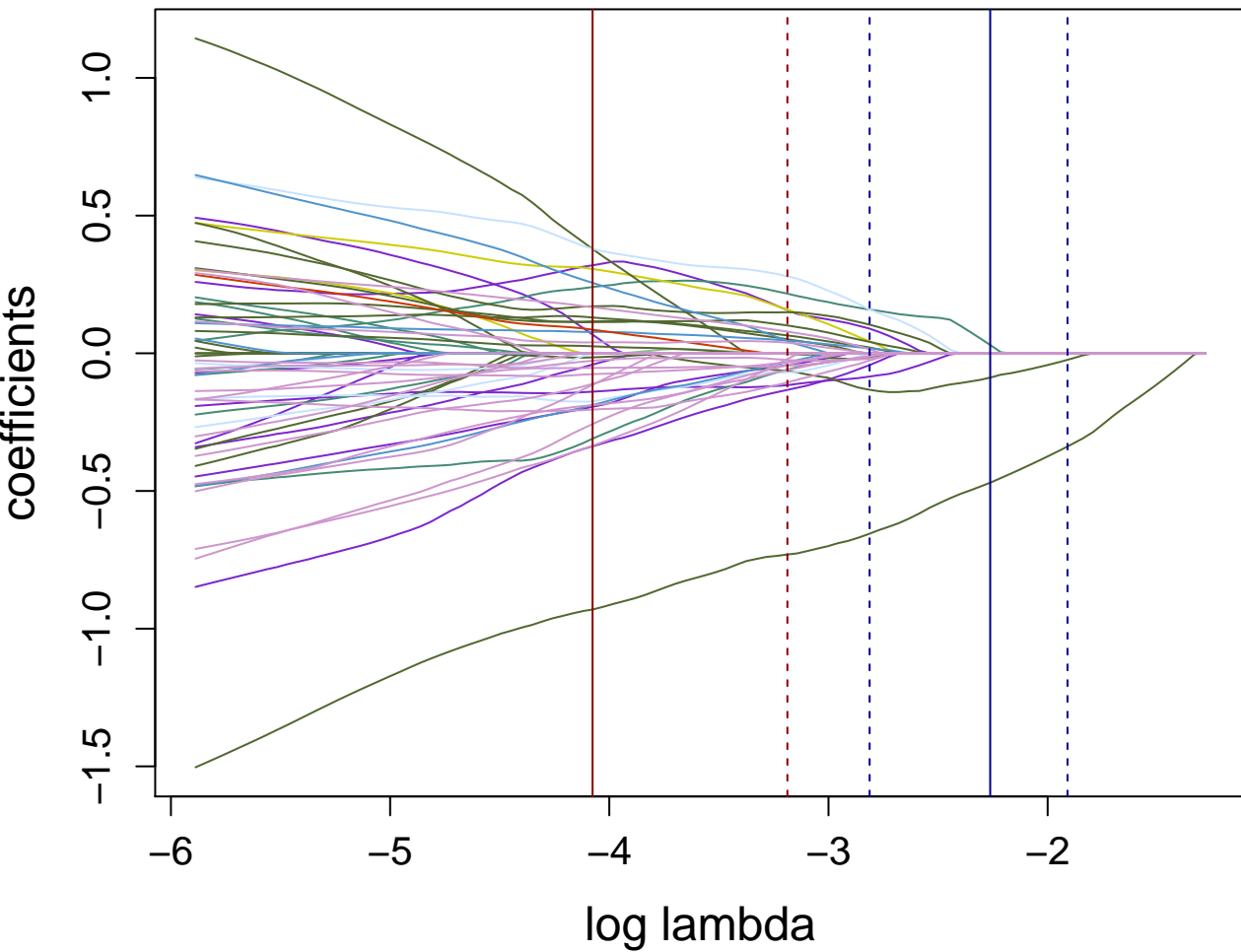

TP

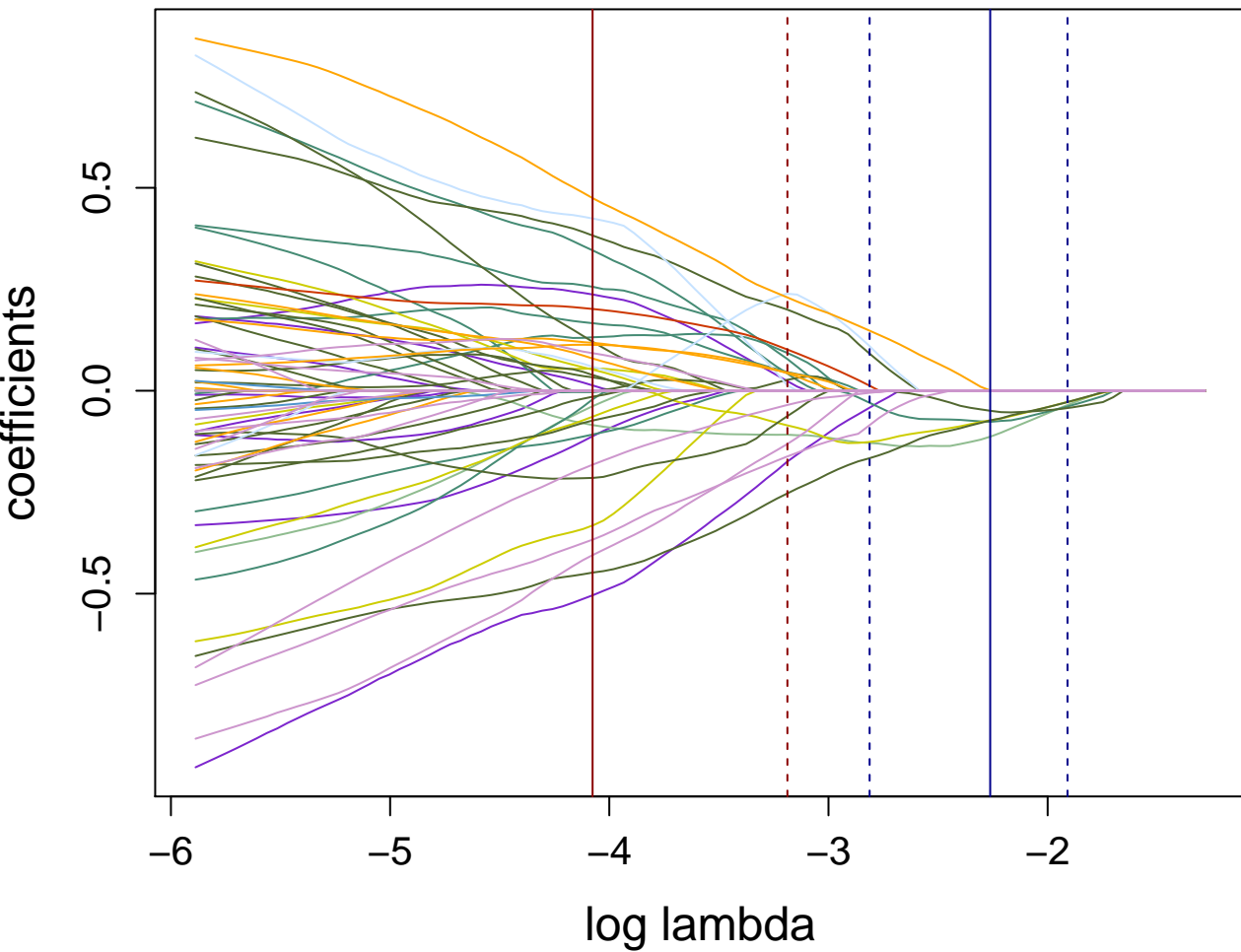

UP

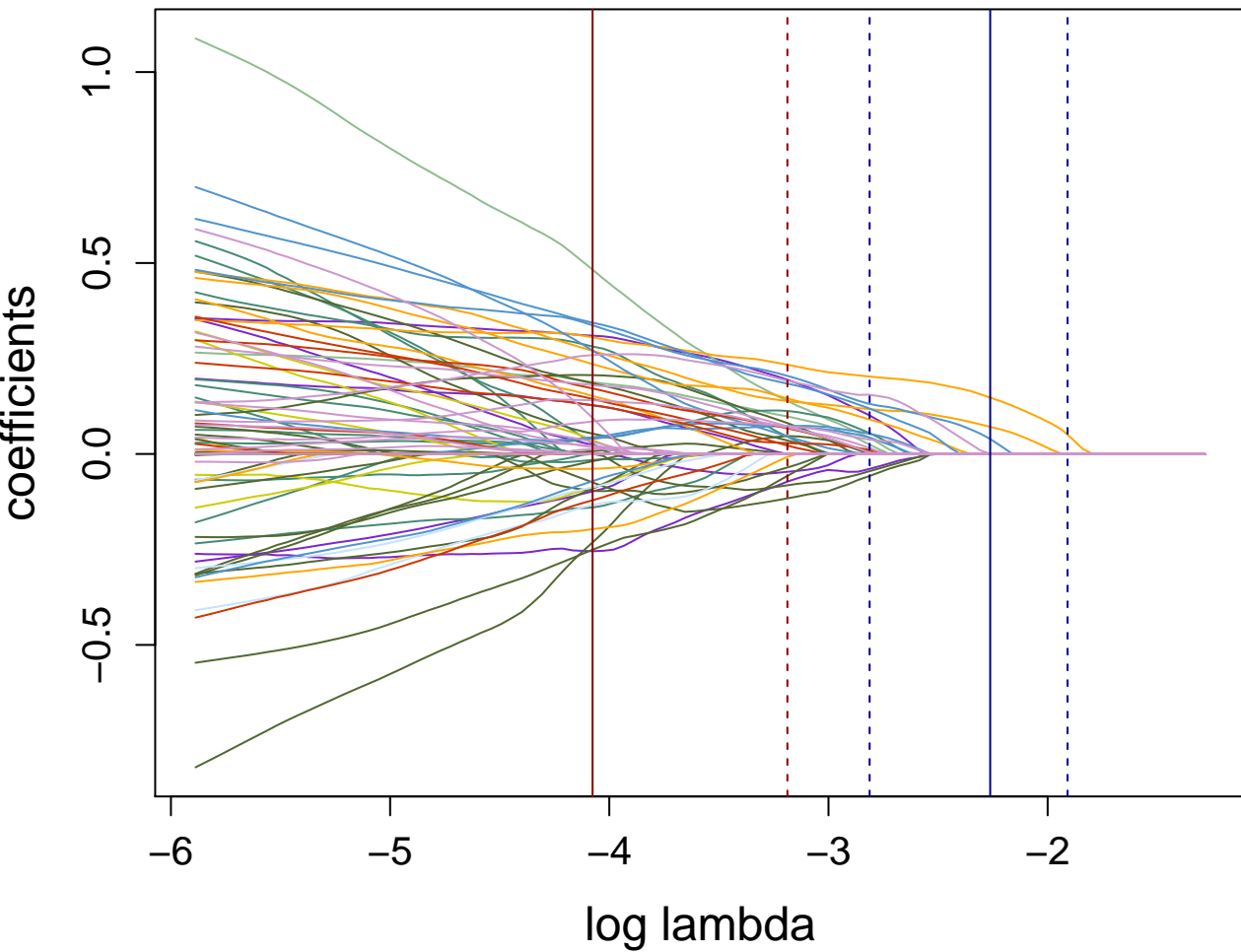

VC

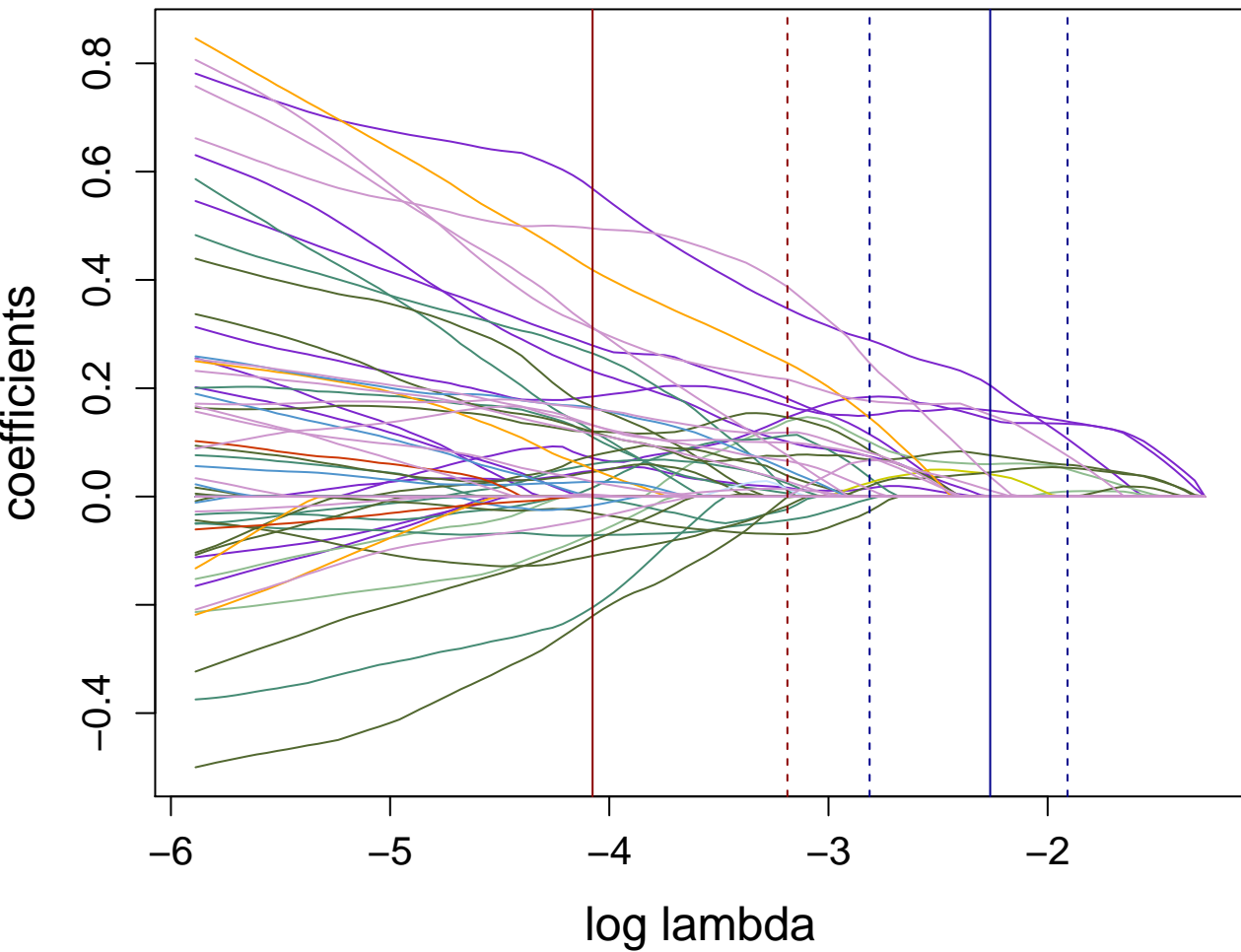

Supplement: Supplementary file 5 — Dataset EV3 [file MSB-14-e7881-s005.zip › dataset_EV3/Fc.array/class.etuv/coeff-path.pdf]

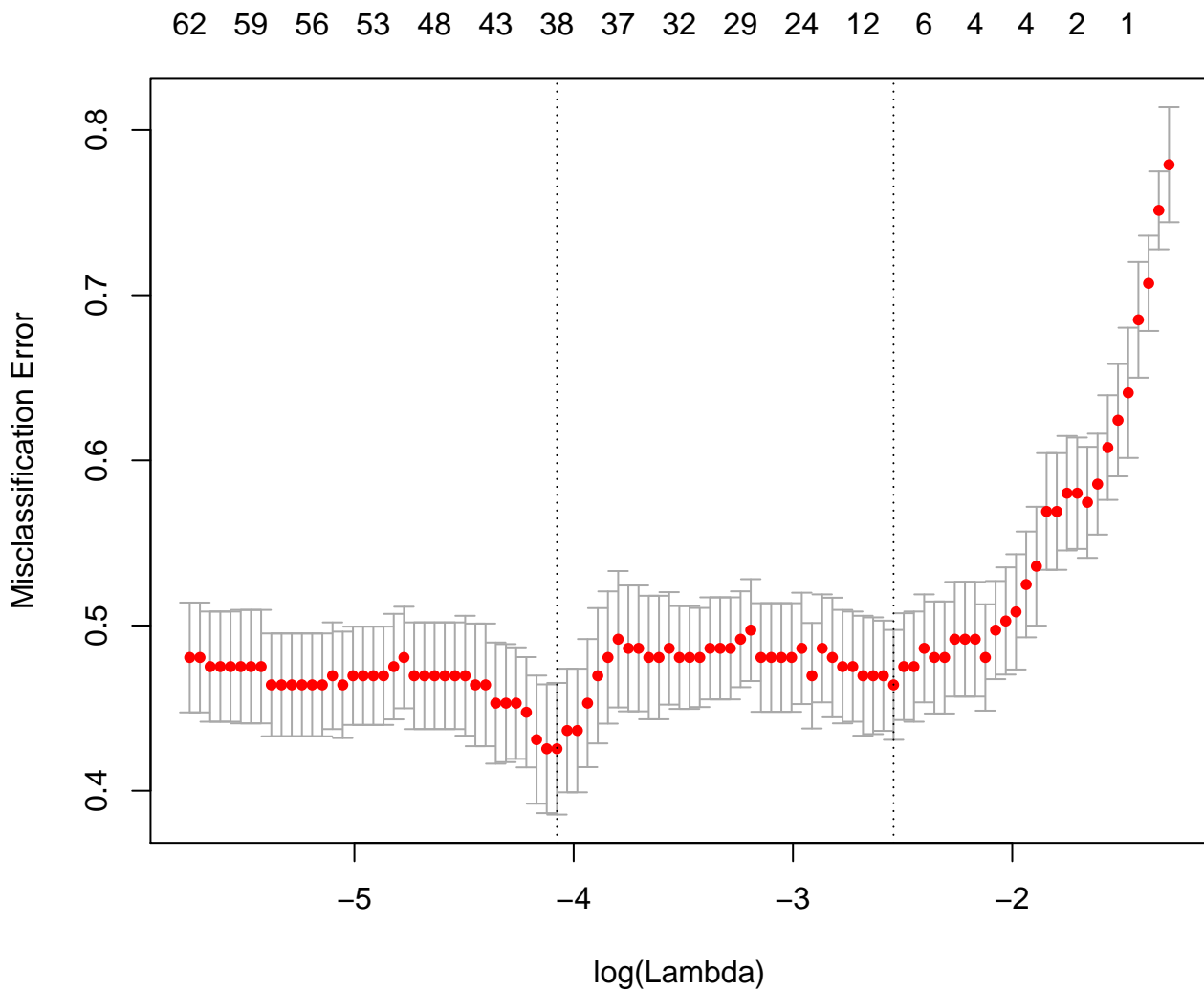

Supplement: Supplementary file 5 — Dataset EV3 [file MSB-14-e7881-s005.zip › dataset_EV3/Fc.array/class.etuv/lambda.min/cv-results.pdf]

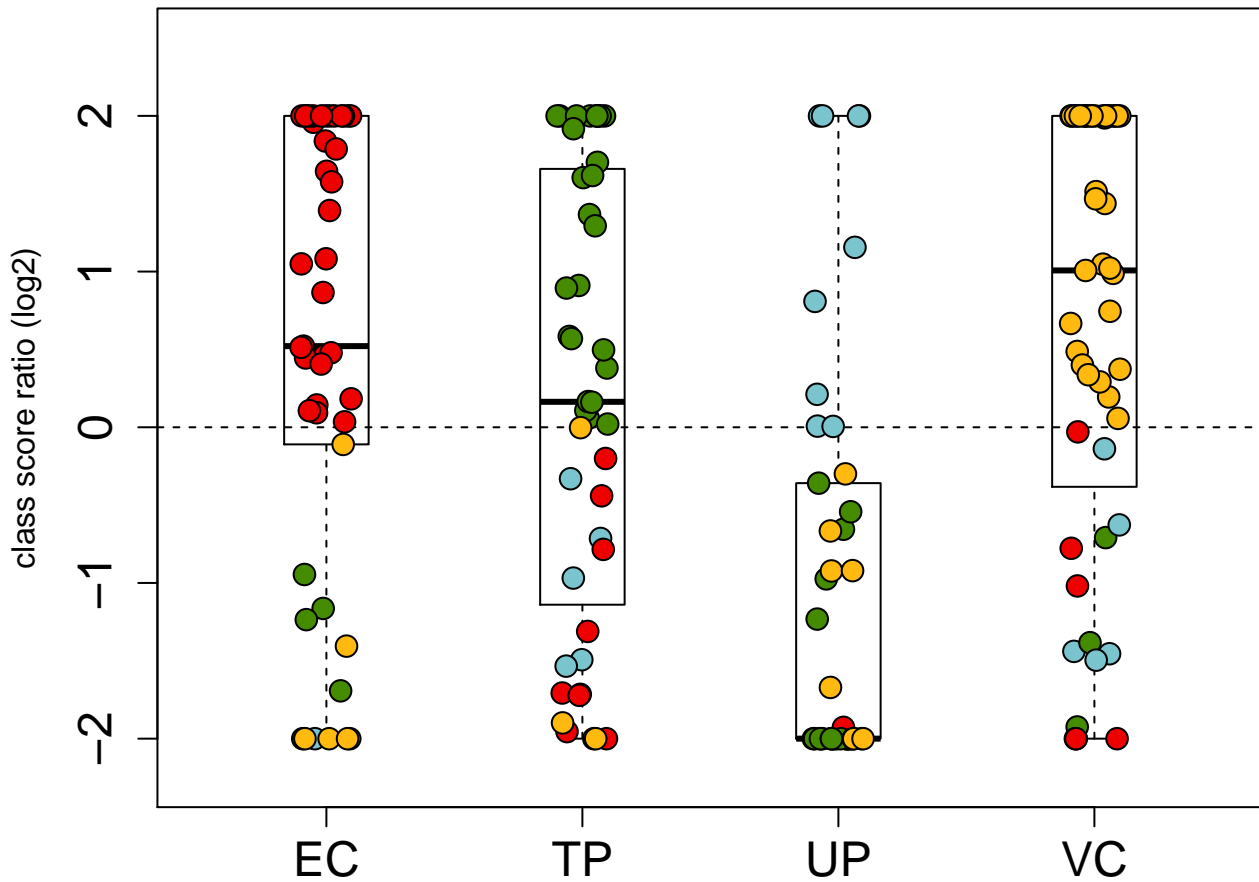

Supplement: Supplementary file 5 — Dataset EV3 [file MSB-14-e7881-s005.zip › dataset_EV3/Fc.array/class.etuv/lambda.min/cvmod-box.pdf]

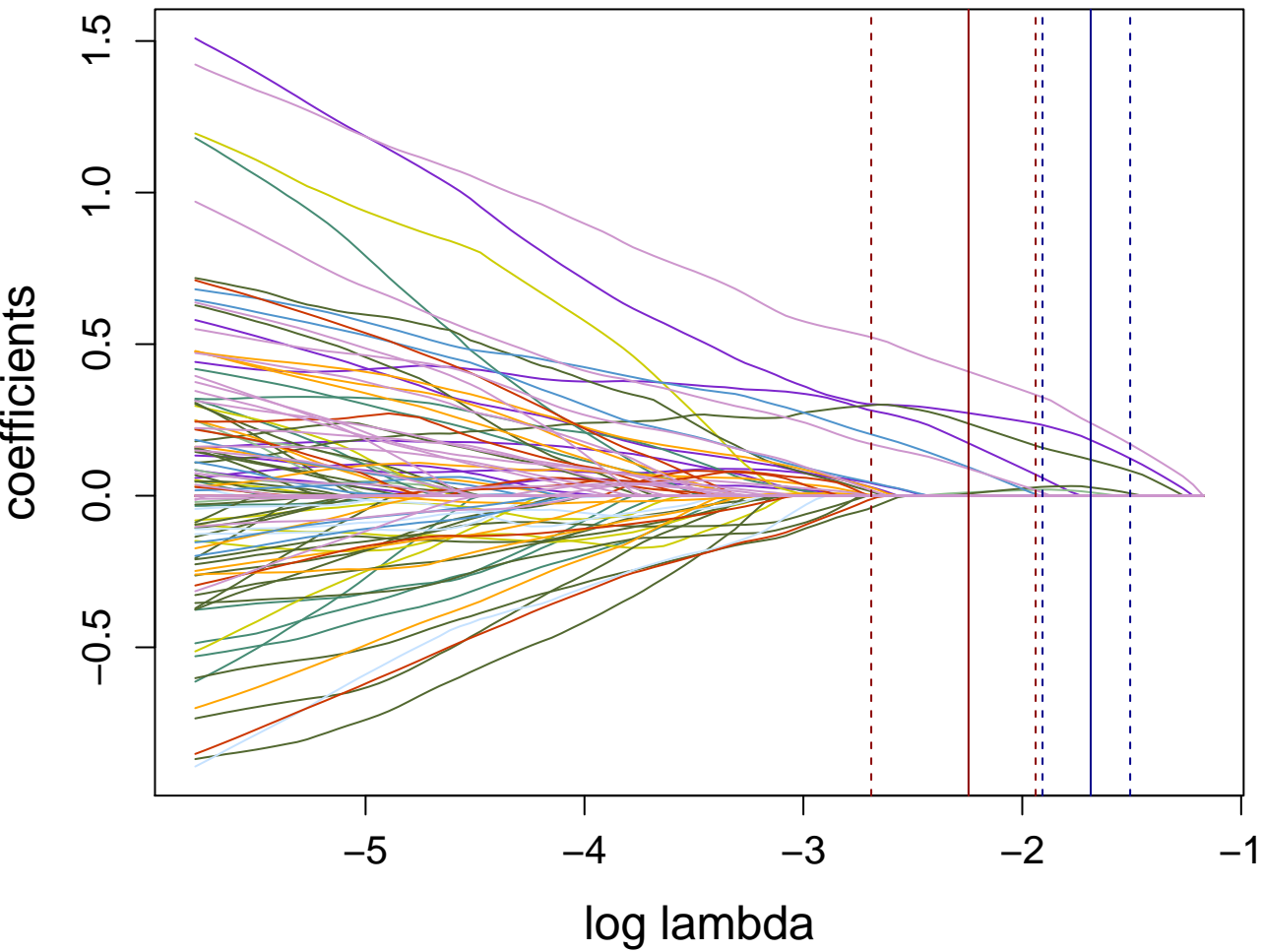

Supplement: Supplementary file 5 — Dataset EV3 [file MSB-14-e7881-s005.zip › dataset_EV3/Fc.array/class.nv/coeff-path.pdf]

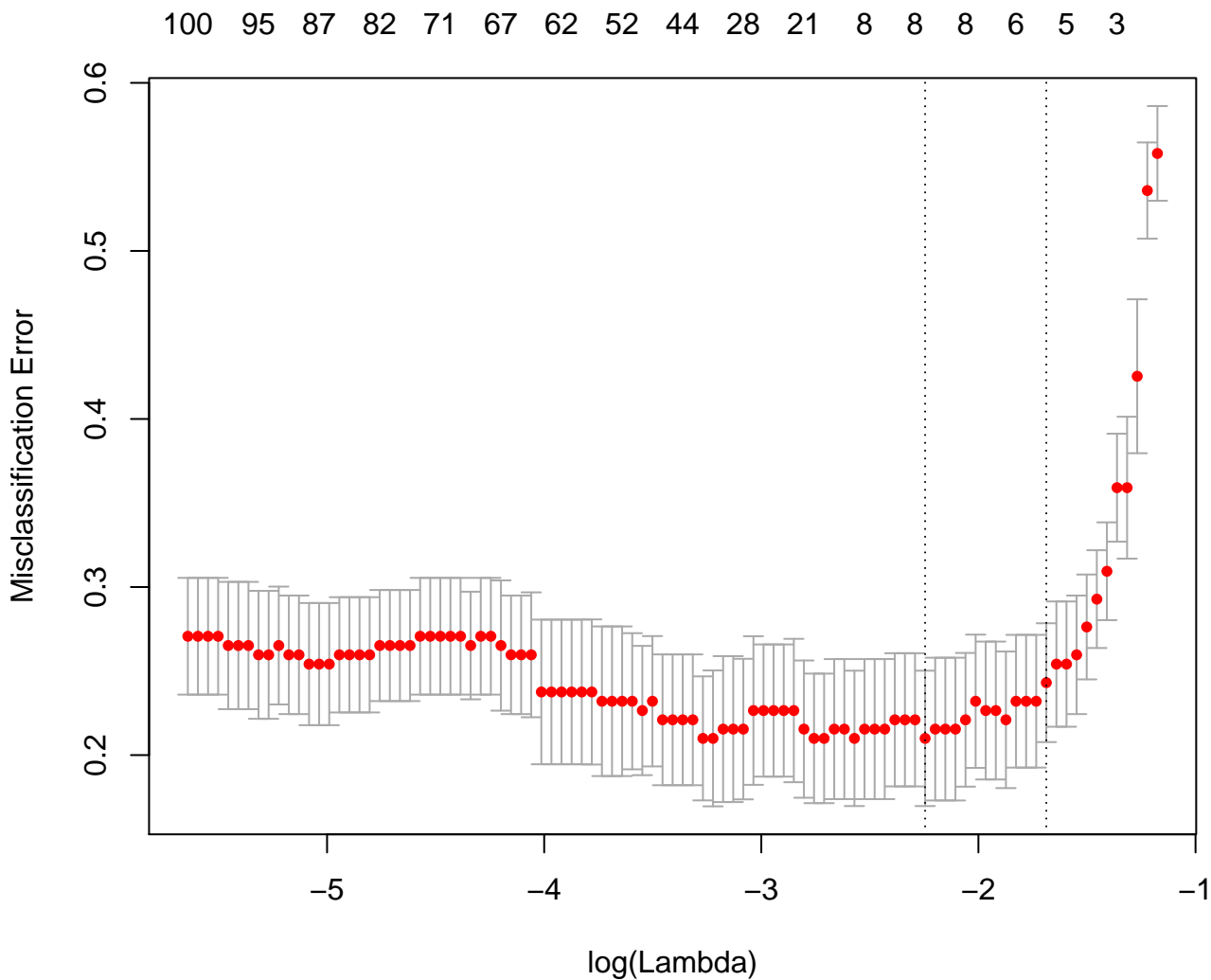

Supplement: Supplementary file 5 — Dataset EV3 [file MSB-14-e7881-s005.zip › dataset_EV3/Fc.array/class.nv/lambda.min/cv-results.pdf]

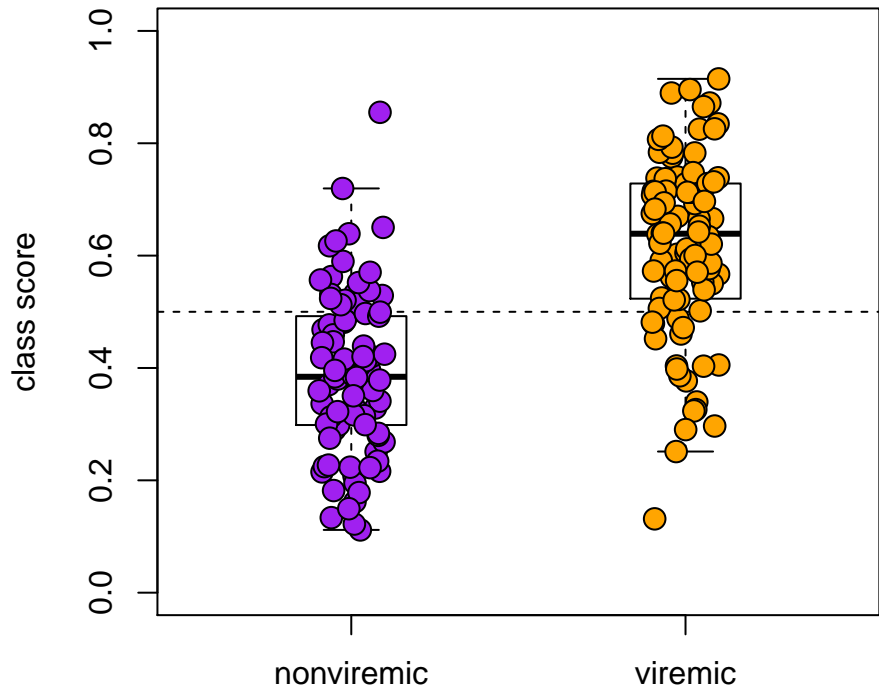

Supplement: Supplementary file 5 — Dataset EV3 [file MSB-14-e7881-s005.zip › dataset_EV3/Fc.array/class.nv/lambda.min/cvmod-box.pdf]

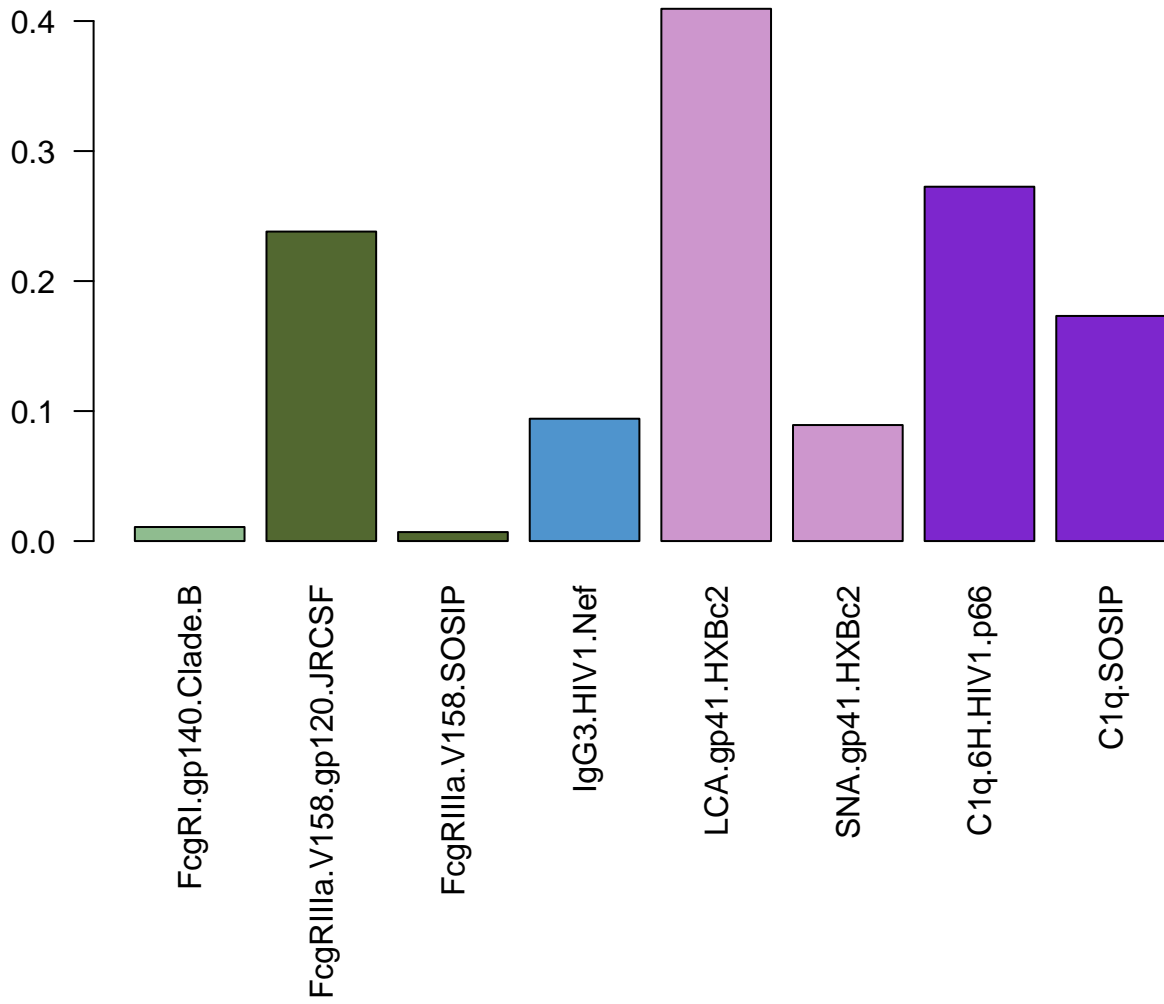

Supplement: Supplementary file 5 — Dataset EV3 [file MSB-14-e7881-s005.zip › dataset_EV3/Fc.array/class.nv/lambda.min/fullmod-coeffs.pdf]

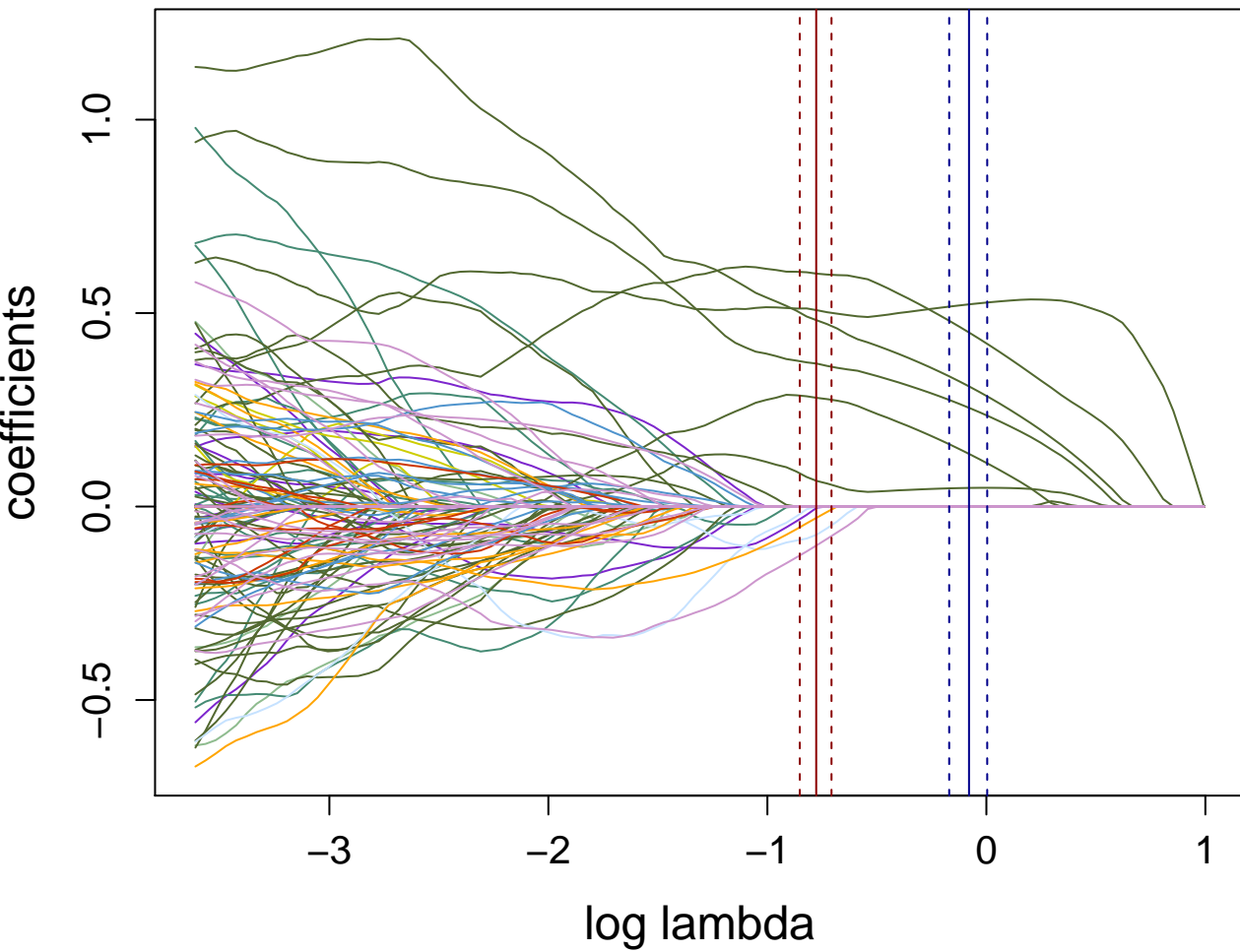

Supplement: Supplementary file 5 — Dataset EV3 [file MSB-14-e7881-s005.zip › dataset_EV3/Fc.array/IFNy/coeff-path.pdf]

Mean-Squared Error

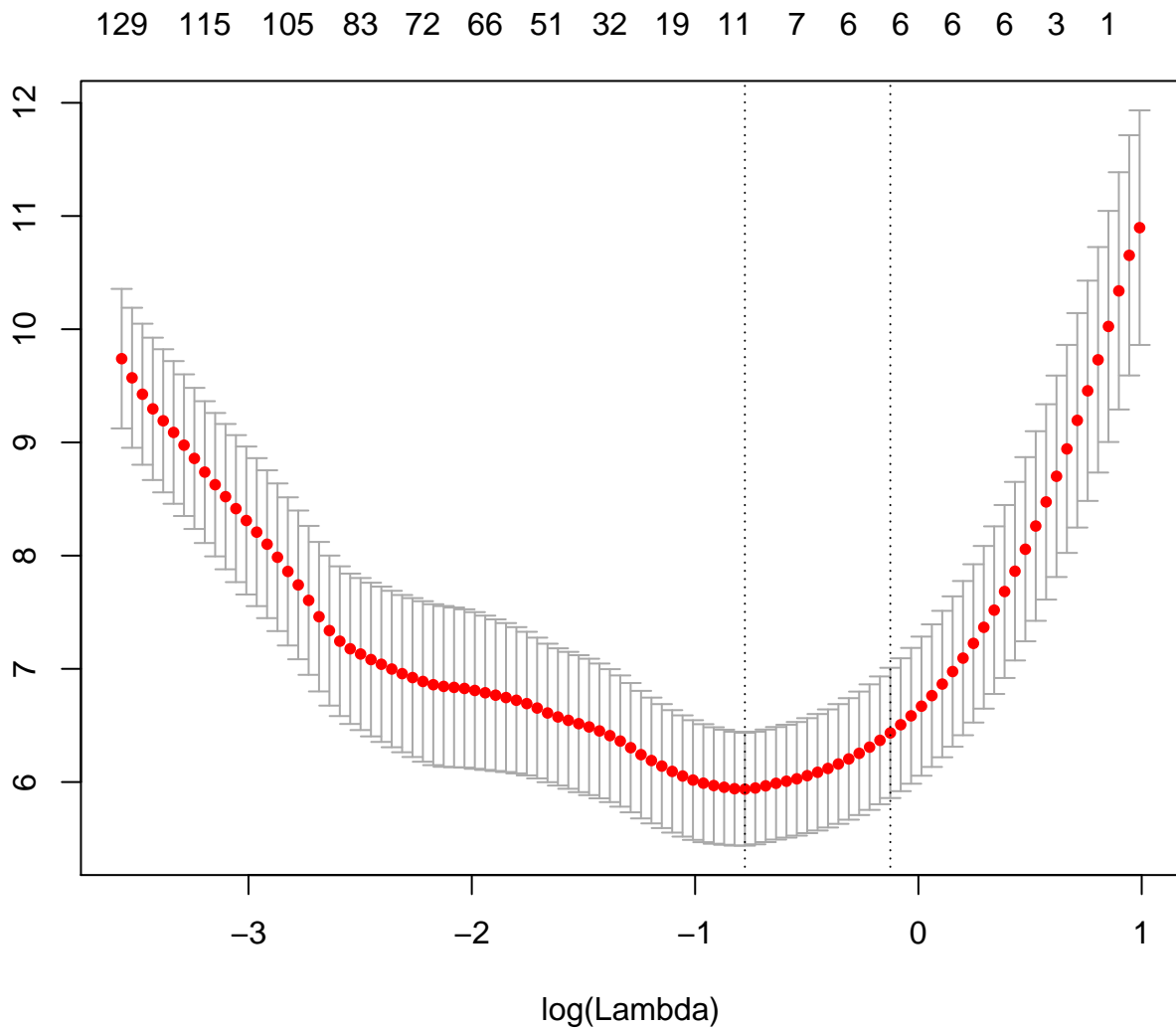

Supplement: Supplementary file 5 — Dataset EV3 [file MSB-14-e7881-s005.zip › dataset_EV3/Fc.array/IFNy/lambda.min/cv-results.pdf]

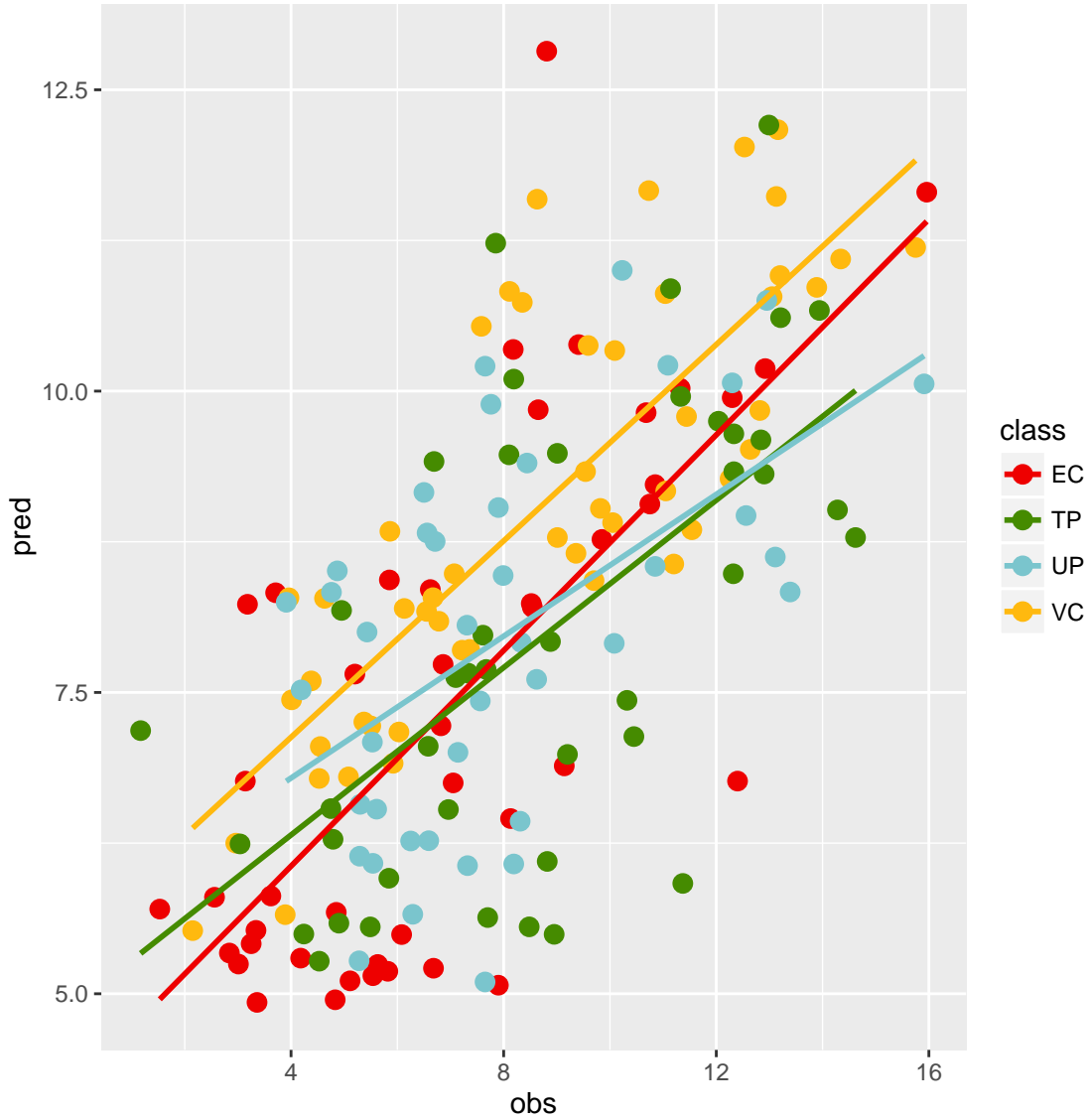

Supplement: Supplementary file 5 — Dataset EV3 [file MSB-14-e7881-s005.zip › dataset_EV3/Fc.array/IFNy/lambda.min/cvmod-scatter.pdf]

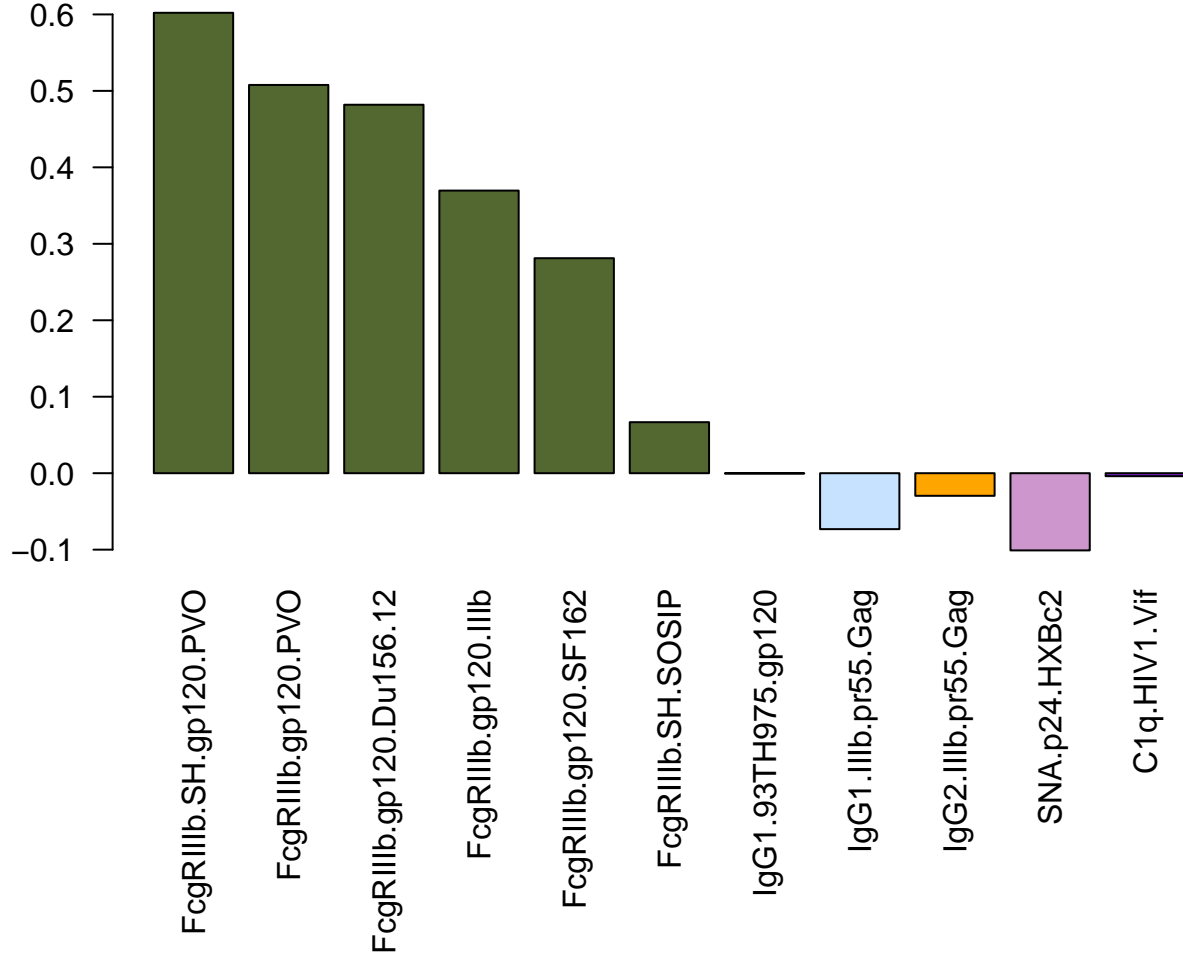

Supplement: Supplementary file 5 — Dataset EV3 [file MSB-14-e7881-s005.zip › dataset_EV3/Fc.array/IFNy/lambda.min/fullmod-coeffs.pdf]

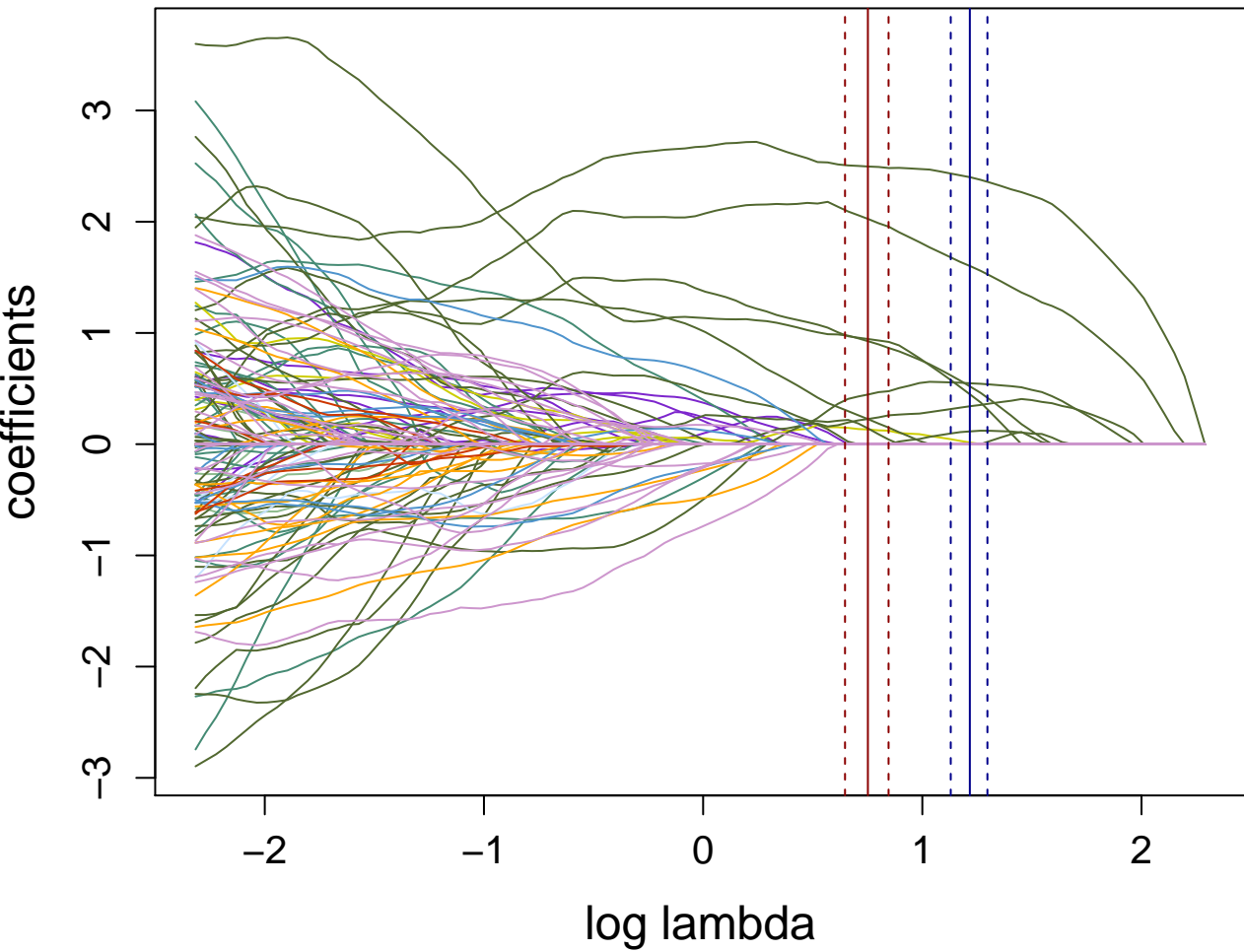

Supplement: Supplementary file 5 — Dataset EV3 [file MSB-14-e7881-s005.zip › dataset_EV3/Fc.array/MIP1b/coeff-path.pdf]

Mean-Squared Error

135 113 97 89 76 65 49 36 25 18 10 9 8 8 5 4 2

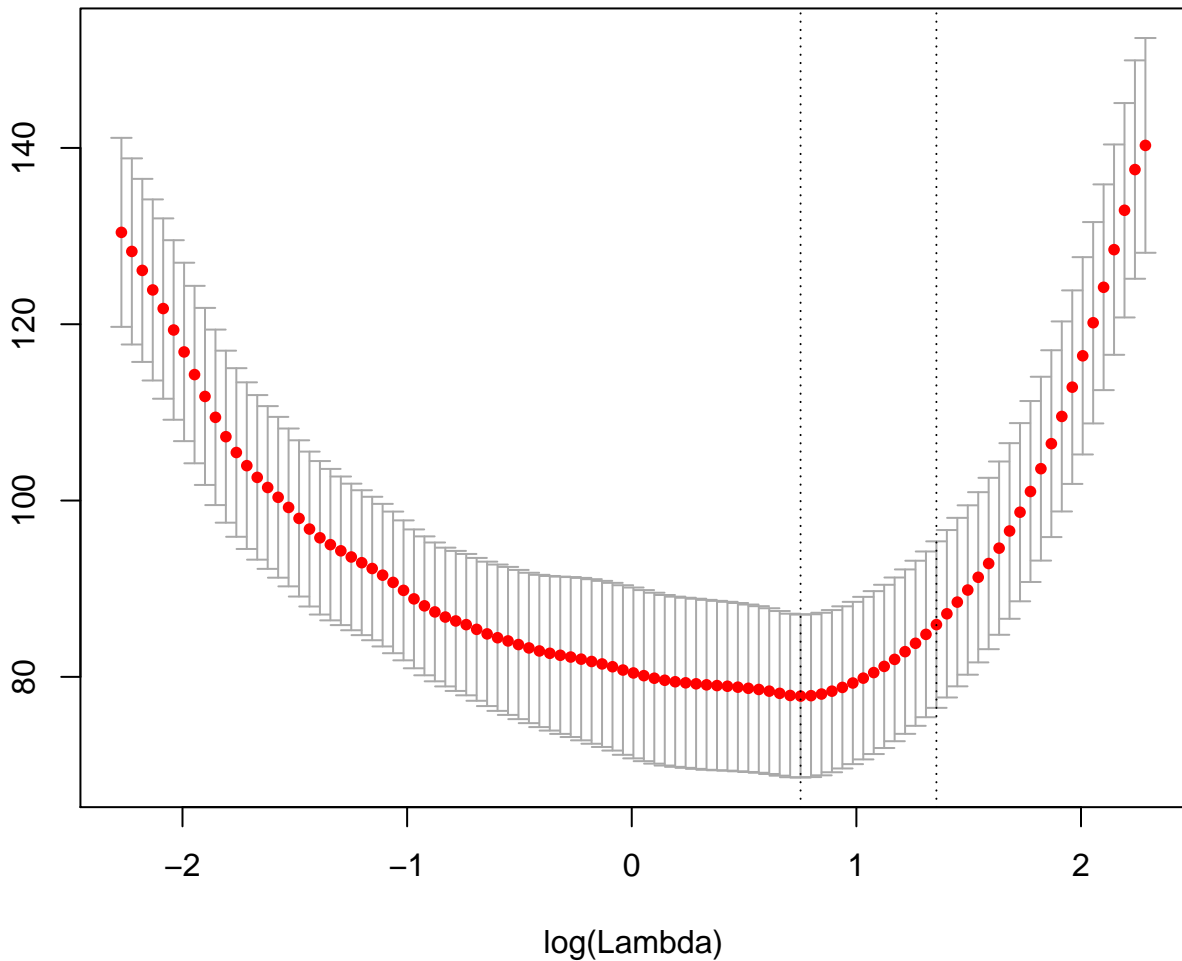

Supplement: Supplementary file 5 — Dataset EV3 [file MSB-14-e7881-s005.zip › dataset_EV3/Fc.array/MIP1b/lambda.min/cv-results.pdf]

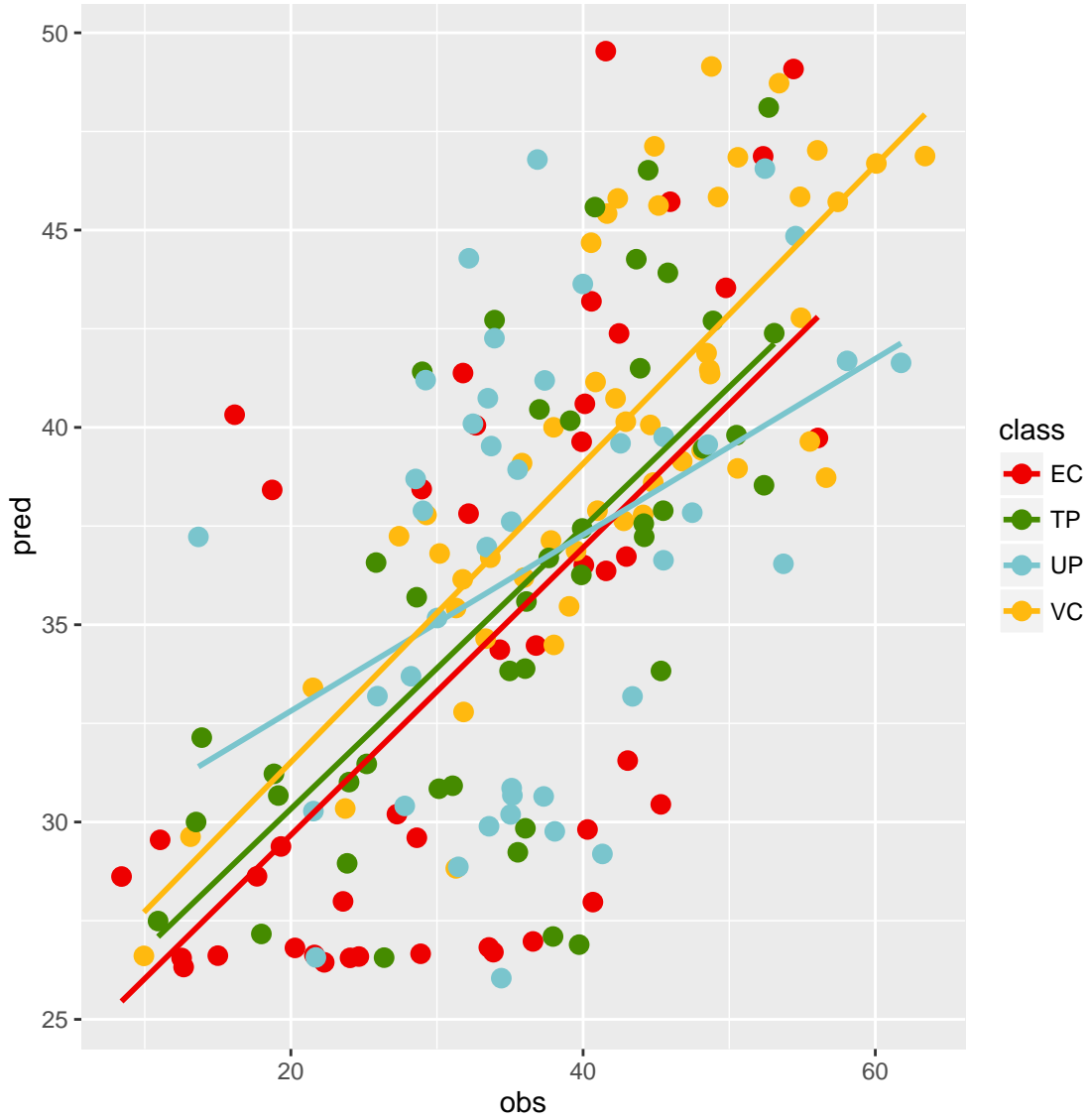

Supplement: Supplementary file 5 — Dataset EV3 [file MSB-14-e7881-s005.zip › dataset_EV3/Fc.array/MIP1b/lambda.min/cvmod-scatter.pdf]

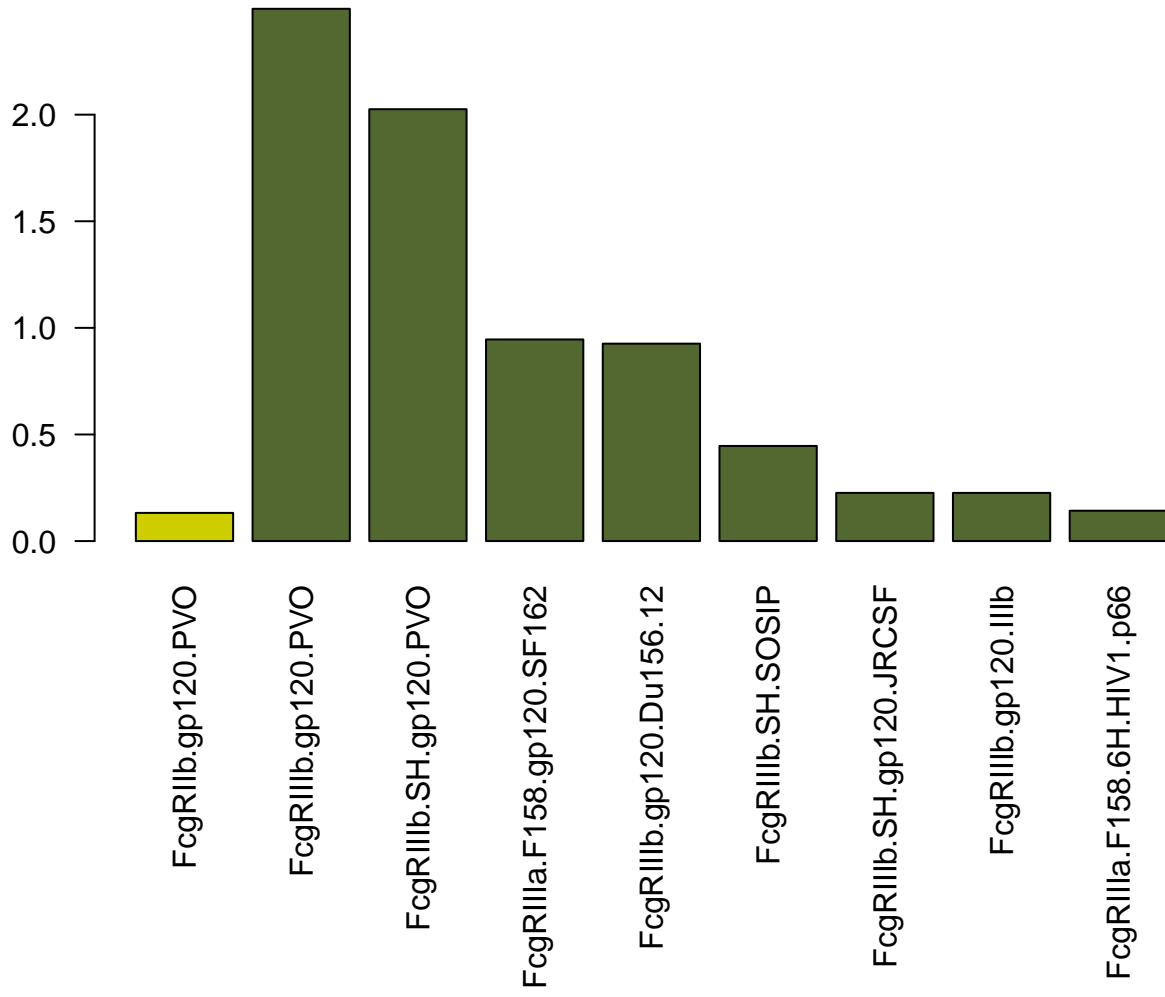

Supplement: Supplementary file 5 — Dataset EV3 [file MSB-14-e7881-s005.zip › dataset_EV3/Fc.array/MIP1b/lambda.min/fullmod-coeffs.pdf]

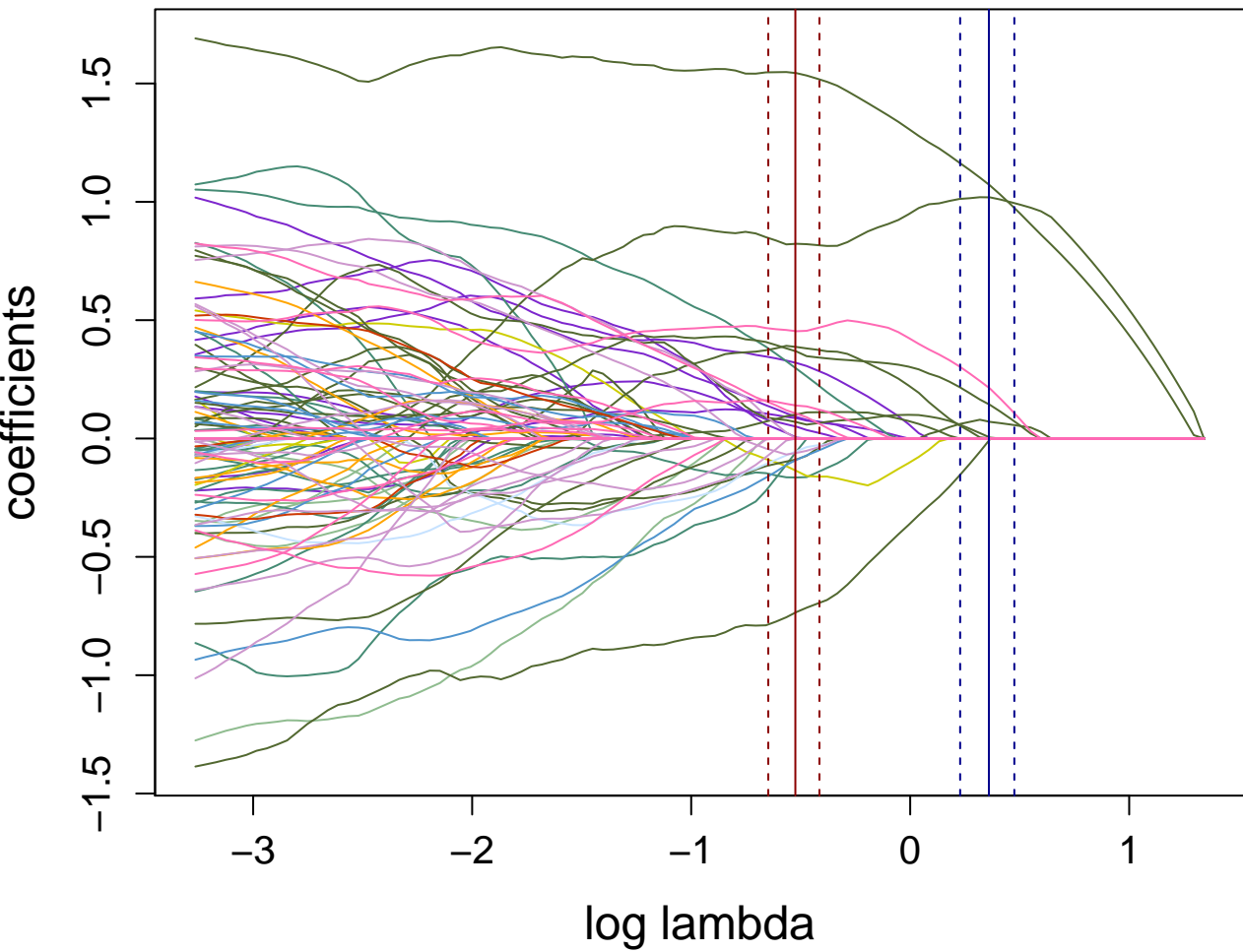

Supplement: Supplementary file 5 — Dataset EV3 [file MSB-14-e7881-s005.zip › dataset_EV3/Fc.array.plus.glycan/ADCC/coeff-path.pdf]

Mean-Squared Error

102 92 84 79 73 65 56 44 32 25 20 11 8 5 2 2 2

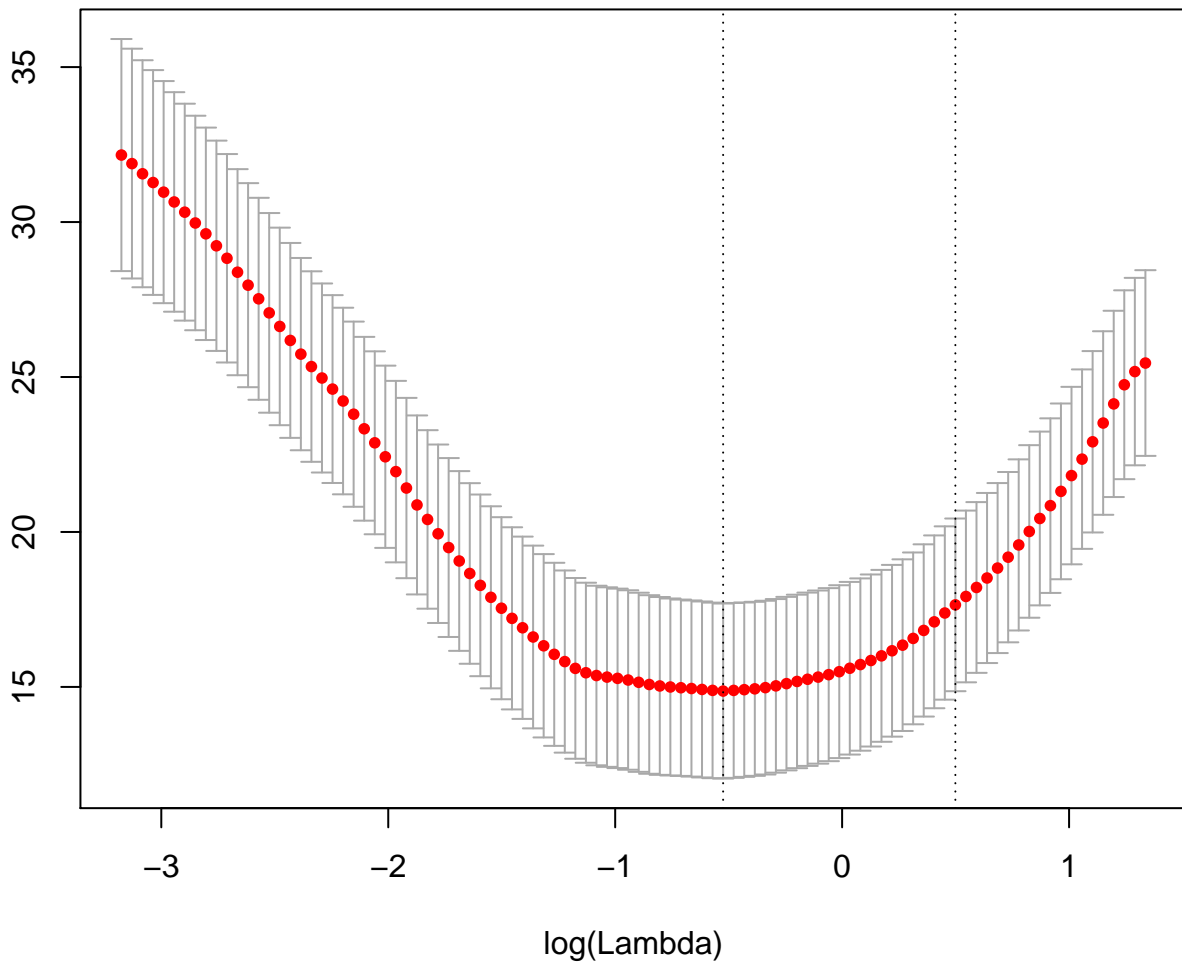

Supplement: Supplementary file 5 — Dataset EV3 [file MSB-14-e7881-s005.zip › dataset_EV3/Fc.array.plus.glycan/ADCC/lambda.min/cv-results.pdf]

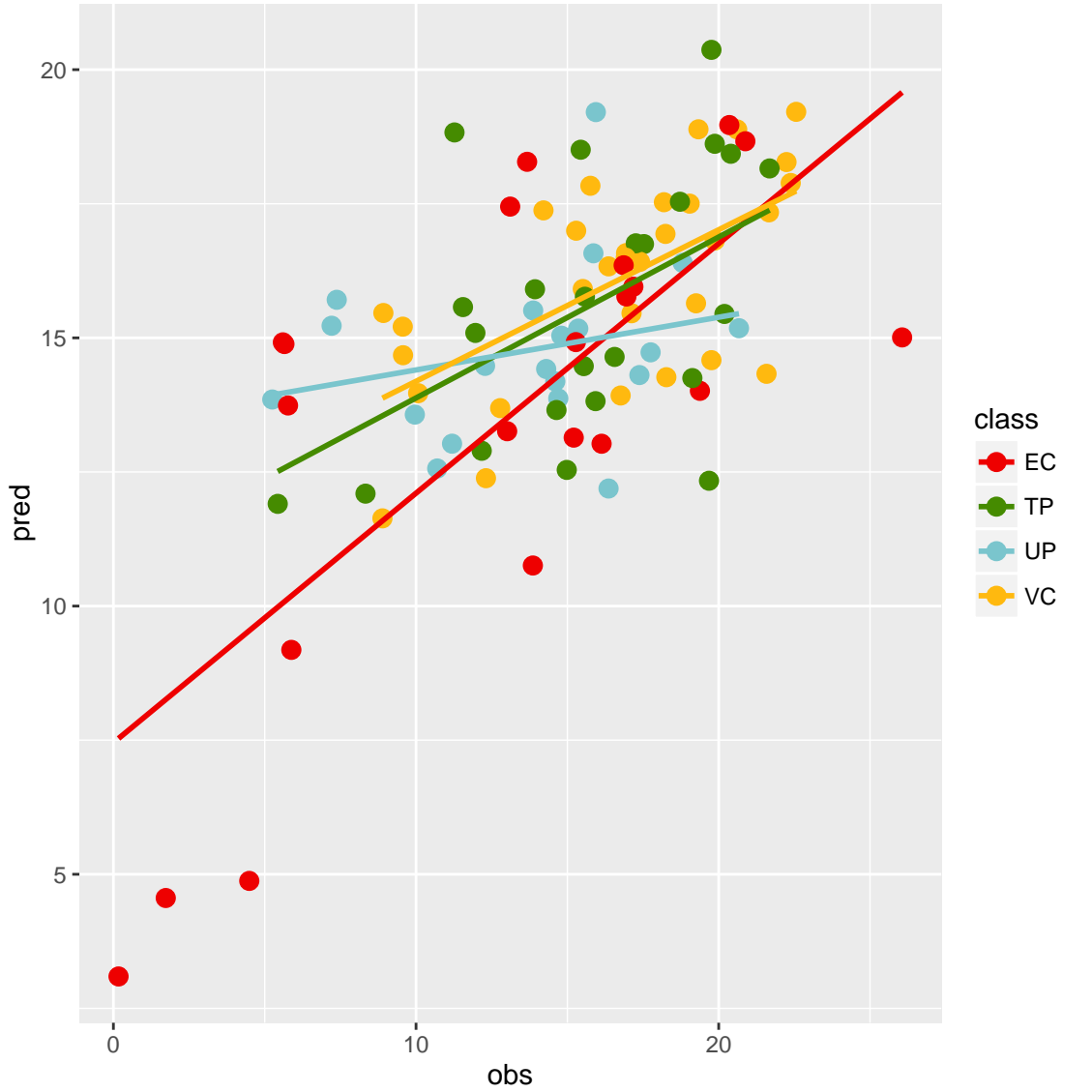

Supplement: Supplementary file 5 — Dataset EV3 [file MSB-14-e7881-s005.zip › dataset_EV3/Fc.array.plus.glycan/ADCC/lambda.min/cvmod-scatter.pdf]

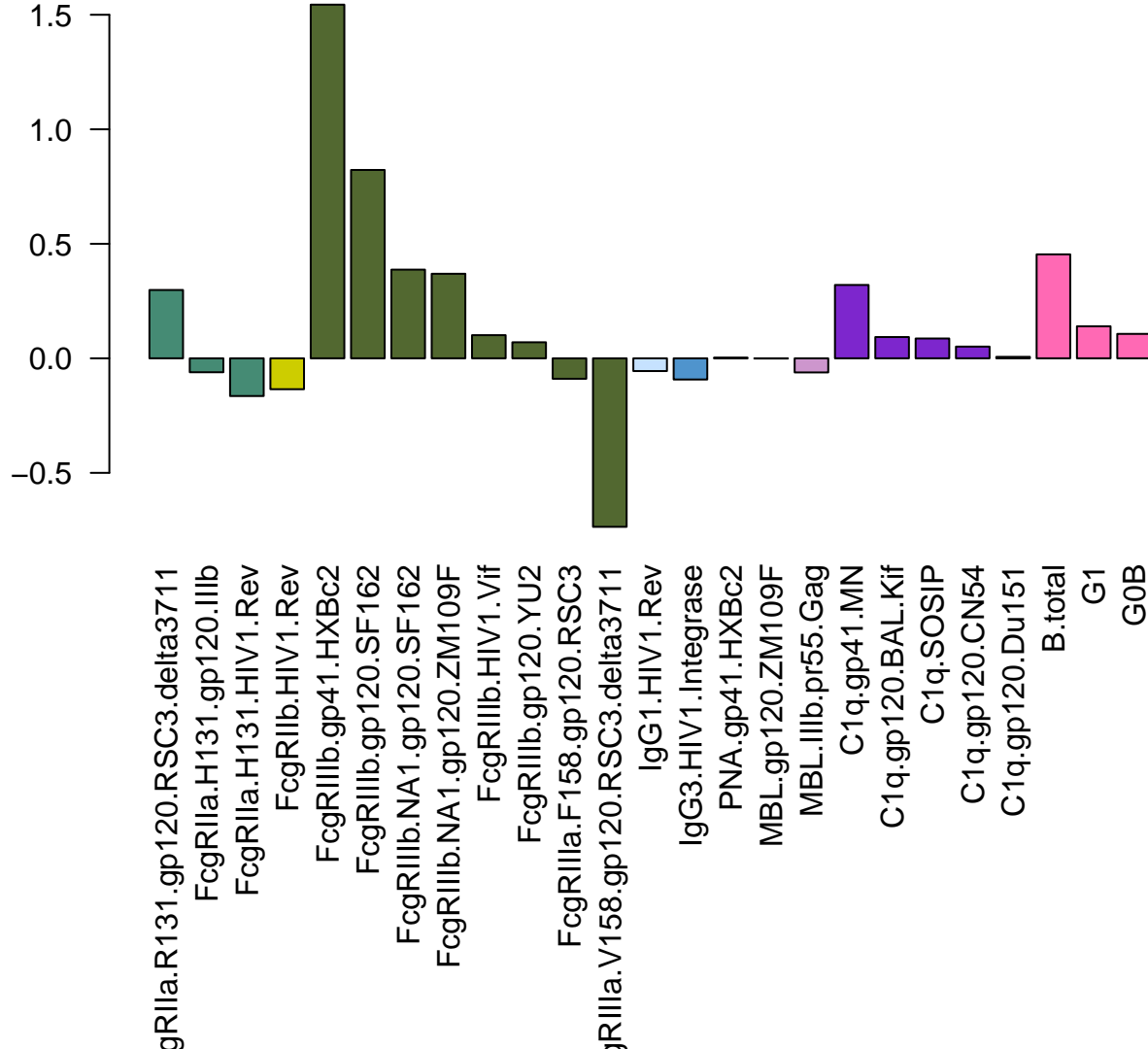

Supplement: Supplementary file 5 — Dataset EV3 [file MSB-14-e7881-s005.zip › dataset_EV3/Fc.array.plus.glycan/ADCC/lambda.min/fullmod-coeffs.pdf]

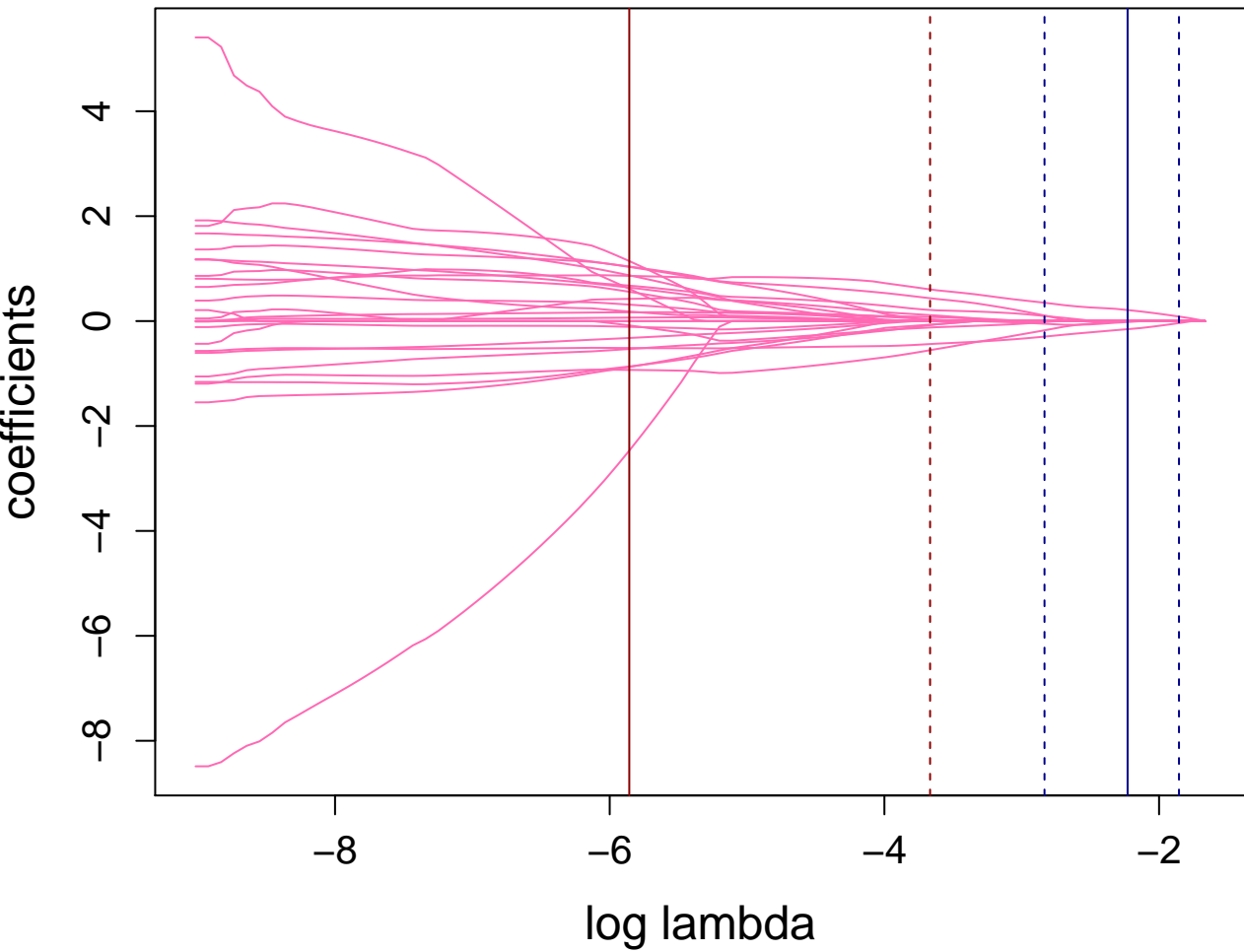

Supplement: Supplementary file 5 — Dataset EV3 [file MSB-14-e7881-s005.zip › dataset_EV3/glycan/class.nv/coeff-path.pdf]

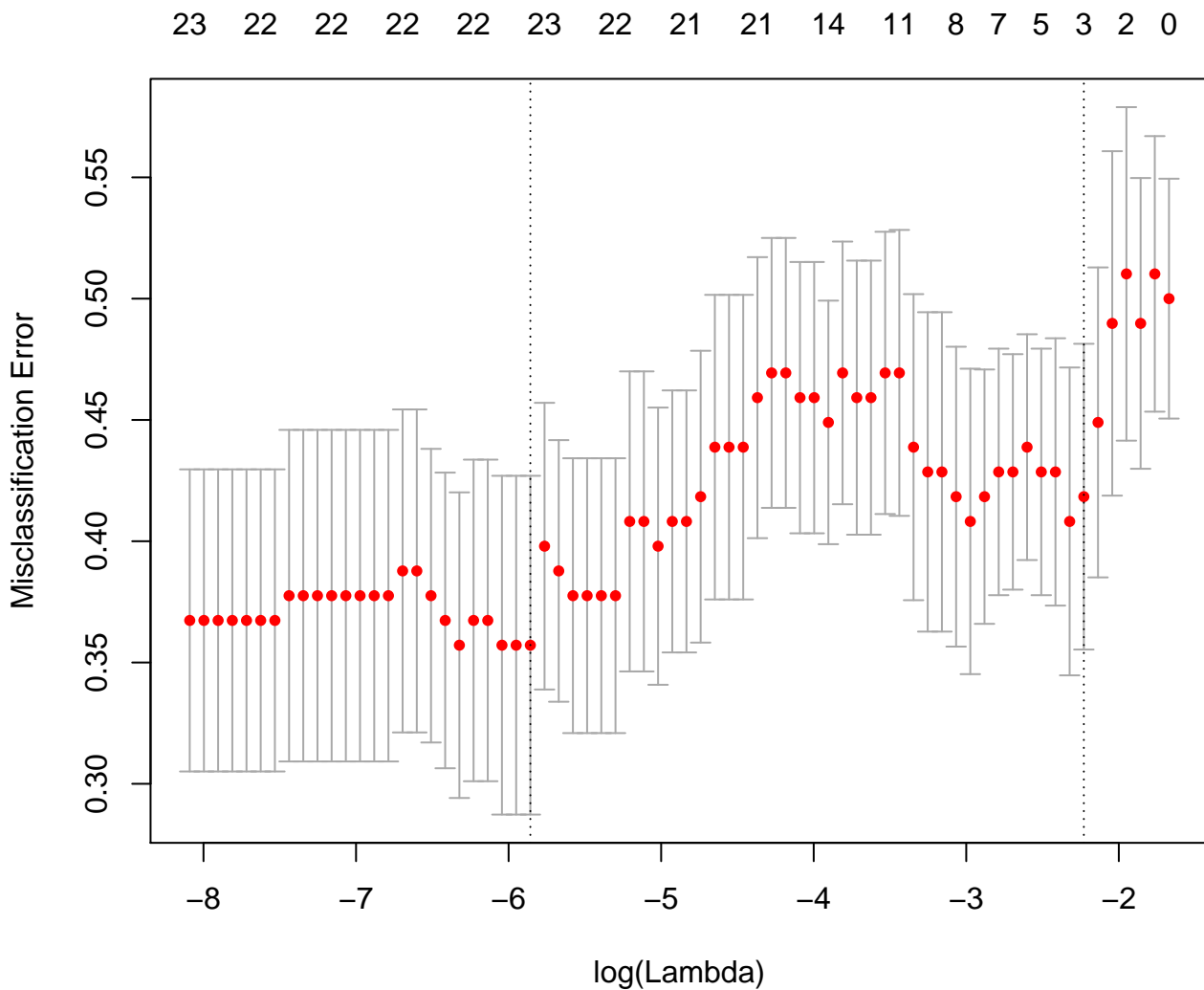

Supplement: Supplementary file 5 — Dataset EV3 [file MSB-14-e7881-s005.zip › dataset_EV3/glycan/class.nv/lambda.min/cv-results.pdf]

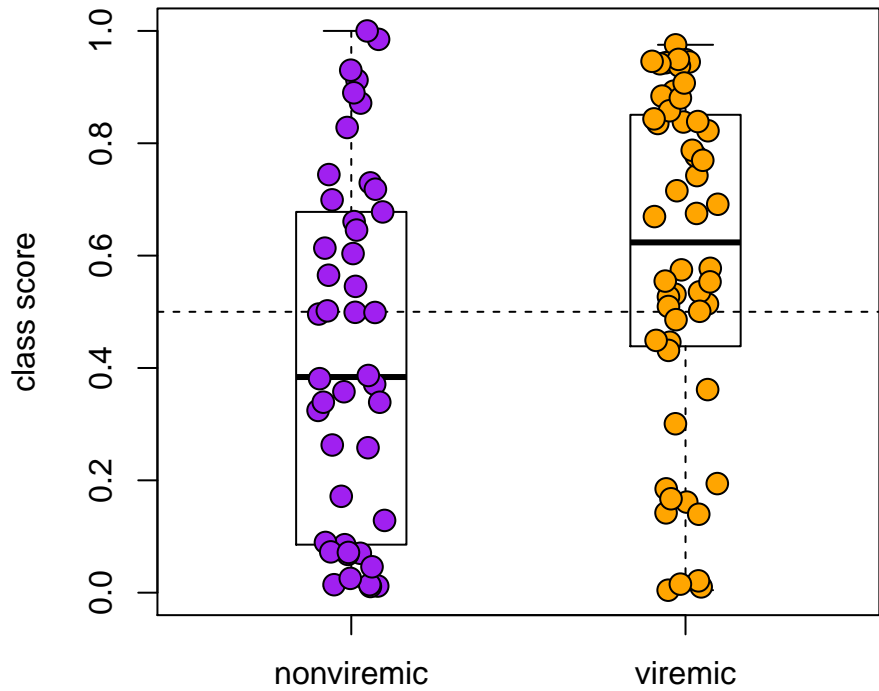

Supplement: Supplementary file 5 — Dataset EV3 [file MSB-14-e7881-s005.zip › dataset_EV3/glycan/class.nv/lambda.min/cvmod-box.pdf]

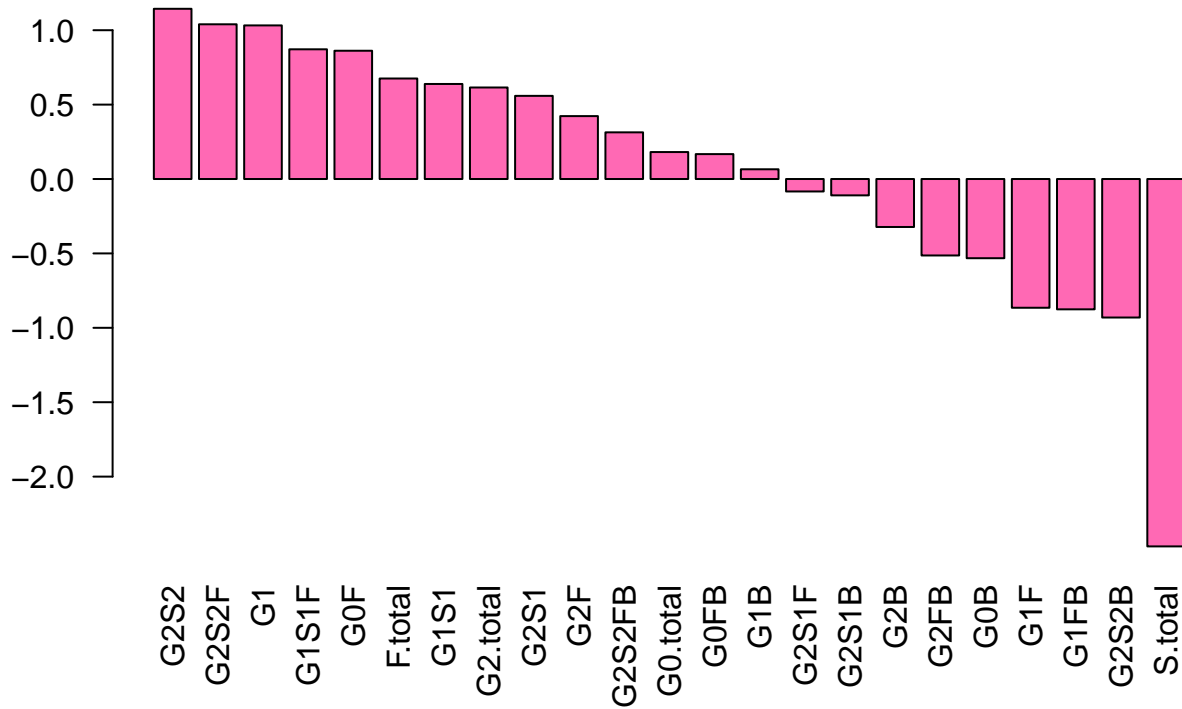

Supplement: Supplementary file 5 — Dataset EV3 [file MSB-14-e7881-s005.zip › dataset_EV3/glycan/class.nv/lambda.min/fullmod-coeffs.pdf]

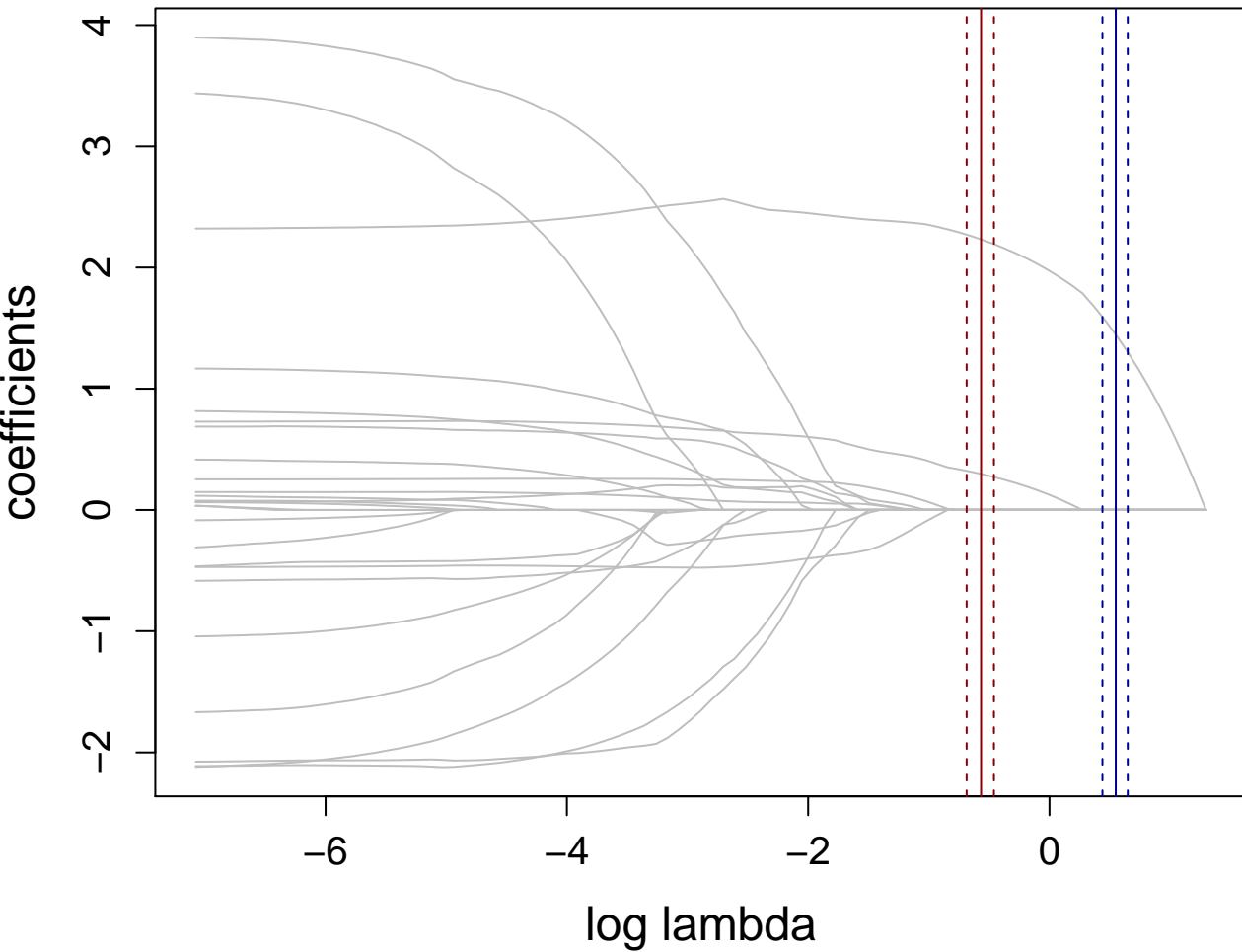

Supplement: Supplementary file 5 — Dataset EV3 [file MSB-14-e7881-s005.zip › dataset_EV3/titer/ADCC/coeff-path.pdf]

Mean-Squared Error

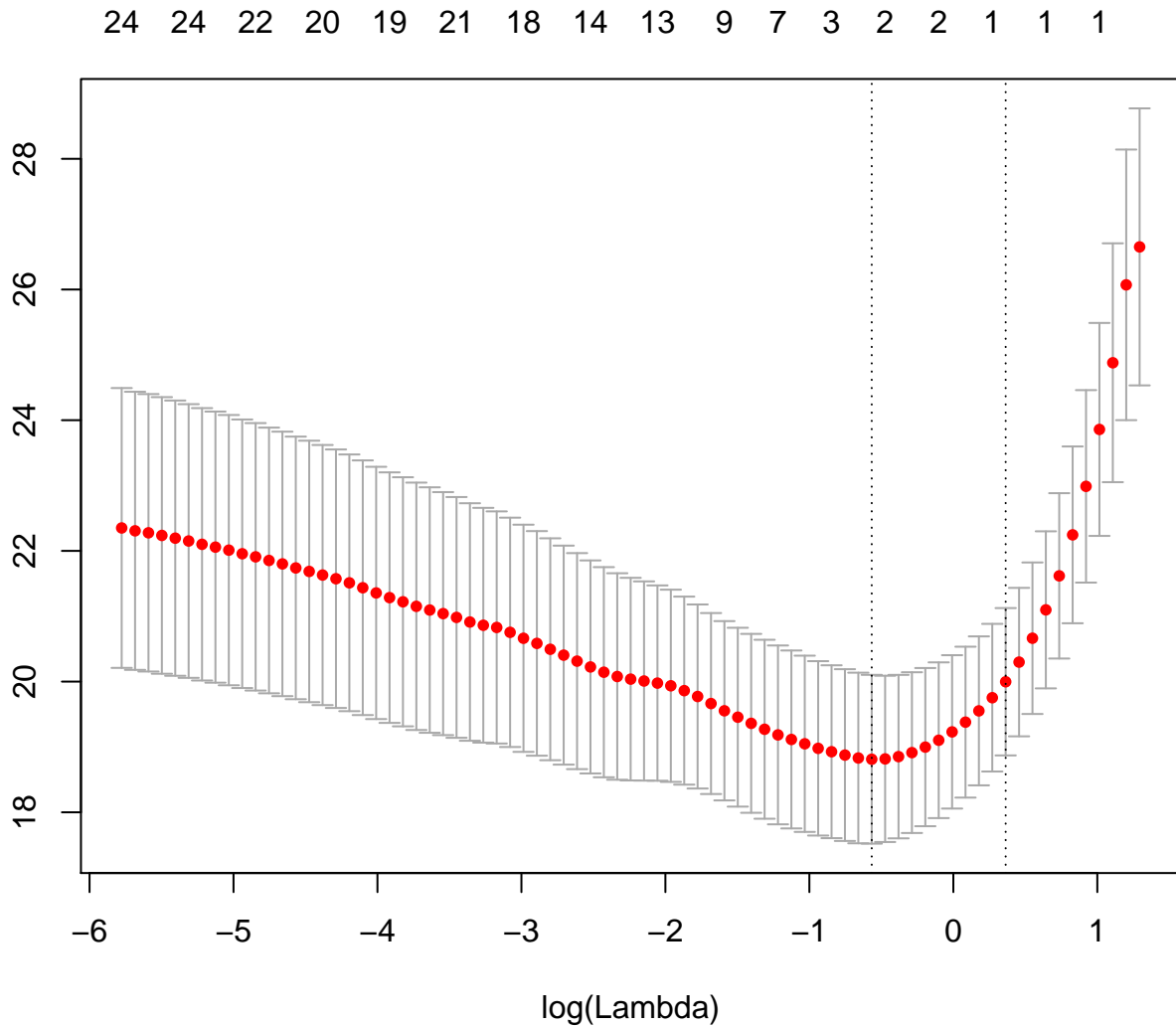

Supplement: Supplementary file 5 — Dataset EV3 [file MSB-14-e7881-s005.zip › dataset_EV3/titer/ADCC/lambda.min/cv-results.pdf]

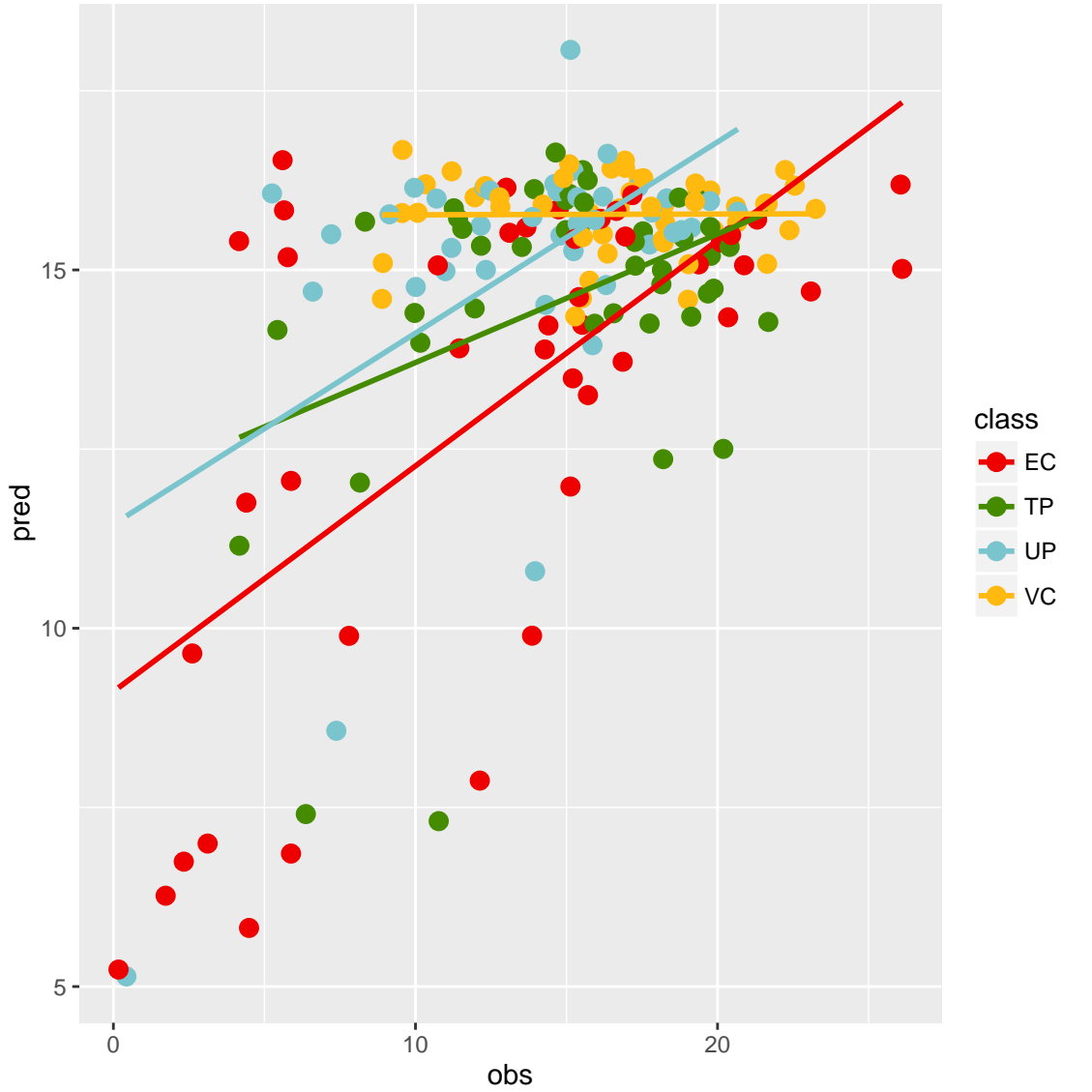

Supplement: Supplementary file 5 — Dataset EV3 [file MSB-14-e7881-s005.zip › dataset_EV3/titer/ADCC/lambda.min/cvmod-scatter.pdf]

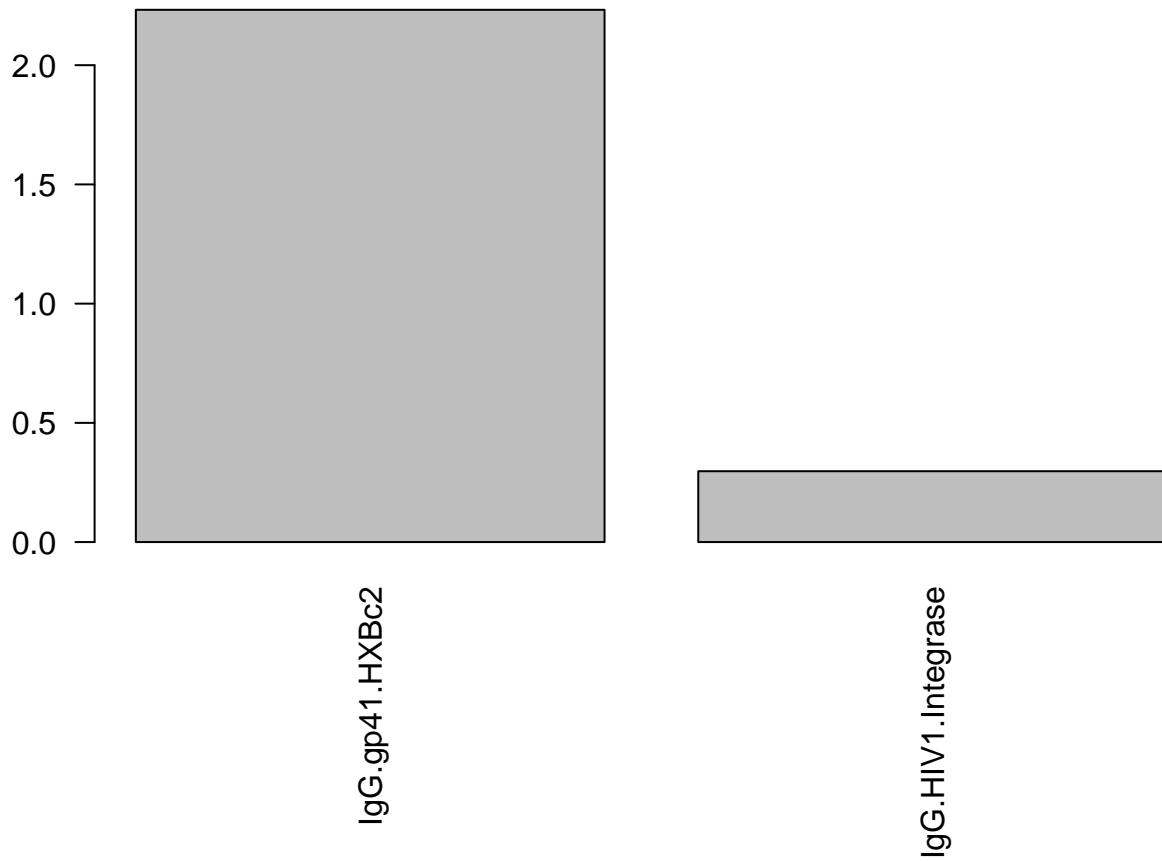

Supplement: Supplementary file 5 — Dataset EV3 [file MSB-14-e7881-s005.zip › dataset_EV3/titer/ADCC/lambda.min/fullmod-coeffs.pdf]

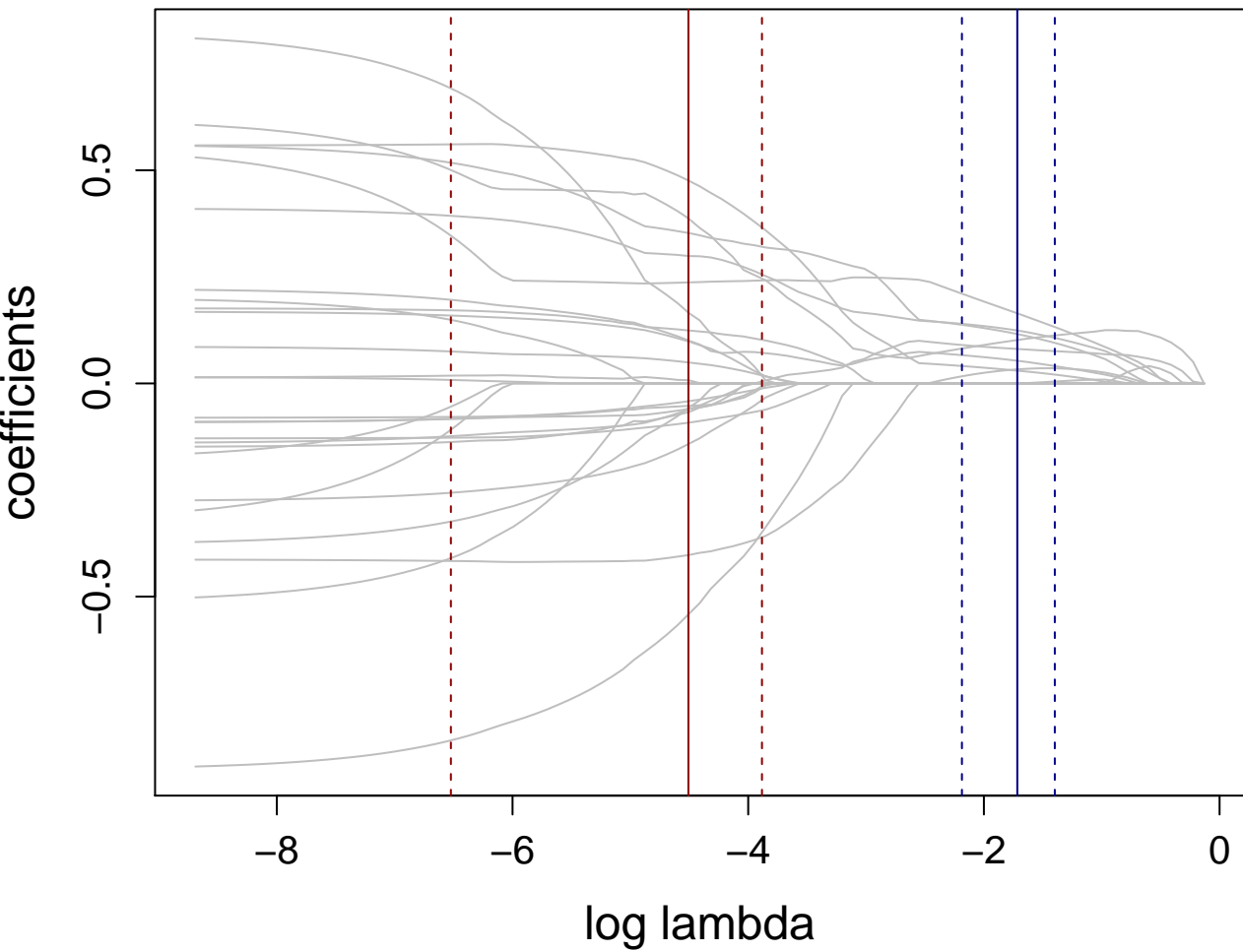

Supplement: Supplementary file 5 — Dataset EV3 [file MSB-14-e7881-s005.zip › dataset_EV3/titer/ADCD/coeff-path.pdf]

Mean-Squared Error

26 26 26 26 26 23 23 21 18 11 8 8 8 8 9 9 5 0

1.2  
1.0  
0.8  
0.6

-8 -6 -4 -2 0

$\log(\text{Lambda})$

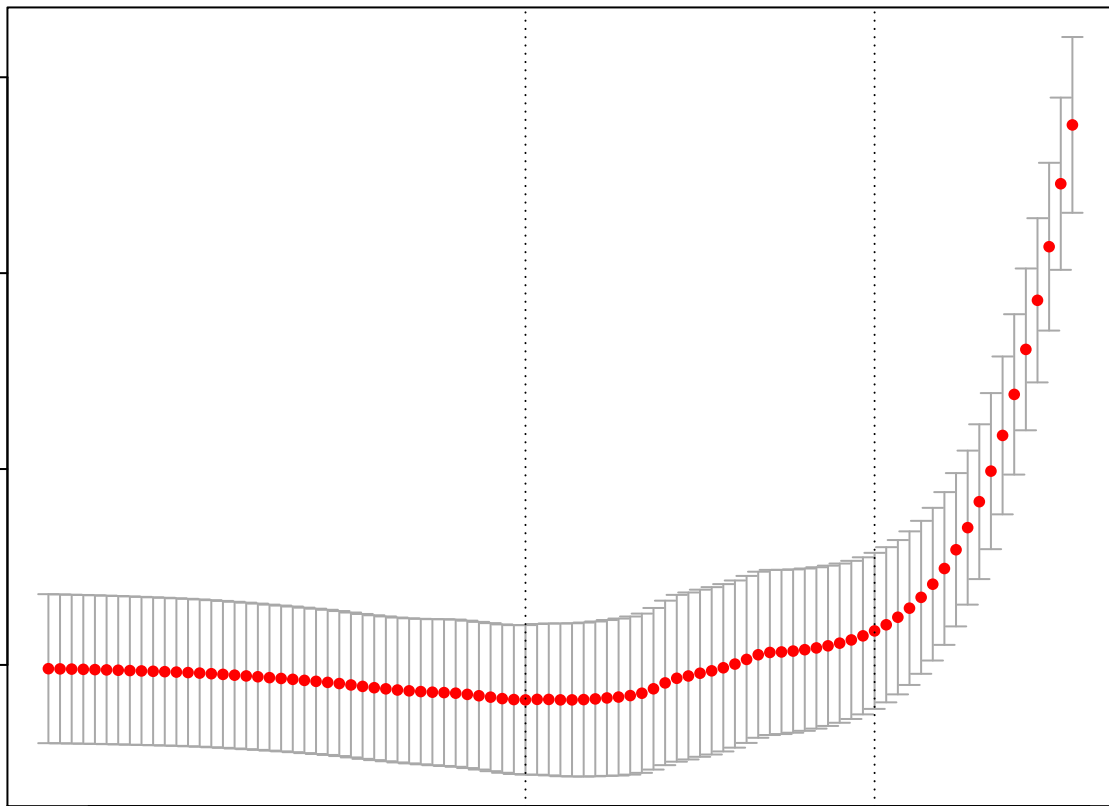

Supplement: Supplementary file 5 — Dataset EV3 [file MSB-14-e7881-s005.zip › dataset_EV3/titer/ADCD/lambda.min/cv-results.pdf]

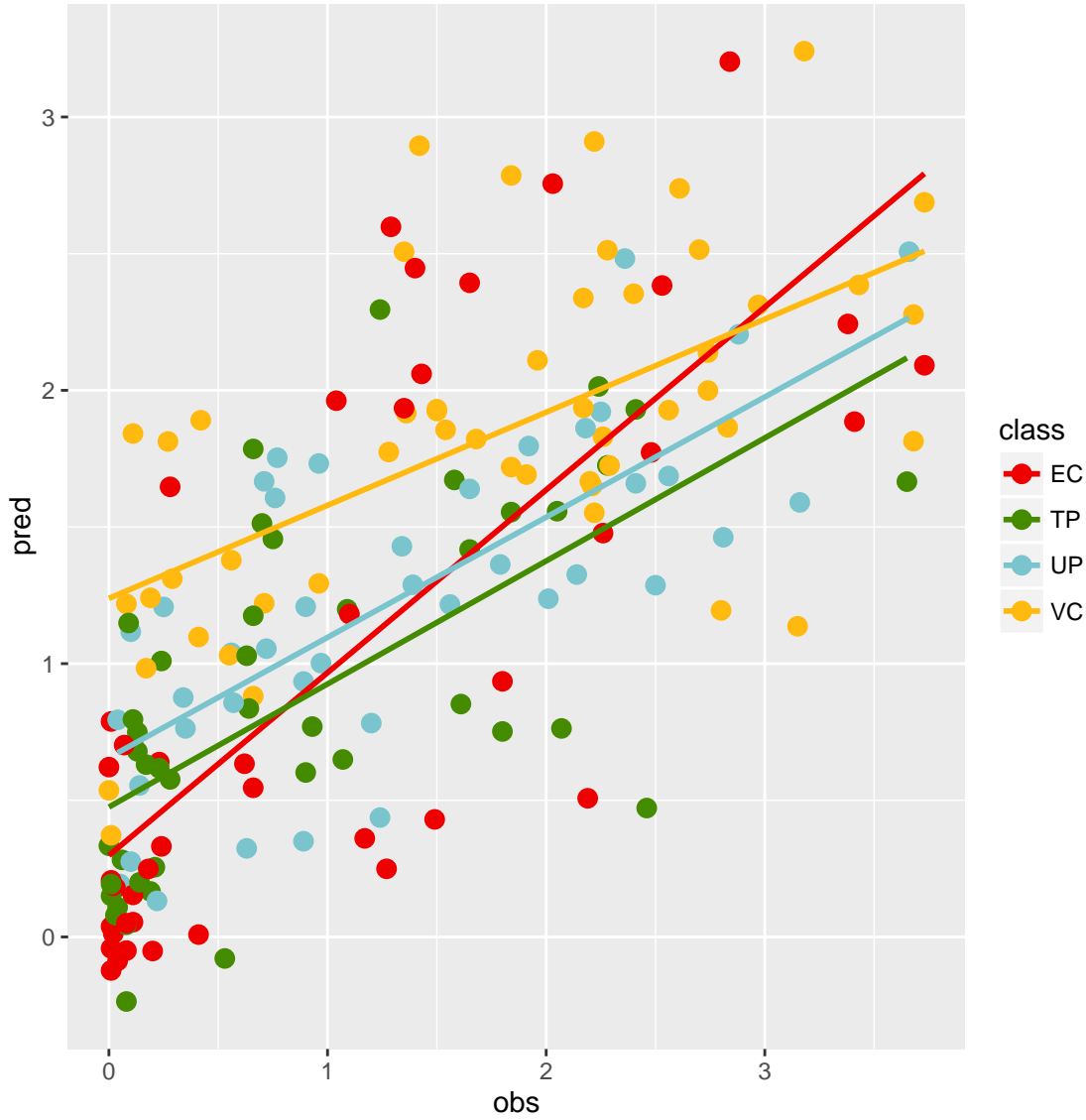

Supplement: Supplementary file 5 — Dataset EV3 [file MSB-14-e7881-s005.zip › dataset_EV3/titer/ADCD/lambda.min/cvmod-scatter.pdf]

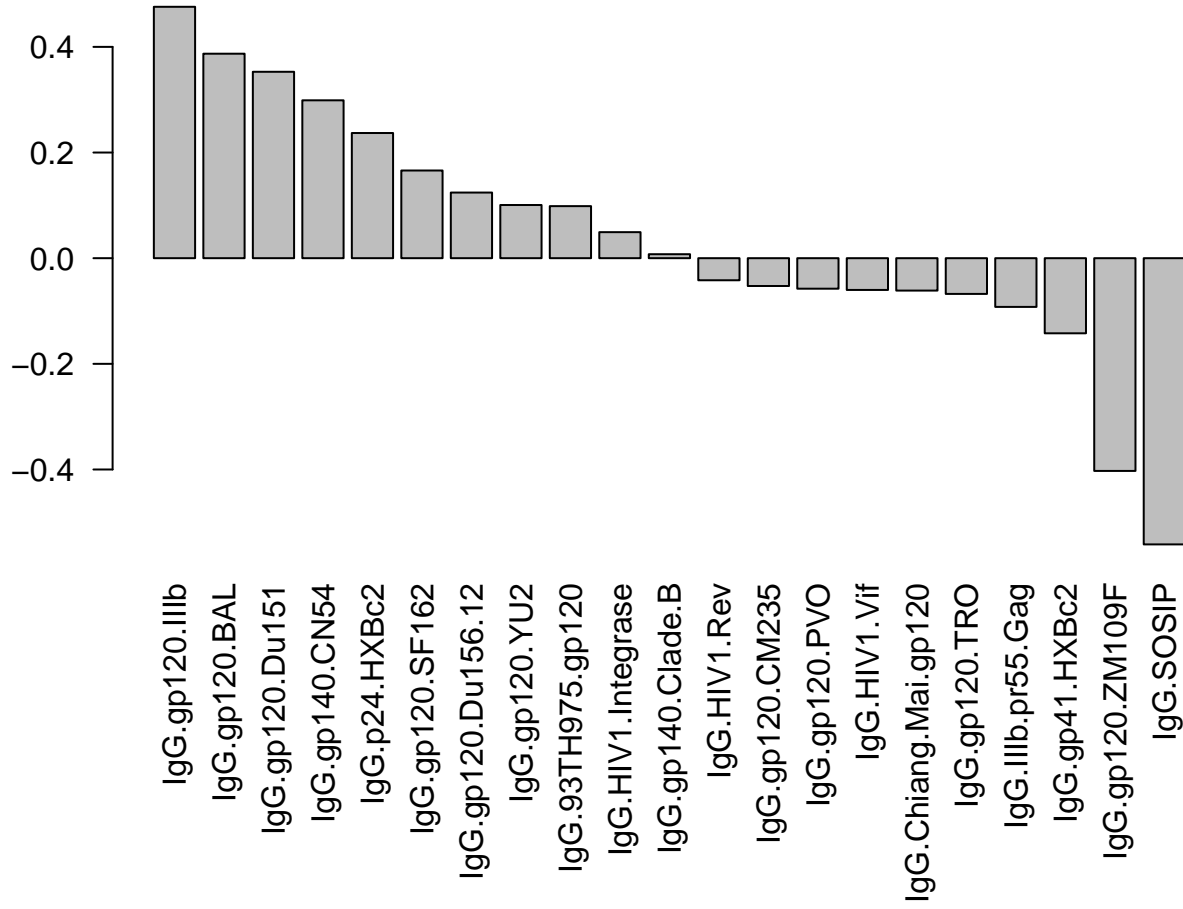

Supplement: Supplementary file 5 — Dataset EV3 [file MSB-14-e7881-s005.zip › dataset_EV3/titer/ADCD/lambda.min/fullmod-coeffs.pdf]

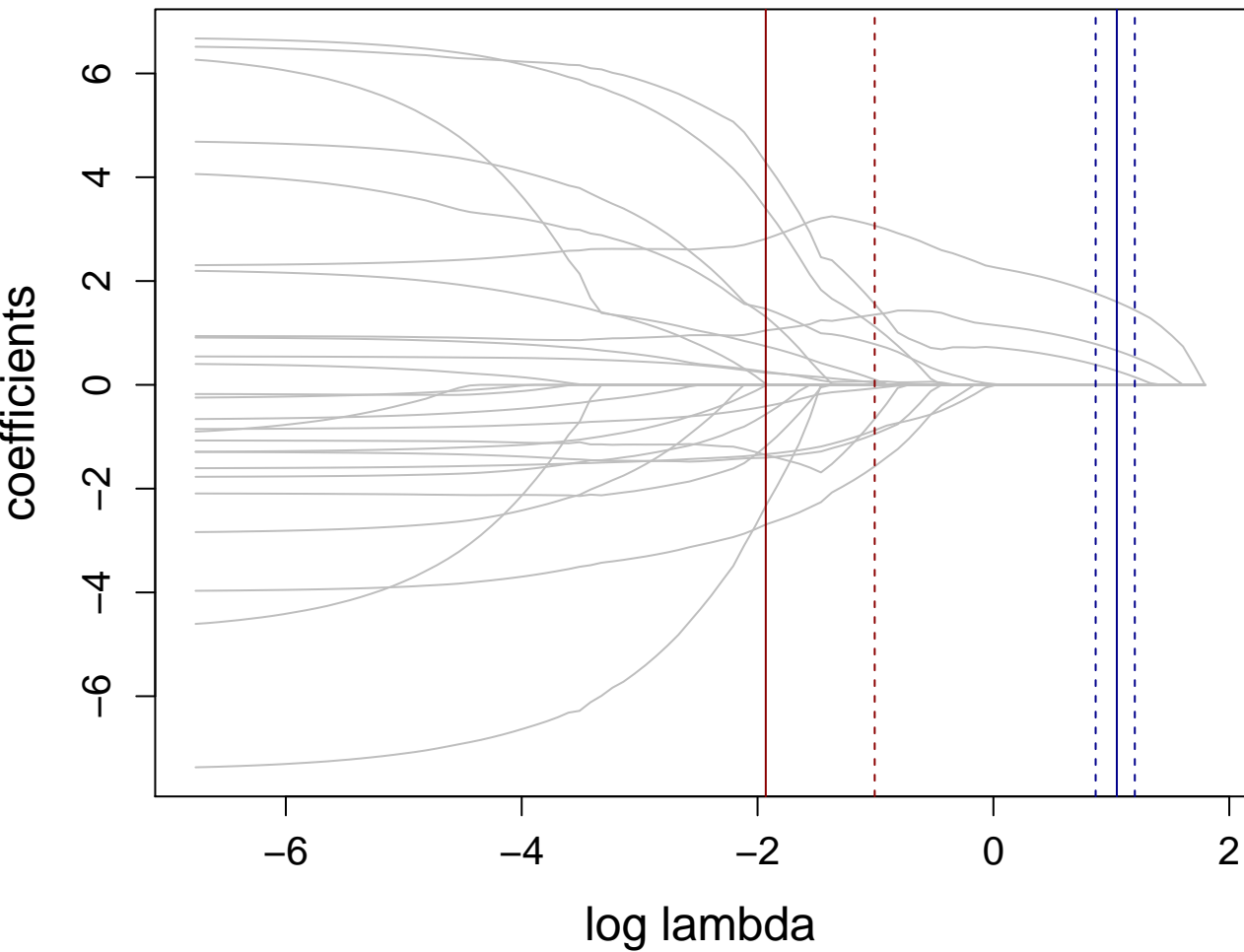

Supplement: Supplementary file 5 — Dataset EV3 [file MSB-14-e7881-s005.zip › dataset_EV3/titer/ADNP/coeff-path.pdf]

Mean-Squared Error

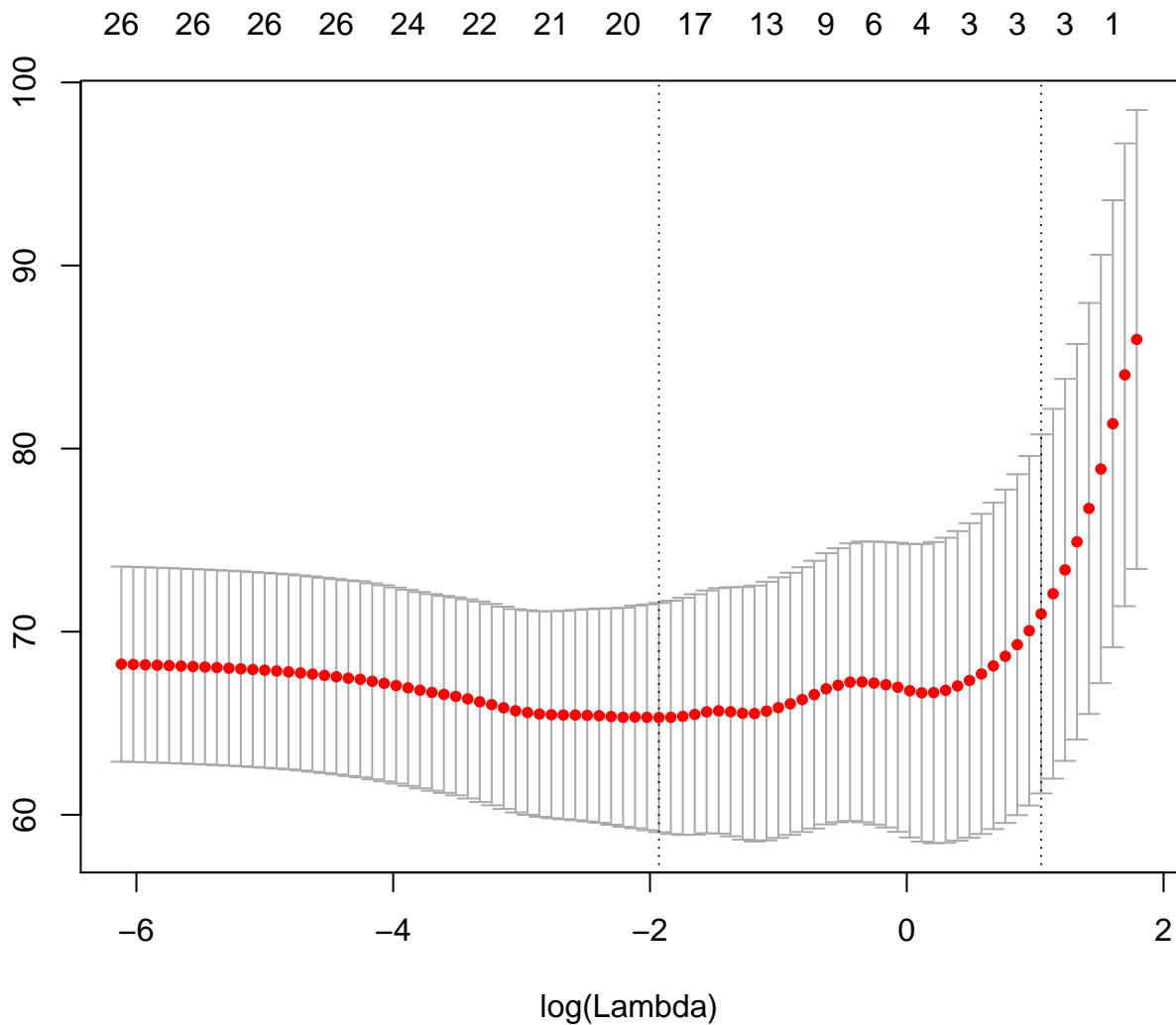

Supplement: Supplementary file 5 — Dataset EV3 [file MSB-14-e7881-s005.zip › dataset_EV3/titer/ADNP/lambda.min/cv-results.pdf]

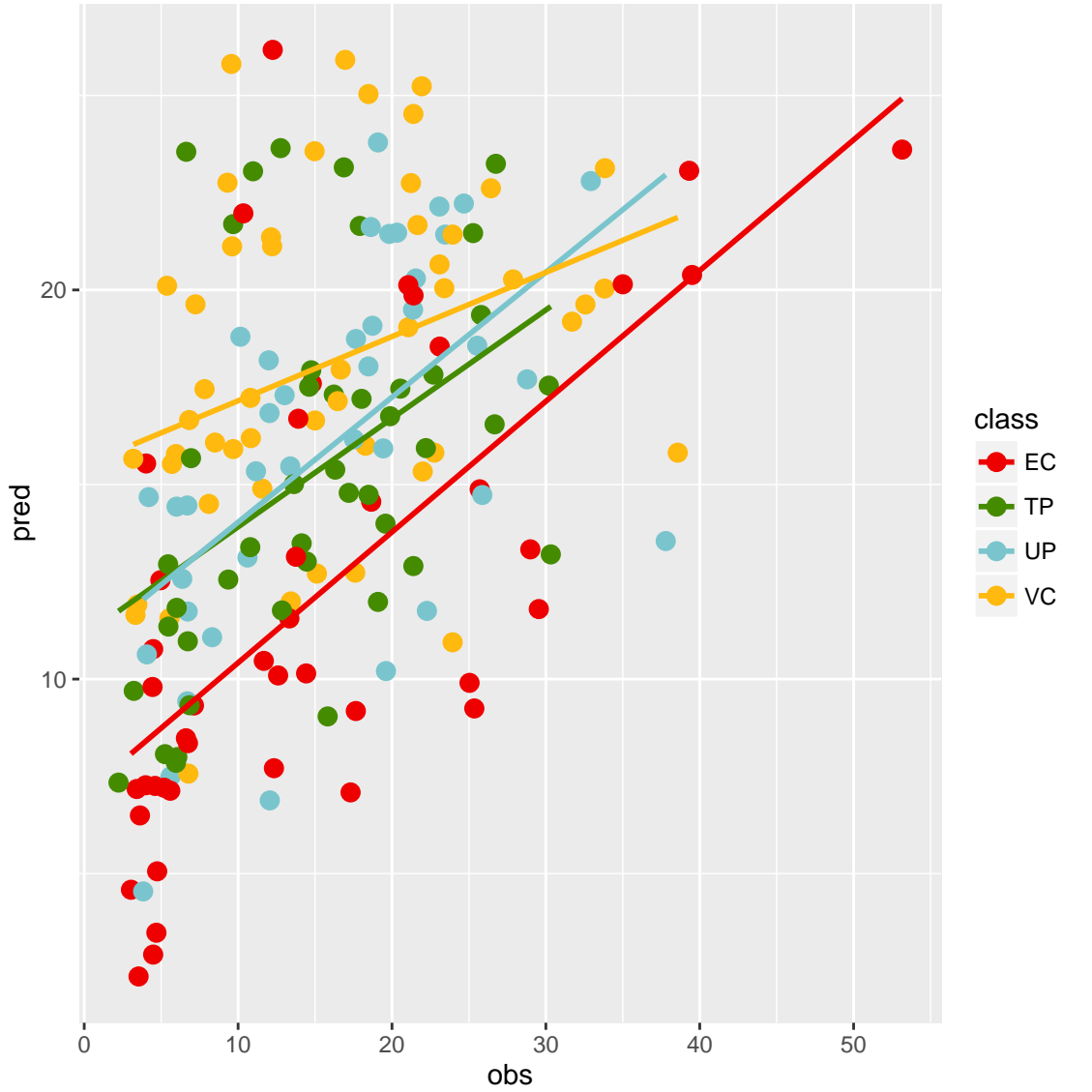

Supplement: Supplementary file 5 — Dataset EV3 [file MSB-14-e7881-s005.zip › dataset_EV3/titer/ADNP/lambda.min/cvmod-scatter.pdf]

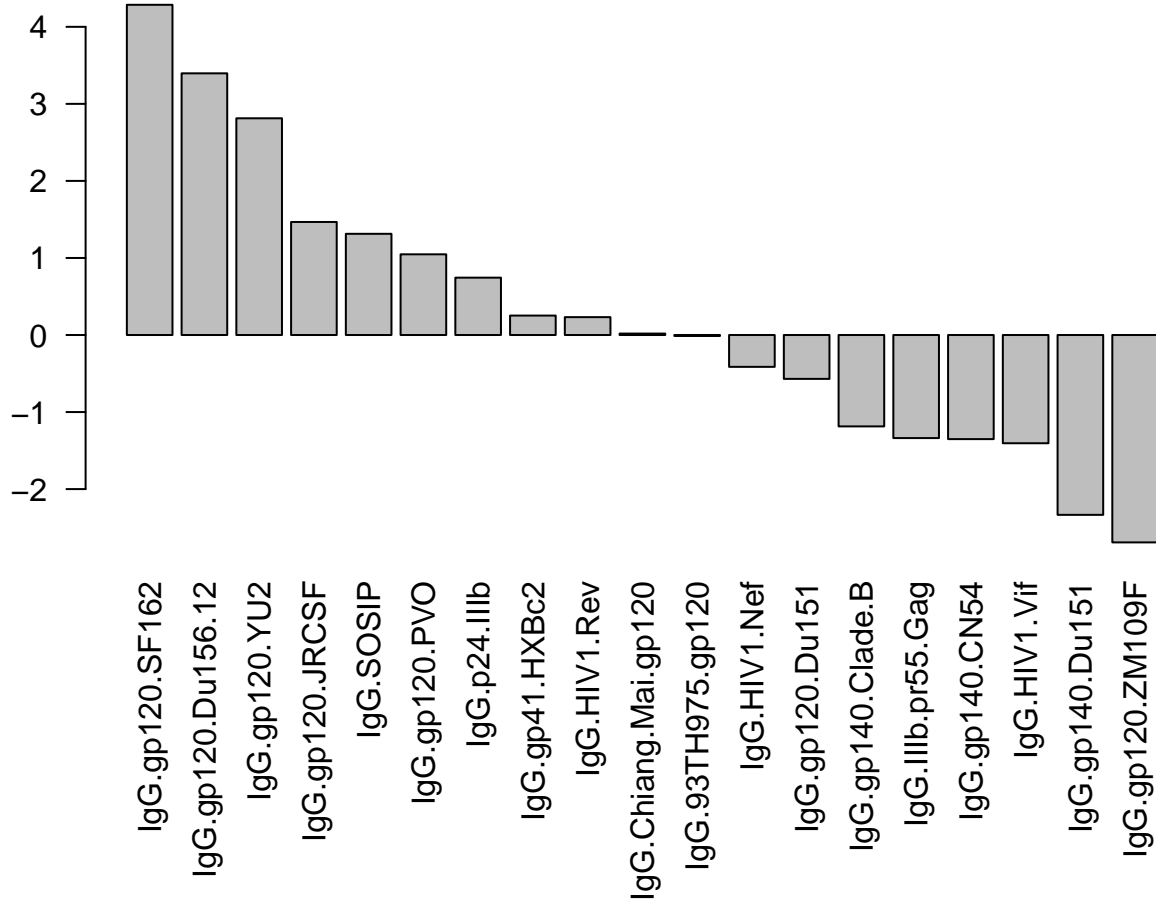

Supplement: Supplementary file 5 — Dataset EV3 [file MSB-14-e7881-s005.zip › dataset_EV3/titer/ADNP/lambda.min/fullmod-coeffs.pdf]

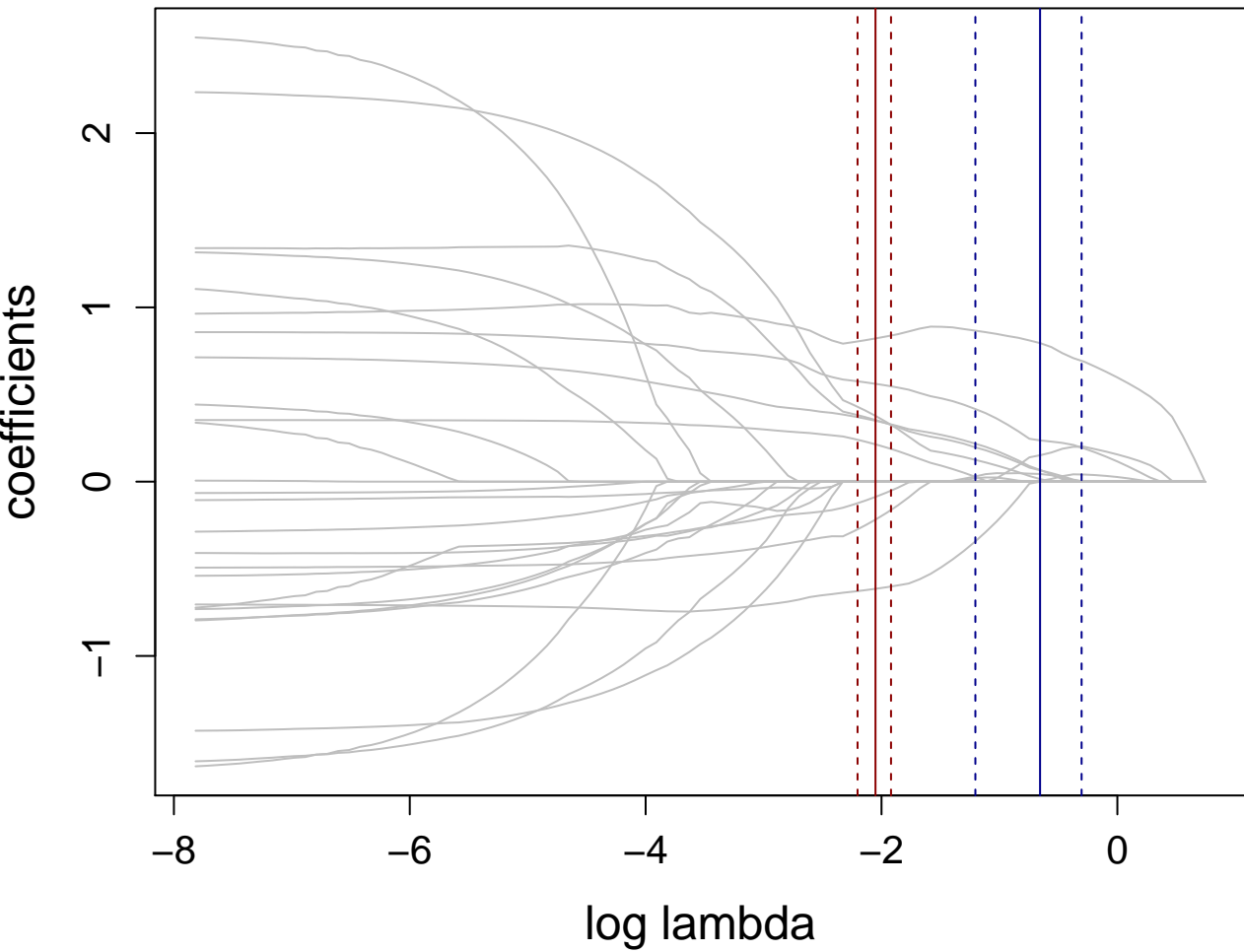

Supplement: Supplementary file 5 — Dataset EV3 [file MSB-14-e7881-s005.zip › dataset_EV3/titer/CD107a/coeff-path.pdf]

Mean-Squared Error

26 25 25 24 24 23 22 19 17 14 9 9 9 9 8 4 4 2

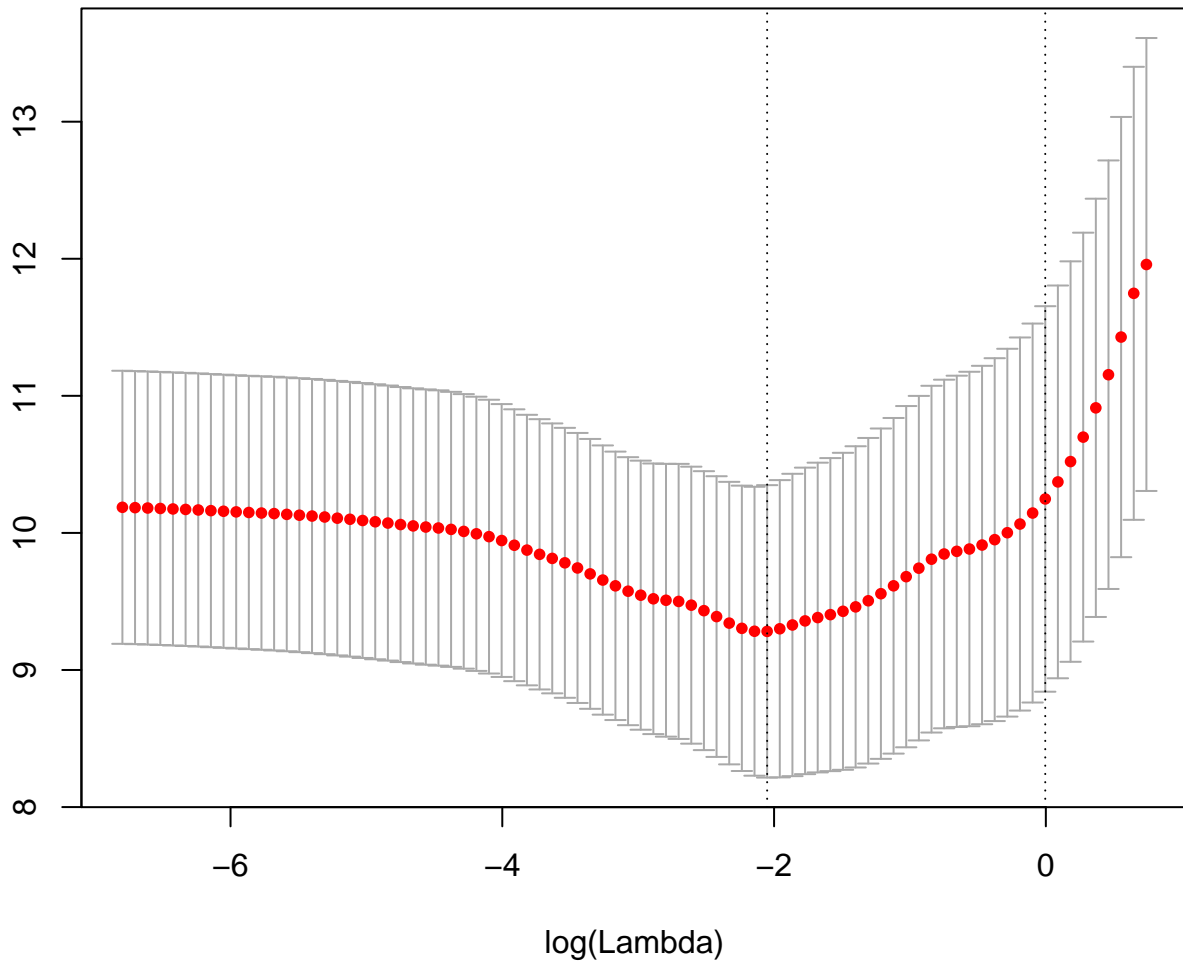

Supplement: Supplementary file 5 — Dataset EV3 [file MSB-14-e7881-s005.zip › dataset_EV3/titer/CD107a/lambda.min/cv-results.pdf]

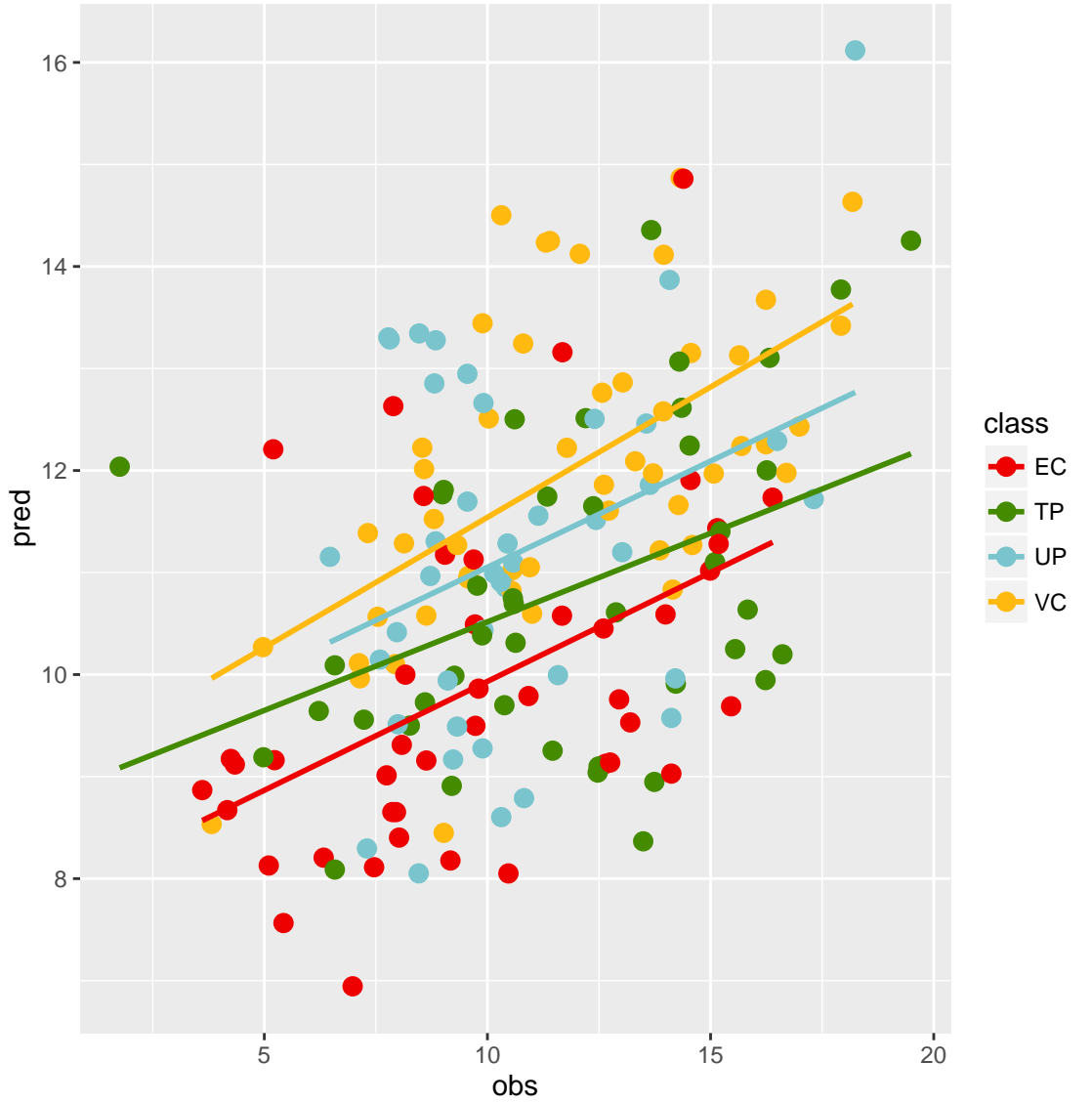

Supplement: Supplementary file 5 — Dataset EV3 [file MSB-14-e7881-s005.zip › dataset_EV3/titer/CD107a/lambda.min/cvmod-scatter.pdf]

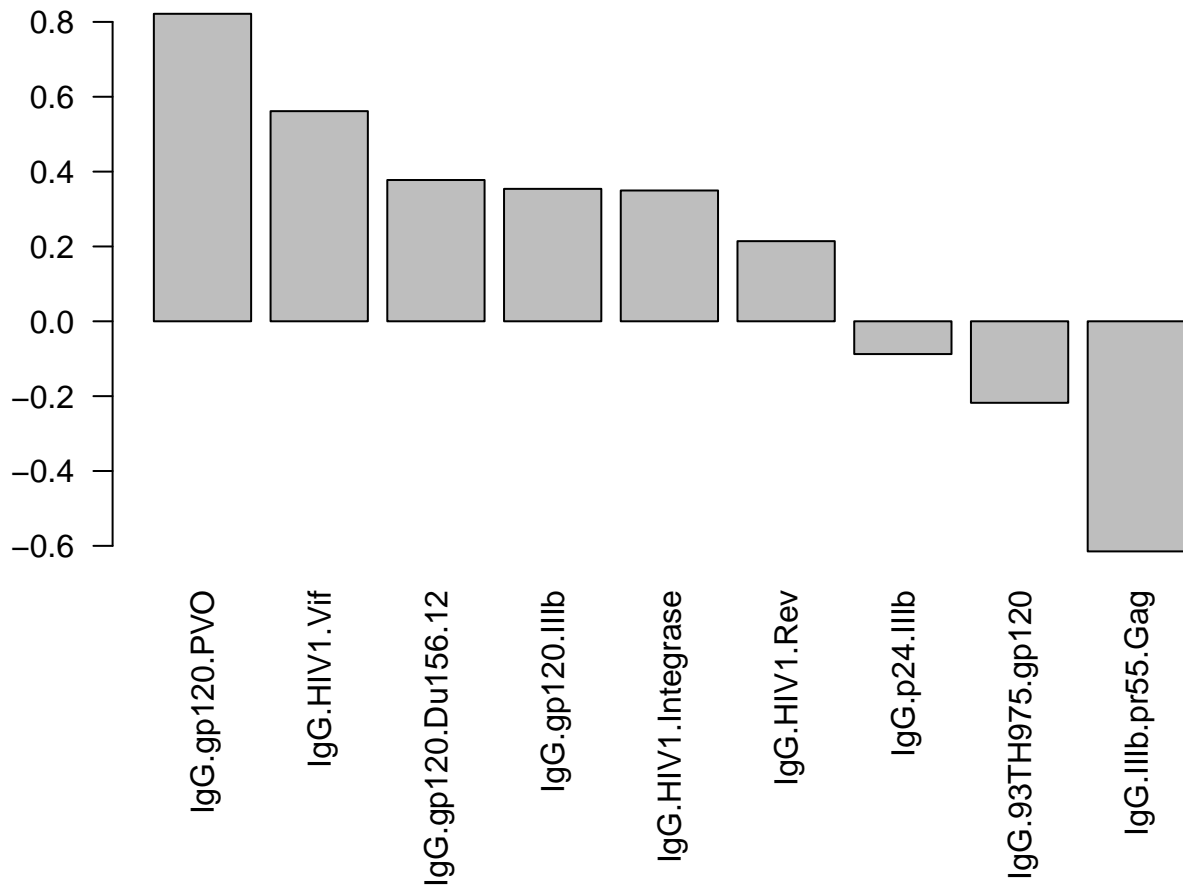

Supplement: Supplementary file 5 — Dataset EV3 [file MSB-14-e7881-s005.zip › dataset_EV3/titer/CD107a/lambda.min/fullmod-coeffs.pdf]

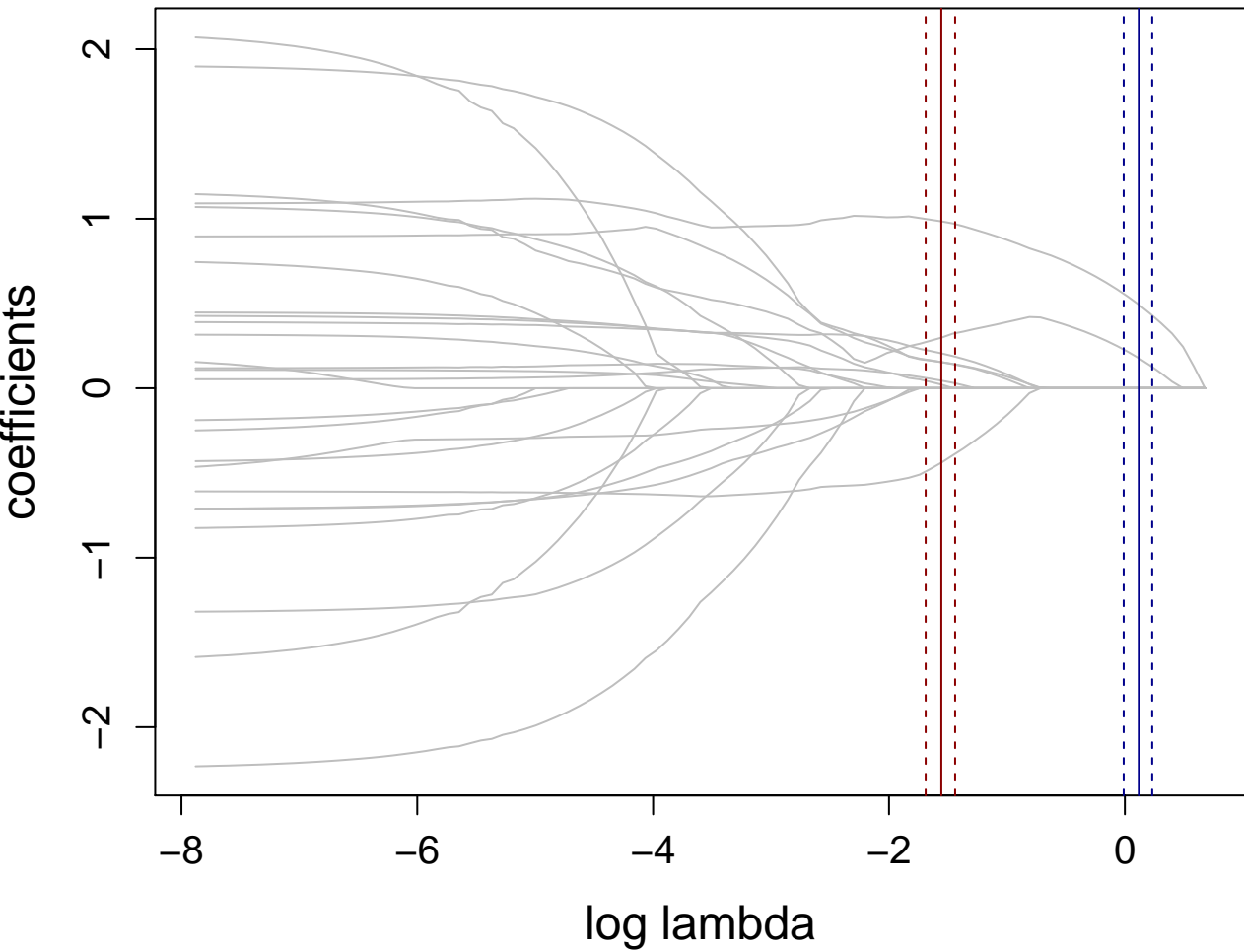

Supplement: Supplementary file 5 — Dataset EV3 [file MSB-14-e7881-s005.zip › dataset_EV3/titer/IFNy/coeff-path.pdf]

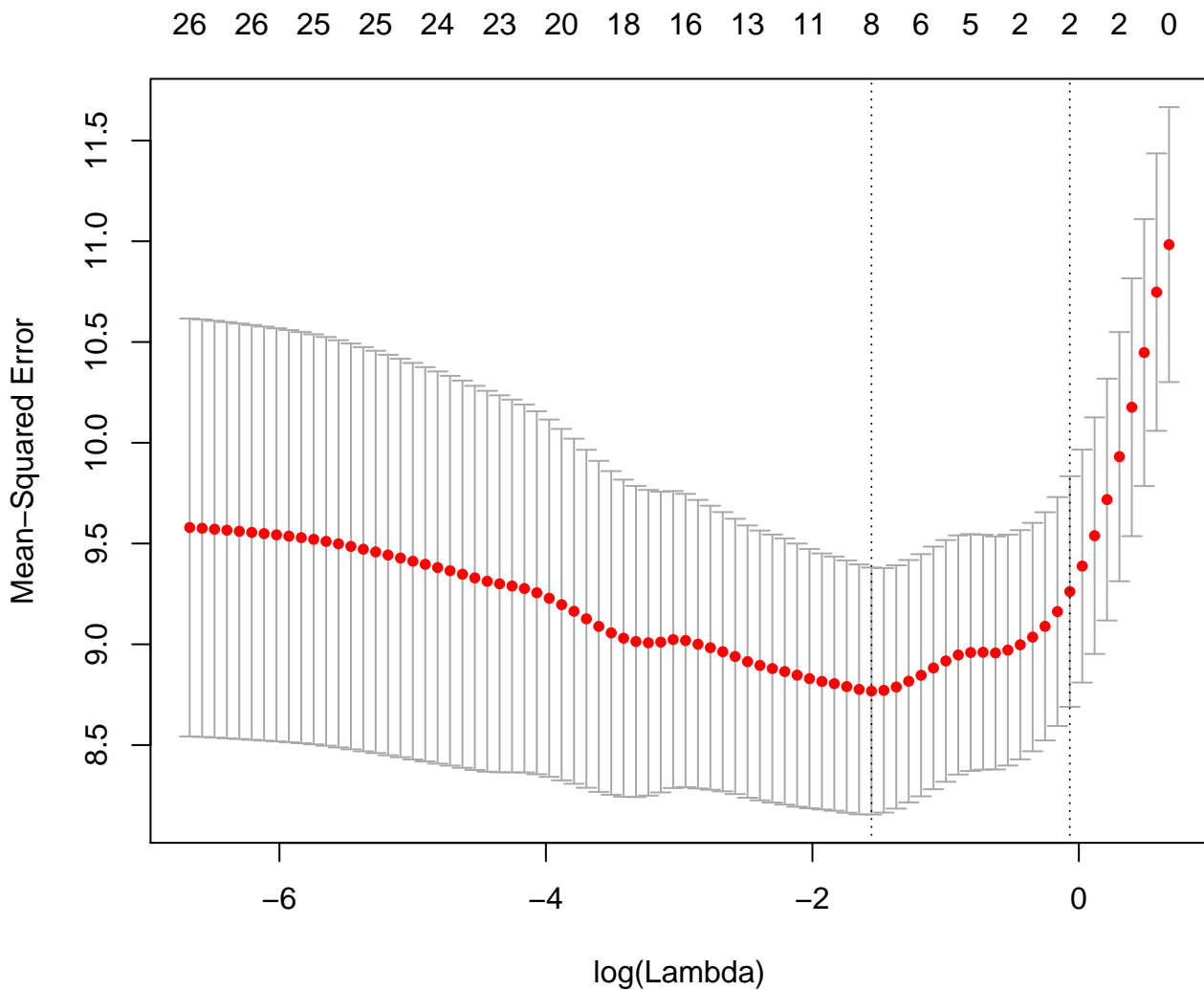

Supplement: Supplementary file 5 — Dataset EV3 [file MSB-14-e7881-s005.zip › dataset_EV3/titer/IFNy/lambda.min/cv-results.pdf]

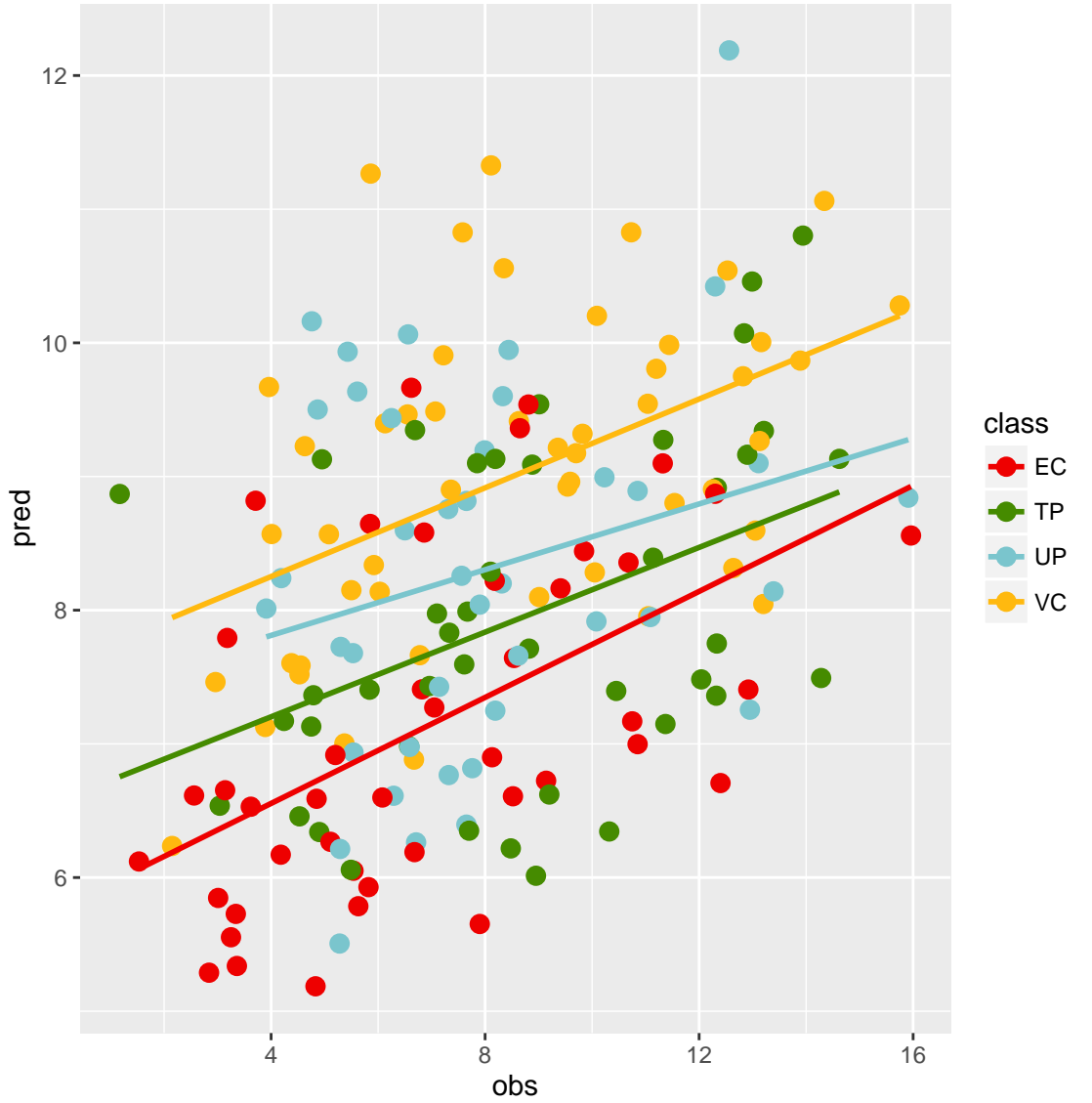

Supplement: Supplementary file 5 — Dataset EV3 [file MSB-14-e7881-s005.zip › dataset_EV3/titer/IFNy/lambda.min/cvmod-scatter.pdf]

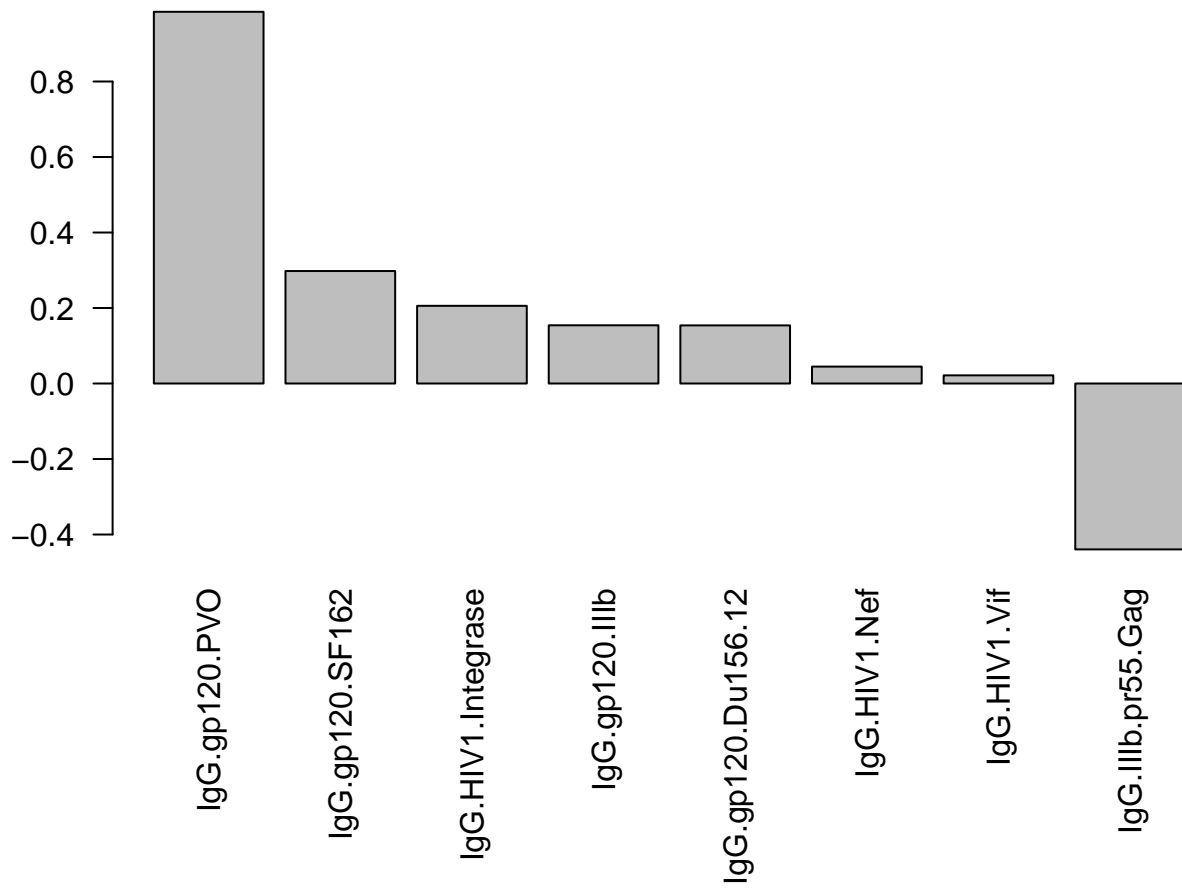

Supplement: Supplementary file 5 — Dataset EV3 [file MSB-14-e7881-s005.zip › dataset_EV3/titer/IFNy/lambda.min/fullmod-coeffs.pdf]

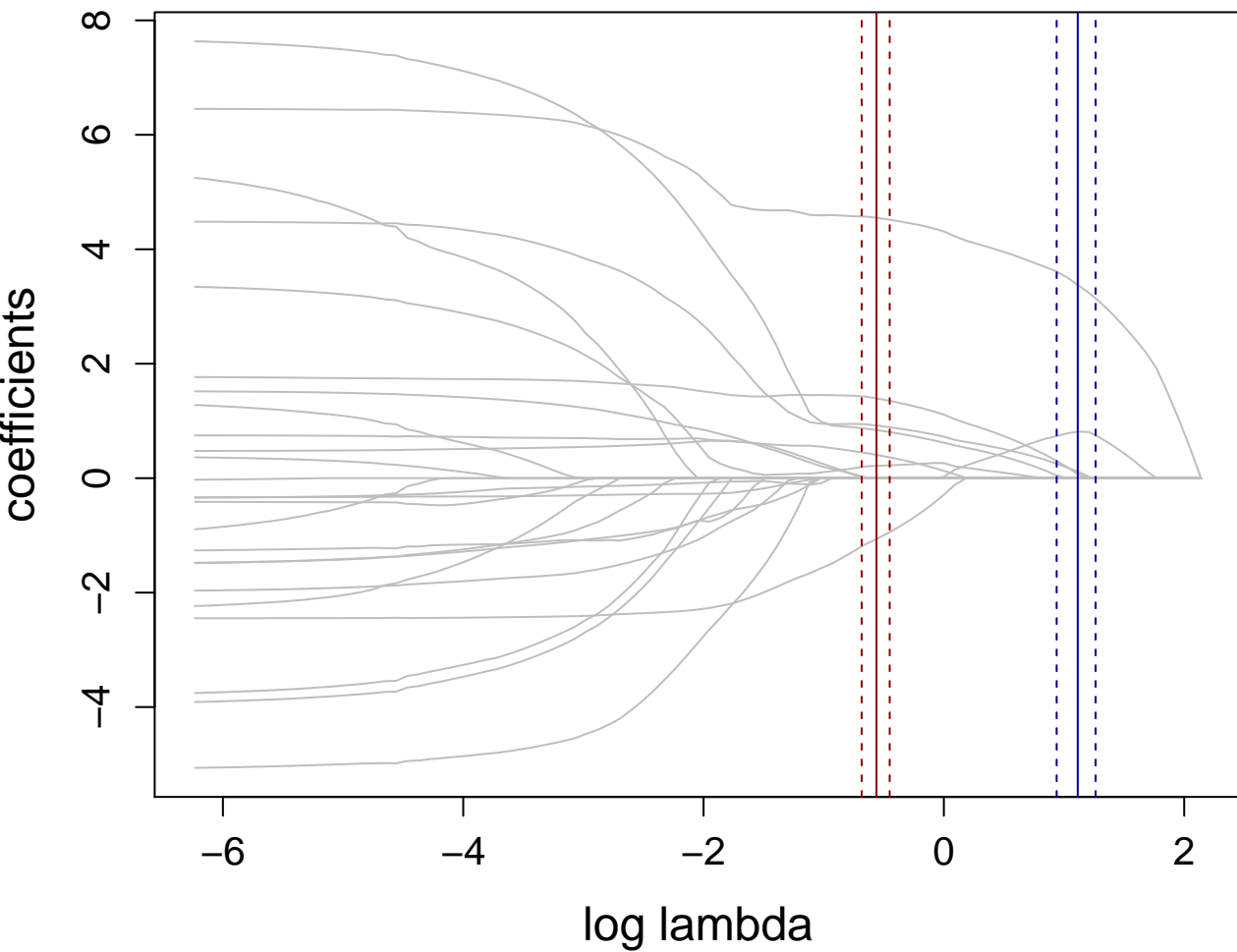

Supplement: Supplementary file 5 — Dataset EV3 [file MSB-14-e7881-s005.zip › dataset_EV3/titer/MIP1b/coeff-path.pdf]

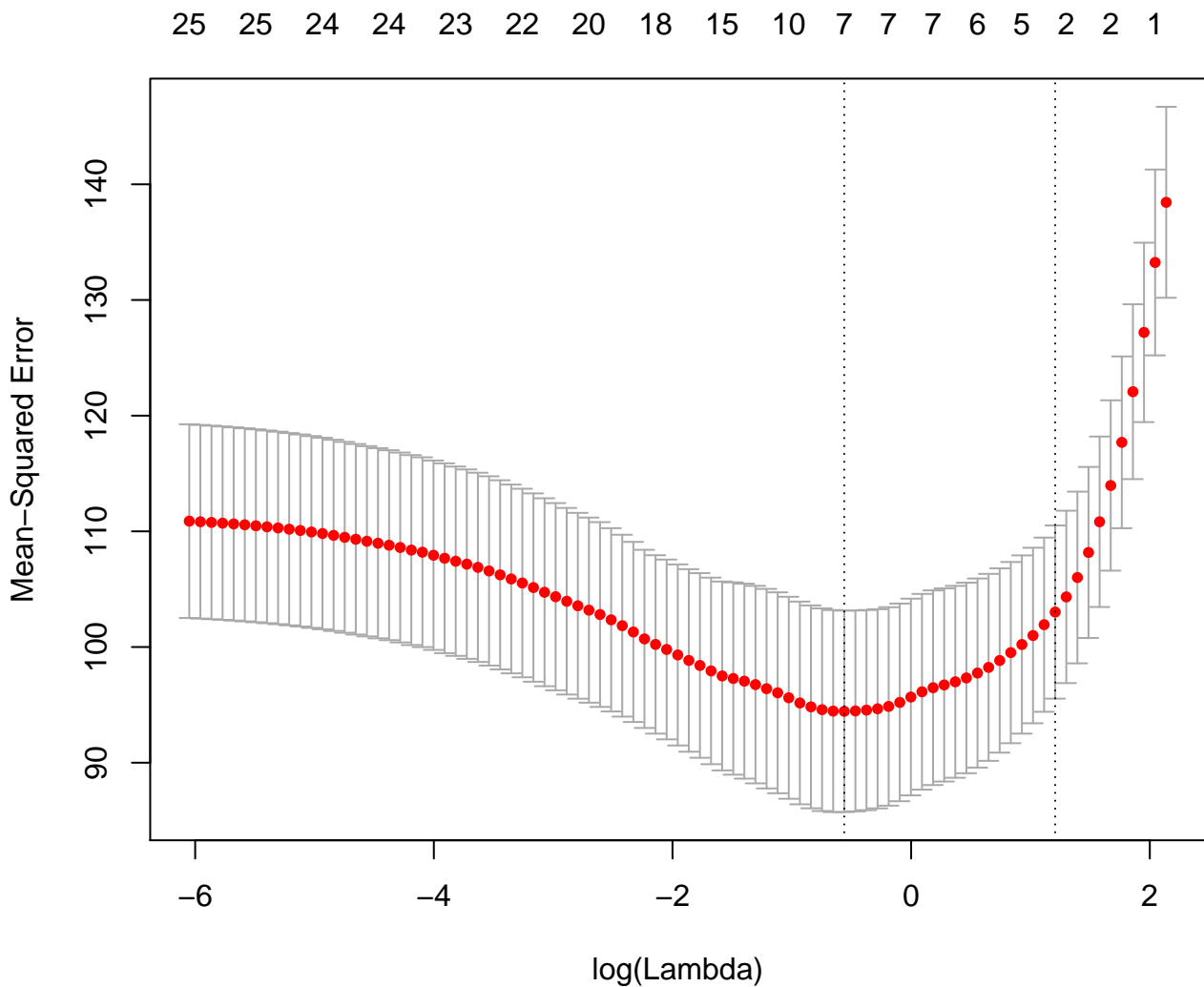

Supplement: Supplementary file 5 — Dataset EV3 [file MSB-14-e7881-s005.zip › dataset_EV3/titer/MIP1b/lambda.min/cv-results.pdf]

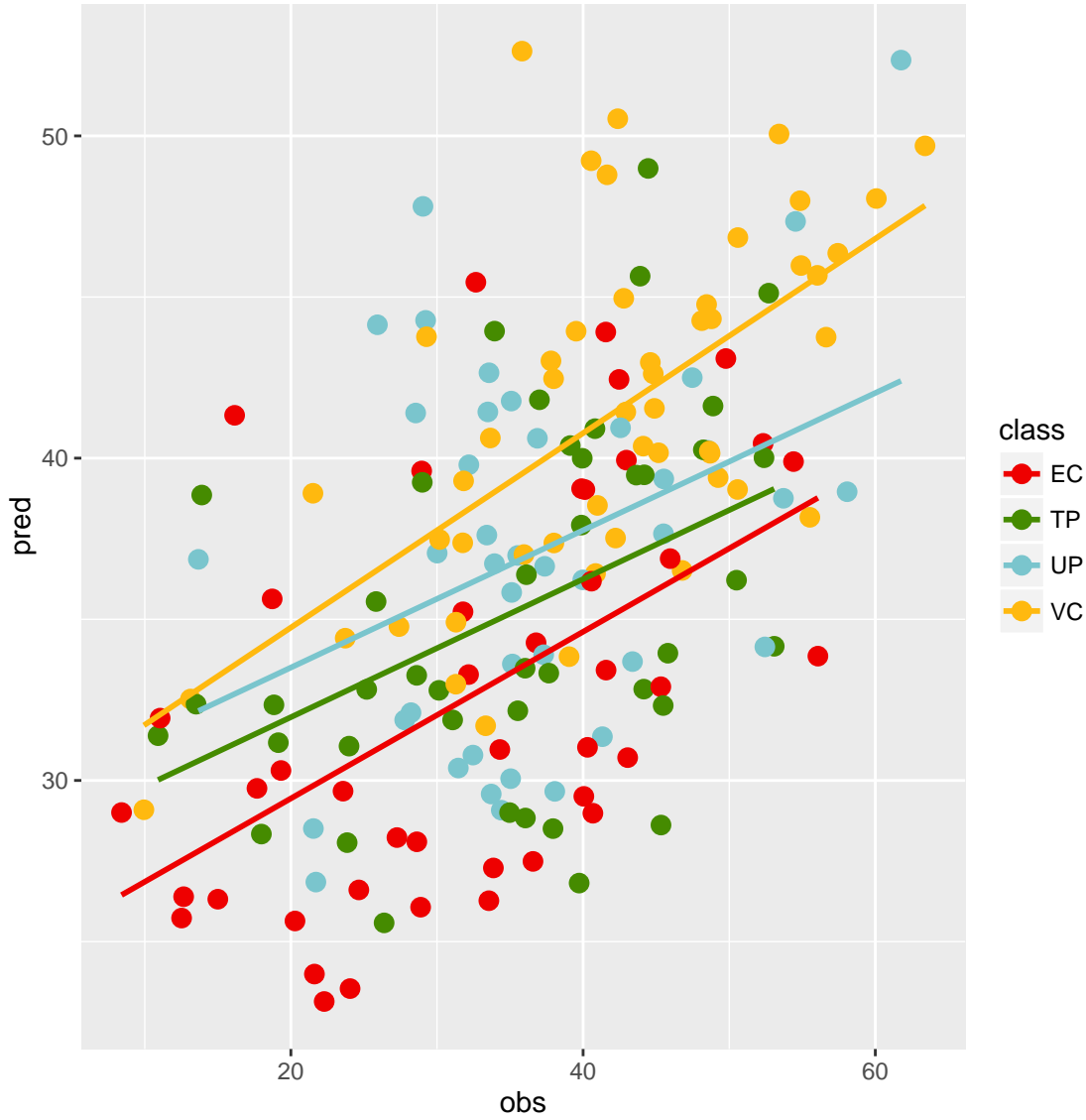

Supplement: Supplementary file 5 — Dataset EV3 [file MSB-14-e7881-s005.zip › dataset_EV3/titer/MIP1b/lambda.min/cvmod-scatter.pdf]

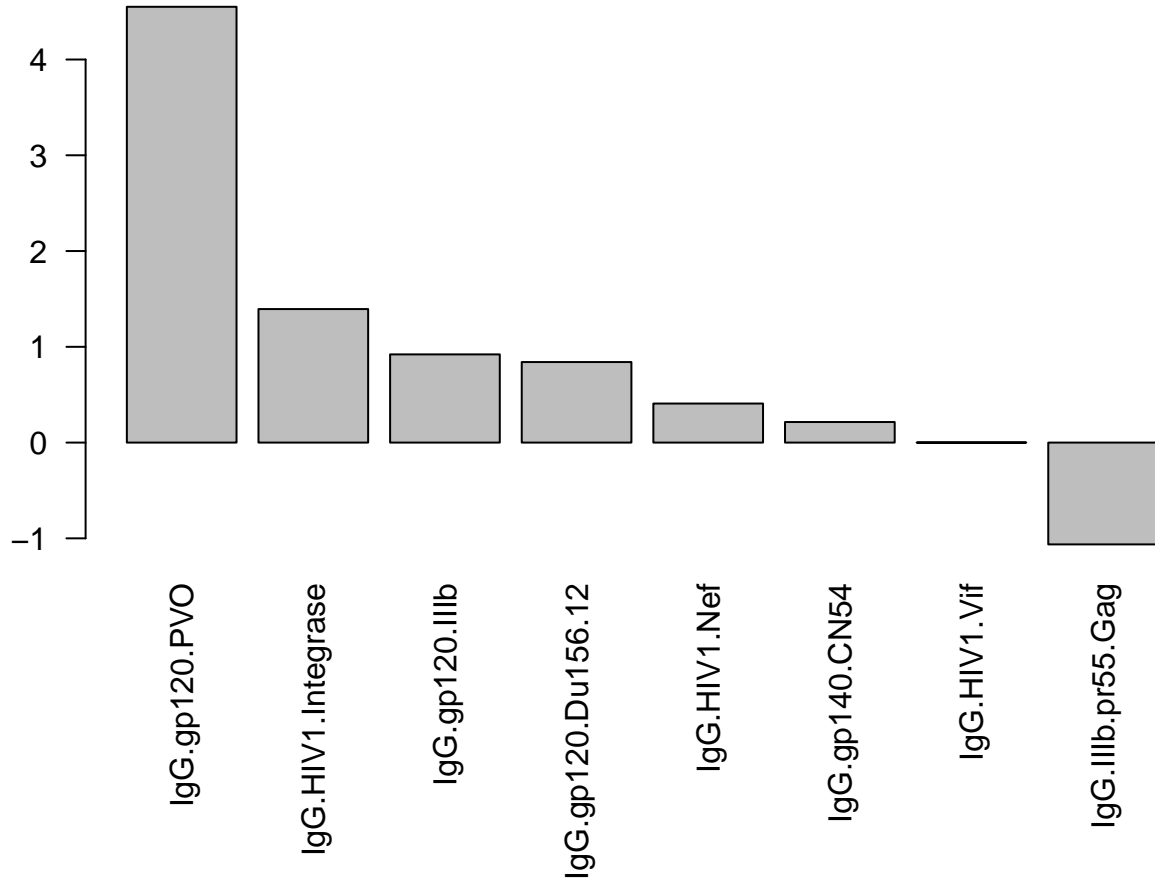

Supplement: Supplementary file 5 — Dataset EV3 [file MSB-14-e7881-s005.zip › dataset_EV3/titer/MIP1b/lambda.min/fullmod-coeffs.pdf]
